# Quantitative analysis of septin Cdc10 & Cdc3-associated proteome during stress response in the fungal pathogen *Cryptococcus neoformans*

**DOI:** 10.1371/journal.pone.0313444

**Published:** 2024-12-17

**Authors:** Stephani Martinez Barrera, Emma Hatchell, Stephanie D. Byrum, Samuel G. Mackintosh, Lukasz Kozubowski

**Affiliations:** 1 Department of Genetics and Biochemistry, Eukaryotic Pathogens Innovation Center, Clemson University, Clemson, SC, United States of America; 2 Department of Biochemistry and Molecular Biology, University of Arkansas for Medical Sciences, Little Rock, AR, United States of America; Duke University Medical Center: Duke University Hospital, UNITED STATES OF AMERICA

## Abstract

*Cryptococcus neoformans* is a pathogenic basidiomycetous yeast that primarily infects immunocompromised individuals. Fatal outcome of cryptococcosis depends on the ability of *C*. *neoformans* to sense and adapt to 37°C. A complex of conserved filament forming GTPases, called septins, composed of Cdc3, Cdc10, Cdc11, and Cdc12, assembles at the mother-bud neck in *C*. *neoformans*. Septins Cdc3 and Cdc12 are essential for proliferation of *C*. *neoformans* at 37°C and for virulence in the *Galleria mellonella* model of infection, presumably due to their requirement for septin complex formation, and the involvement in cytokinesis. However, how exactly Cdc3, and Cdc12 contribute to *C*. *neoformans* growth at 37°C remains unknown. Based on studies investigating roles of septins in *Saccharomyces cerevisiae*, septin complex at the mother-bud neck of *C*. *neoformans* is predicted to interact with proteins involved in cell cycle control, morphogenesis, and cytokinesis, but the septin-associated proteome in *C*. *neoformans* has not been investigated. Here, we utilized tandem mass spectrometry to define *C*. *neoformans* proteins that associate with either Cdc3 or Cdc10 at ∼25°C or after the shift to 37°C. Our findings unveil a diverse array of septin-associated proteins, highlighting potential roles of septins in cell division, and stress response. Two proteins, identified as associated with both Cdc3 and Cdc10, the actin-binding protein profilin, which was detected at both temperatures, and ATP-binding multi-drug transporter Afr1, which was detected exclusively at 37°C, were further confirmed by co-immunoprecipitation. We also confirmed that association of Cdc3 with Afr1 was enhanced at 37°C. Upon shift to 37°C, septins Cdc3 and Cdc10 exhibited altered localization and Cdc3 partially co-localized with Afr1. In addition, we also investigated changes to levels of individual *C*. *neoformans* proteins upon shift from ∼25 to 37°C in exponentially grown culture and when cells entered stationary phase at ∼25°C. Our study reveals changes to *C*. *neoformans* proteome associated with heat and nutrient deprivation stresses and provides a landscape of septin-associated *C*. *neoformans* proteome, which will facilitate elucidating the biology of septins and mechanisms of stress response in this fungal pathogen.

## Introduction

*C*. *neoformans* is a mycosis-causing opportunistic fungal pathogen, which is responsible for approximately 152,000 cases of cryptococcal meningitis, resulting in 112,000 global deaths annually [1]. Immunocompromised individuals, HIV patients, recent organ transplant recipients, and other patients who receive immunosuppressing drugs are particularly susceptible to cryptococcal infection [2]. Humans get infected by inhalation of spores or desiccated yeast cells found in the dust and/or avian feces [3]. During the initial stages of infection, *C*. *neoformans* employs antiphagocytic factors such as capsule and melanin to evade the alveolar macrophages [4, 5]. *C*. *neoformans* then proceeds to utilize phagocytosis as a trojan horse mechanism to disseminate into the central nervous system (CNS) [6, 7]. Understanding the mechanisms that *C*. *neoformans* employs to survive at host temperature (37°C) is a potential route for deciphering novel anti-cryptococcal drug targets. *C*. *neoformans* septin proteins Cdc3 and Cdc12 are a point of interest since they are essential for successful cytokinesis and survival at host temperature (37°C) while being dispensable for proliferation at 25°C [8].

Septins are a family of GTP-binding, filament-forming proteins that are conserved in eukaryotic cells, except for plants [9–11]. Moreover, septins have been recently recognized as a novel component of the cytoskeleton [12]. Septins assemble into hetero-oligomeric complexes that form higher-order structures such as filaments, gauzes, and rings at sites of cell division and active growth [11, 13]. Septins were first identified in the baker’s yeast, *Saccharomyces cerevisiae*, as crucial factors for the separation of the daughter from the mother cell in mitotically proliferating cells [13–15]. In *S*. *cerevisiae*, five septin proteins, Cdc3, Cdc10, Cdc11, Cdc12, and Shs1 (which is most similar to Cdc11) assemble into hetero-oligomeric complexes, which polymerize into filaments at the presumptive bud site. Upon budding, septin filaments are organized into an hourglass-shaped collar at the mother–bud neck. During mitotic exit, prior to cytokinesis, septin-based collar splits into a double ring by the mechanism that remains poorly understood [12, 15, 16]. *S*. *cerevisiae* septins Cdc3 and Cdc12 are essential, presumably due to their requirement for septin complex assembly and the essential role of the complex in cytokinesis [14, 17–20]. *S*. *cerevisiae* cells lacking cdc10 are viable and form a complex consisting of the remaining septins that fails to split into the double ring during cytokinesis, suggesting that ring splitting is not essential for cytokinesis [21–23]. Beyond cytokinesis, *S*. *cerevisiae* vegetative septins also control cell polarity and bud morphogenesis, while specialized developmentally regulated septins, Spr3 and Spr28, contribute to sexual development and sporulation [13, 24–26].

Septin proteins have been shown to associate with negatively charged membrane surfaces and have an affinity for binding to a variety of phospholipids, specifically phosphoinositides [27]. It has also been determined that membrane association promotes septin assembly into filaments; therefore, the association of septins with membranes might be facilitated by the assembly of septins into higher-order structures [28]. Septins also associate with positively charged membranes. Nonetheless, in this case, septins have been found to sense on the microscale the positive membrane curvature [29]. This capability is a staple of proteins involved in intracellular membrane trafficking [29]. During the past decade, several novel septin roles have been elucidated in mammalian cells including interacting with components of endocytosis and exocytosis [30, 31], acting as membrane diffusion barriers [32, 33], interacting with proteins involved in cytoskeleton organization [34, 35], and interacting with proteins that are functionally associated with the ubiquitin and sumoylation cycles [34, 36]. In addition, septins were recently also associated with autophagy in *S*. *cerevisiae* [37, 38]. Thus, septins are conserved proteins involved in multiple cellular pathways in eukaryotic organisms.

Numerous studies have reported a plethora of proteins that localize to the mother-bud neck in *S*. *cerevisiae* in a septin-dependent manner and act in polarity establishment, cell cycle regulation, cell wall biogenesis/chitin deposition, and cytokinesis [14]. A previous global quantitative analysis of the septin Cdc11-associated proteome in *S*. *cerevisiae* revealed 83 putative interacting partners in pathways such as ribosomal biogenesis, cell cycle, and endocytosis [39]. *C*. *neoformans* septin interactome had not yet been thoroughly analyzed for potential interacting partners. Given that septins Cdc3 and Cdc12 are not necessary for viability at 25°C in *C*. *neoformans* and they become essential at 37°C or during other types of stress [8], septins may play unique stress-related functions in *C*. *neoformans* that may be also conserved in other eukaryotes.

In this study, tandem-mass spectrometry was utilized to identify proteins that associate with septins Cdc3 and Cdc10 in *C*. *neoformans* during nutrient-rich growth at ∼25°C, and after the temperature shift to 37°C. This study has also revealed changes to *C*. *neoformans* proteome when exponentially grown cells are shifted from ∼25 to 37°C and when cells enter stationary phase of growth at ∼25°C. Thus, findings presented here should facilitate elucidating the biology of septins and the mechanisms of stress response in *C*. *neoformans*.

## Materials and methods

### *C*. *neoformans* strains

All *C*. *neoformans* strains included in this study were derived from *C*. *neoformans* wild-type reference strain H99 (*MAT*⍺) and are listed in Table 1. The strain expressing Cdc10-mCherry (LK60) and the strain expressing Cdc3-mCherry (LK140) were generated as previously described [8]. The wild-type H99 strain was used as a negative control.

**Table 1:** List of strains and plasmids utilized in this study.

Strain	Genotype	Source/Reference
H99	(⍺) WT	(Perfect, et al. 1993) [43]
LK60	(⍺) *CDC10-mCherry*: *NEO*	(Kozubowski & Heitman, 2010) [8]
LK140	(⍺) *CDC3-mCherry*: *NEO*	(Kozubowski & Heitman, 2010) [8]
SM1	(⍺) *GFP-PROFILIN (CNAG_00584)*: *HYG*	This study
SM2	(⍺) *GFP- PROFILIN (CNAG_00584)*: *HYG Cdc10-mCherry*: *NEO*	This study
SM3	(⍺) *GFP-AFR1 (CNAG_00730)*: *HYG*	This study
SM4	(⍺) *GFP-AFR1 (CNAG_00730)*:*HYG Cdc10-mCherry*: *NEO*	This study
**Plasmid**	**Genotype**	**Source/Reference**
pLKB90	pXLI + *GFP* (*HYG*)	Based on previously described pLK55 [40]
pSM1	*GFP*-*PROFILIN* (CNAG_00584): *HYG*	This study
pSM2	*GFP*-*AFR1* (CNAG_00730): *HYG*	This study

All strains expressing N-terminally tagged green fluorescent protein (GFP) were generated as previously described [8]. Strains expressing GFP-profilin and GFP-Afr1 were made by ectopic integration of plasmids, based on pLKB90 encoding GFP expressed from the GPD1 promoter. Plasmid pLK90 is identical to previously described pLK55 [40], except it has replaced mCherry-encoding sequence by GFP-encoding sequence. Genes of interest were amplified via PCR and restriction cloning was used to generate cloned plasmids. To amplify profilin ORF (CNAG_00584) to generate pSM1, the following primers were used: forward primer with NheI restriction site added at 5’ end (ACAGCTAGCCatgtcctggcaaggtac), and reverse primer with PacI restriction site added at 3’ end (CAGTGTTAATTAAgtgaagacggaactgg). To amplify *AFR1* ORF (CNAG_00730) to generate pSM2, the following primers were used: forward primer with NheI restriction site added at 5’ end (TCAGCTAGCaatgtcagctgcaggcgt), and reverse primer with PacI restriction site added at 3’ end (AGTCTTAATTAAcatcgcttgagacgatcagg). The plasmids generated for this study were confirmed via sequencing (S1 and S2 Data). The integrations were conducted via biolistic transformation, as described previously [41, 42]. The positive clones were screened based on growth media containing Hygromycin 300 μg/mL. In addition, the clones were confirmed by examining the fluorescent signal.

### Media and growth conditions

Unless otherwise stated, *C*. *neoformans* strains were routinely maintained in YPD medium (2% yeast extract, 1% bacto-peptone, 2% dextrose, 2% bacto-agar) at ∼25°C.

To collect samples for the MS analysis, strains LK60, LK140, and H99(⍺), were initially cultured for 24 h in 1000 mL YPD media at ∼25°C (cells grown to mid-exponential phase; OD_600_ ≤ 1). Following this, each 1000 mL culture was divided into two 500 mL cultures. For analysis of cells approaching the stationary phase of growth, one of the divided cultures continued incubation in the same medium for an additional 48 h at ∼25°C. For analysis of cells at exponential phase of growth, the other 500 mL cultures were refreshed in YPD media (cell density ∼ 10^5^ cells/mL) and grown for 3 hours at ∼25°C. Next, the 500 ml culture was divided into two 250 ml cultures (cell densities ∼ 10^7^ cells/ml). One of the divided cultures was incubated at ∼25°C and the other at 37°C for 2 h. All cultures were then used to prepare cell lysates, as described in the next section.

### Preparation of the cell lysates

After the final incubations (described in the previous section), the cultures representing each experimental condition were rapidly chilled using dry ice and independently centrifuged at 5,000 x g for 20 mins at 4°C. Cells were then re-suspended in 30 ml of ice-cold lysis buffer (10 mM Tris/Cl pH  =  7.5, 150 mM NaCl, 13% (v/v) glycerol, 0.5 mM EDTA) supplemented with protease inhibitor tablets (Roche cOmplete™, Mini, EDTA-free, Protease Inhibitor Cocktail, Millipore Sigma, Sigma-Aldrich) and 1 mM PMSF. The re-suspended cell pellets were aliquoted in 1 ml microcentrifuge tubes with screw caps and disrupted using ∼500 μl of 0.5 mm glass beads (Sigma-Aldrich) in a Mini-Beadbeater (Biospec Products). Samples were homogenized at 4°C for 20 sec, followed by 1 min incubations on ice. This process was repeated 8 times to ensure that a sufficient level of cell disruption had been achieved (initially confirmed by direct microscopic observation). Afterward, the lysate of disrupted cells from each condition was centrifuged at 1200 × g for 10 min at 4°C to remove large cell debris. The supernatant was subsequently collected and stored at −80°C until use.

### Anti-mCherry immunoprecipitation & GeLC-MS/MS (Orbitrap Fusion)

The RFP-TRAP affinity resin coupled with RFP Nanobody/VHH from ChromoTek® agarose beads were employed for immunoprecipitation of the fluorescently tagged bait protein, as these beads are well-suited for mCherry tagged fluorescent bait proteins with no contamination concerns from heavy or light chain polypeptide chains in the elution fractions (https://www.ptglab.com/products/pictures/pdf/rfp-trap_brochure.pdf).

The lysate from each experimental condition was thawed on ice. The relative protein expression levels in the lysate were ascertained as a control as previously described [44]. For analysis of proteins associated with Cdc10-mCherry and Cdc3-mCherry, respectively, in every experimental condition, ∼5 ml of lysate was incubated for 4 h with slow end-over-end mixing at 4°C with 50 μl of ChromoTek RFP-Trap® agarose bead slurry, prepared according to manufacturer instruction. After the 4 h incubation, the ChromoTek RFP-Trap® agarose beads were washed 3× with 500 μl of wash buffer (TBS with 2 M urea, pH 7.5). Next, the wash buffer was removed and the ChromoTek RFP-Trap® agarose beads were resuspended in 100 μl of 2x electrophoresis sample buffer. The samples were then boiled for 10 min at 95°C to dissociate the immunocomplexes from the beads. Then, the eluted fraction was removed and collected via centrifugation at 2,500 × g for 2 min at 4°C. The eluted sample (supernatant) was then resolved on SDS-PAGE gel and stained with Coomassie blue. Each SDS-PAGE gel lane was sectioned into 12 segments of equal volume. Each segment was subjected to in-gel trypsin digestion as described previously [44]. Of note, the in-gel digestion included the entire gel lane and therefore did not omit the bait protein or other hypothetical proteins that might have migrated like the bait protein. Gel slices were destained in 50% methanol (Fisher), and 50 mM ammonium bicarbonate (Sigma-Aldrich), followed by a reduction in 10 mM Tris[2-carboxyethyl] phosphine (Pierce) and alkylation in 50 mM iodoacetamide (Sigma-Aldrich). Gel slices were then dehydrated in acetonitrile (Fisher), followed by the addition of 100 ng porcine sequencing grade modified trypsin (Promega) in 50 mM ammonium bicarbonate (Sigma-Aldrich) and incubation at 37°C for 12–16 h. Peptide products were then acidified in 0.1% formic acid (Pierce). Tryptic peptides were separated by reverse phase XSelect CSH C18 2.5 μm resin (Waters) on an in-line 120 x 0.075 mm column using a nanoAcquity UPLC system (Waters). Peptides were eluted using a 45 min gradient from 98:2 to 67:33 buffer A: B ratio. [Buffer A = 0.1% formic acid, 0.5% acetonitrile; buffer B = 0.1% formic acid, 99.9% acetonitrile.] Eluted peptides were ionized by electrospray (2.2 kV) followed by MS/MS analysis using higher-energy collisional dissociation (HCD) on an Orbitrap Fusion Tribrid mass spectrometer (Thermo) in top-speed data-dependent mode. MS data was acquired using the FTMS analyzer in profile mode at a resolution of 240,000 over a range of 375 to 1500 m/z. Following HCD activation, MS/MS data was acquired using the ion trap analyzer in centroid mode and normal mass range with precursor mass-dependent normalized collision energy between 28.0 and 31.0 [45, 46].

### Data analysis

Proteins were identified using EncyclopeDIA [47] against the *C*. *neoformans* H99 UniProtKB database (Proteome ID: UP000010091) (Database last modified: February 15, 2023) [48]. Verification of MS/MS-based peptide and protein identifications was done using Scaffold DIA (Proteome Software), adhering to stringent false discovery rate criteria of 1% at both the protein and peptide levels.

The exclusive MS2 intensity data in Scaffold DIA was imported to RStudio to be analyzed by the R package proteoDA for quantitative proteomics [49]. Protein MS2 exclusive intensity values were assessed for quality using ProteiNorm [52]. The data were normalized using cyclic loess [50] and analyzed using proteoDA to perform statistical analysis using Linear Models for Microarray Data (limma) with empirical Bayes (eBayes) smoothing to the standard errors [49, 50]. Proteins with an FDR adjusted p-value < 0.05 and a fold change > 2 were considered significant. Using this method, quantitative analysis between the ∼25°C and 37°C experimental conditions was performed. This approach was employed to determine whether or not there is differential binding between these two experimental conditions for each binding category.

### Heat map generation

The exclusive dataset derived from each of the five biological replicates from the bulk protein cell lysate of the wild type H99 control under three varying conditions: ∼25°C (exponential growth phase), ∼25°C (stationary growth phase), and 37°C, was subjected to analysis. A heat map was generated utilizing Heatmapper [51] to visually illustrate protein expression variations across these conditions. The heatmap was constructed employing average clustering, coupled with Kendall’s Tau distance measurement method, to accurately discern and represent the hierarchical relationships in protein expression.

### Gene Set Enrichment Analysis (GSEA)

The differentially expressed proteins across the defined conditions were subjected to Gene Set Enrichment Analysis (GSEA) using the Blast2GO function within OmicsBox software to pinpoint significant gene sets correlating with various biological processes, molecular functionalities, and cellular components. This method of analysis follows the knowledge-based approach for interpreting genome-wide expression profiles as delineated by Subramanian et al. [52]. For this analysis, a False Discovery Rate (FDR) of 0.25 was employed instead of the more traditional 0.05. The rationale behind this choice lies in the exploratory nature of this study, where the primary aim is to formulate hypotheses for subsequent validation in future research. Given the intrinsic lack of coherence in most expression datasets and the relatively compact number of gene sets being examined, a more stringent FDR cutoff might lead to the omission of potentially significant results. The utilization of an FDR of 0.25 ensures a reasonable balance, allowing for the identification of valid results 3 out of 4 times, thereby broadening the scope of uncovering relevant findings. The discernment of significant gene sets elucidates the underlying biological narratives amidst the protein expression alterations, enabling a holistic understanding of the systemic impacts upon *C*. *neoformans* H99 (wild type) under differing environmental conditions.

### Functional Enrichment Analysis

The identified binding partners of septins Cdc3 and Cdc10, obtained via mass spectrometry, were subjected to Functional Enrichment Analysis using the Blast2GO function within OmicsBox software. For gene enrichment analysis, Fisher’s Exact Test was employed as per the methodology elucidated by Al-Shahrour et al. [53]. This analysis facilitated the demarcation of predominant biological processes, molecular functions, and cellular components associated with these binding partners, thereby further elucidating the cellular roles and implications of septins Cdc3 and Cdc10. By exploring the enriched functional annotations and gene ontology terms, a comprehensive understanding of the functional landscape in which septins Cdc3 and Cdc10 operate was achieved, granting insights into their potential biological significance and interactions within the cellular milieu.

### *In Vivo* co-immunoprecipitation

Cells were cultured for 24 h in 500 mL YPD media at ∼25°C. Subsequently, cell cultures were refreshed in YPD media (cell density ∼ 10^5^ cells/mL) and grown for 3 hours at ∼25°C. Next, each 500 ml culture was divided into two 250 ml cultures (cell densities ∼ 10^7^ cells/ml). One culture from each strain was incubated at ∼25°C and the other at 37°C. After a 2 h incubation, cell cultures from each experimental condition were rapidly chilled using dry ice and independently centrifuged at 5,000 x g for 20 mins at 4°C. The centrifuged cell pellets from each experimental condition were then subject to cell lysis according to the protocol utilized for the MS analysis (described above). For analysis of proteins associated with Cdc10-mCherry and Cdc3-mCherry, respectively, in every experimental condition, ∼5 ml of lysate was incubated for 4 h with slow end-over-end mixing at 4°C with 25 μl of either ChromoTek RFP-Trap®, or ChromoTek GFP-Trap® agarose bead slurry, prepared according to manufacturer manual. After the 4 h incubation, the agarose beads were washed 3× with 500 μl of wash buffer (TBS with 2 M urea, pH 7.5). Next, the wash buffer was removed, and the agarose beads were resuspended in 80 μl of 2x electrophoresis sample buffer. The samples were then boiled for 10 min at 95°C to dissociate the immunocomplexes from the beads. Then, the eluted fraction was collected via centrifugation at 2,500 × g for 2 min at 4°C. The eluted sample (supernatant) was then resolved on SDS-PAGE gel. Proteins were transferred to nitrocellulose membranes, which were then blocked with 5% nonfat dry milk in tris-buffered saline (TBS) containing 0.1% Tween 20 (TBS/Tween). To detect GFP chimeras via western blotting, an anti-GFP monoclonal antibody (3H9, Chromotek®) was used at a 1:1,000 dilution and incubated overnight at 4°C, followed by HRP-conjugated Affinipure Goat Anti-Rat IgG(H+L) (SA00001-15, Proteintech®) at a dilution of 1:2000, incubated at room temperature for 1 h. To detect mCherry chimeras, an anti-RFP monoclonal antibody (6G6, Chromotek®) was used at a 1:1,000 dilution and incubated overnight at 4°C, followed by HRP-conjugated Affinipure Goat Anti-Mouse IgG(H+L) (SA00001-1, Proteintech®) at a dilution of 1:10000, incubated at room temperature for 1 h. Subsequently, blots were developed with Pierce™ ECL Plus Western Blotting Substrate (32132, Thermo Scientific™) and imaged using the Analytik Jena UVP Chem Studio. Raw data of western blot images related to Fig 10 are available as a supplementary file (S1 Raw images).

### Microscopy

For microscopy, cells were grown overnight in YPD liquid medium at ∼25°C and refreshed the following morning using a 1:10 dilution. Cells were then counted and diluted to achieve a concentration of 1×10^6^ cells/ml. Subsequently, 200–400 μl of cell suspension was aliquoted into a microslide glass chamber (μ-Slide 2 Well Glass Bottom; Ibidi-cells in focus) for imaging. Brightfield, differential interface microscopy (DIC), and fluorescence images were captured with Leica GDSF/TIRF inverted microscope. Images were processed through ImageJ; Fiji software [54, 55].

For cells imaged at 37°C, cultures were initially grown overnight as described above. After refreshing the cultures the following morning using a 1:10 dilution, cells were grown at 37°C for two hours. Subsequently, the cultures were diluted to achieve a concentration of 1×10^6^ cells/ml. Next, 200–400 μl of cell suspension was aliquoted into a pre-heated (37°C) microslide glass chamber (μ-Slide 2 Well Glass Bottom; Ibidi-cells in focus) for imaging. Brightfield, differential interface microscopy (DIC), and fluorescence images were captured with Leica GDSF/TIRF inverted microscope with the incubator chamber set to 37°C during the entire imaging session. Images were processed as described above.

## Results

### Experimental setup

We had initially hypothesized that cell cycle-related proteins would be among the candidate septin-binding partners identified in this study, given the conserved localization of septins in *C*. *neoformans* to the site of cell division [8]. We also anticipated our analysis revealing differences in the septin binding partners between *S*. *cerevisiae* and *C*. *neoformans* due to the following reasons: [1] septin proteins are essential for growth in *S*. *cerevisiae* at non-stress conditions, while they are dispensable for growth in *C*. *neoformans* only at non-stress conditions; [2] there is significant phylogenetic divergence between these two species.

According to literature based on the *S*. *cerevisiae* model and a study performed in *C*. *neoformans*, septins in *C*. *neoformans* function as a complex that consists of all four septin proteins present in *C*. *neoformans* genome (Cdc3, Cdc10, Cdc11, and Cdc12). While Cdc10 is dispensable for growth at 37°C, the remaining three septins are essential for growth at host temperature [8]. Furthermore, the septin complex does not form in the absence of Cdc3 or Cdc12 [8]. Since Cdc10 is presumably dispensable for septin complex formation, it was chosen as one of the “baits” for the pull down, as tagging this protein should have a minimal impact on septin complex functionality. Furthermore, Cdc10 was chosen since it has the highest homology to its *S*. *cerevisiae* septin counterpart.

As part of the initial study design, we planned to determine the binding partners of septin Cdc10 in the absence of Cdc3, conditions at which functional higher order septin complex does not form [8]. This approach was intended to identify Cdc10 interactions that could be independent of the formation of the whole septin complex. However, we could not successfully pull-down septin Cdc10 in the absence of Cdc3 and therefore did not follow this initial plan.

Due to septin Cdc3’s presumed critical role in septin ring homeostasis, it was chosen as a second “bait” for the tandem mass spectrometry. Identifying the interacting proteins of two septin proteins provided better insight into their functional roles and involvement in cellular pathways.

Studies in other organisms point to roles for septins that are independent of their established function during cytokinesis [34, 39, 56], and we anticipated that our findings may reveal septin interactions supporting this possibility. Specifically, we initially planned to utilize cultures grown to the stationary phase of growth, predicted to consist of mostly unbudded cells. We reasoned that unbudded cells would represent interactions with septins that are not relevant to cytokinesis. Unfortunately, we could not obtain a sufficient quantity of pulled-down fluorescently tagged septins when cells were in stationary phase. However, as the cells in the stationary phase cultures are expected to be undergoing starvation due to limited nutrient availability, we nonetheless utilized those samples to test the impact of nutrient deprivation stress on the whole *C*. *neoformans* proteome.

The bulk protein lysate from each sample was analyzed via mass spectrometry for the following two reasons: 1. To compare the whole proteome of *C*. *neoformans* grown at no-stress conditions with the proteome of cultures grown under high temperature stress or nutrient deprivation stress, 2. To provide a background control of protein levels for differential analysis of proteins associated with the septins. Consequently, label-free proteomic profiling of the differentially expressed proteins in cells exposed to heat stress (37°C) and nutrient starvation stress (stationary phase) as compared to no-stress control was performed and those data served also as a control for the analysis of the septin-associated proteome.

**Fig 1:**
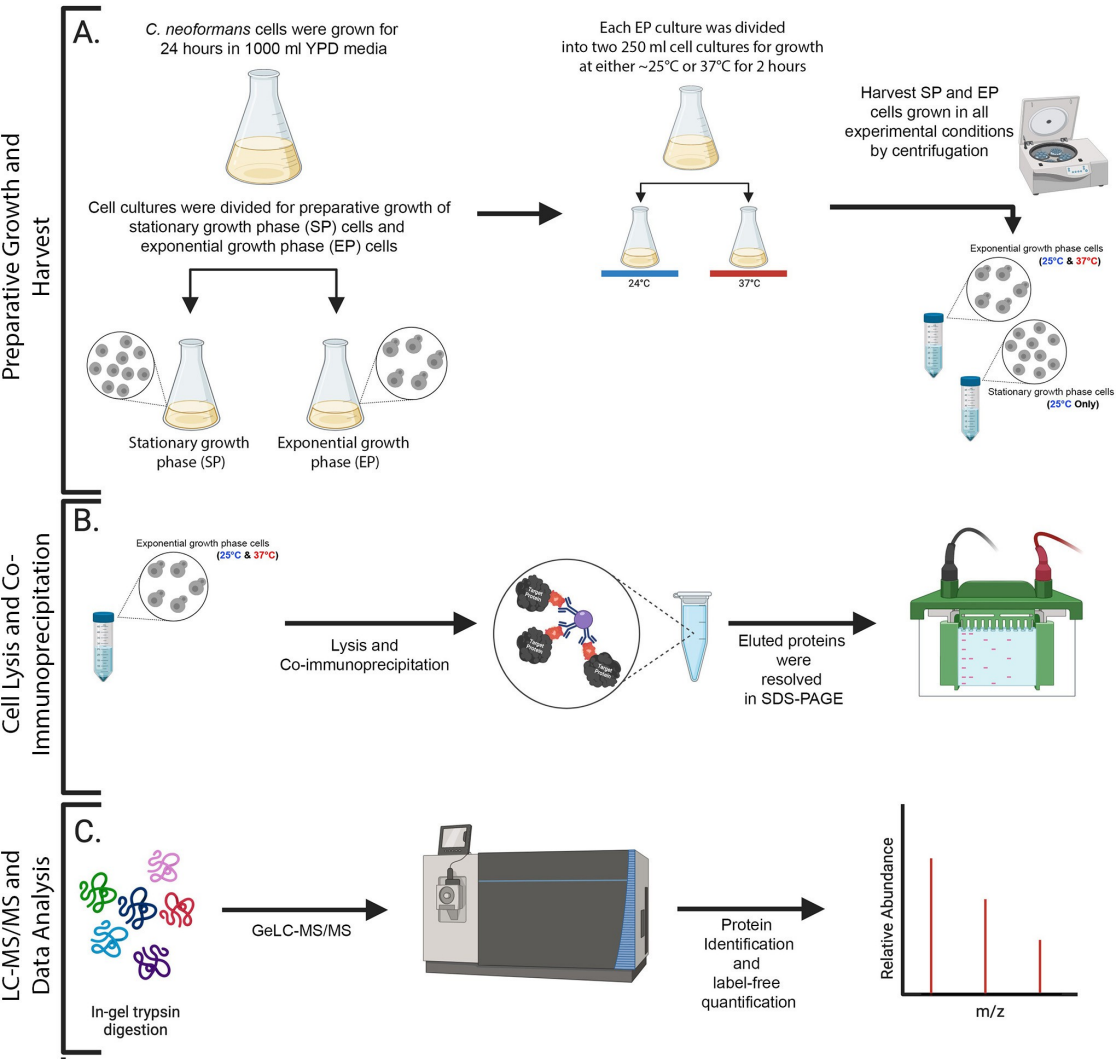
Schematic representation of the GeLC-MS/MS workflow. (A) *C*. *neoformans* cells were grown for 24 h in YPD rich media at ∼25°C. Subsequently, the cell culture was split, and half of the culture was further grown in the same media for an additional 48 h at ∼25°C (to ensure it approached the stationary phase of growth (SP)), while the other half was refreshed in YPD media and grown for 3 h at ∼25°C (to assure it was at an exponential phase of growth (EP)). Each EP culture was then divided into two cultures and grown either at ∼25 or at 37°C for 2 h before harvesting the cells. (B) Cell lysis and immunoprecipitation of bait protein using RFP-TRAP resin. Bound proteins were eluted and resolved on SDS-PAGE gel. (C) Each lane of the SDS-PAGE gel was excised into twelve fragments and subjected to in-gel trypsin digestion and analyzed by in-gel liquid chromatography-tandem mass spectrometry (GeLC-MS/MS) on an Orbitrap Fusion Tribrid mass spectrometer (Thermo). Raw data was processed with Scaffold DIA.

**Fig 2:**
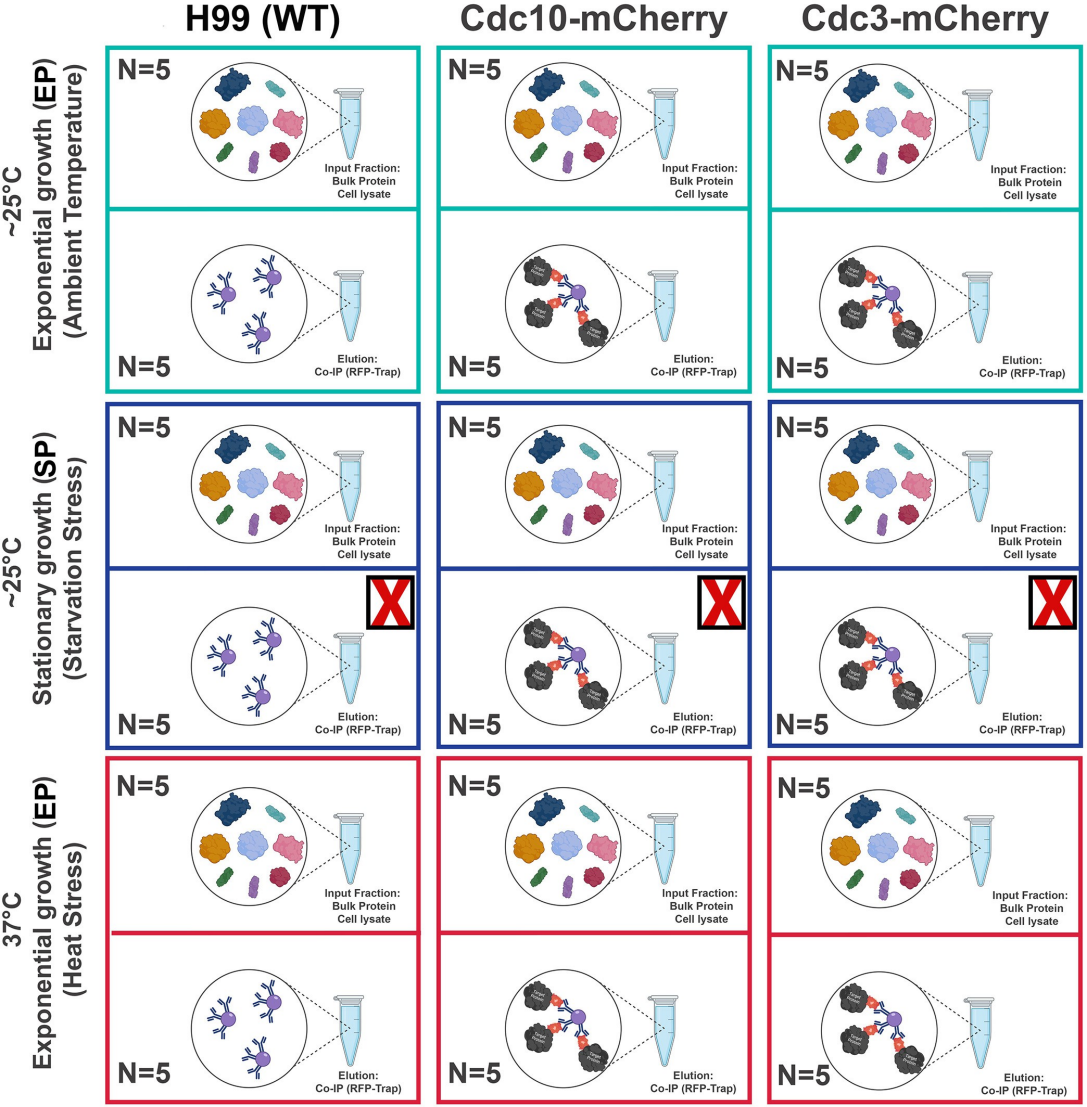
Schematic representation of samples that were analyzed via label-free quantitative mass spectrometry. The bulk protein cell lysate and subsequent co-immunoprecipitation elution fractions from Cdc3-mCherry and Cdc10-mCherry pulldowns of the following strains were analyzed via mass spec: H99 (WT), Cdc10-mCherry, and Cdc3-mCherry. The experiment was conducted under three independent conditions (N = 5): [1] exponential growth phase at ∼25°C (Ambient Temperature), [2] stationary growth phase at ∼25°C (Nutrient deprivation stress), and [3] exponential growth phase at 37°C (Heat Stress).

A schematic representation of the the GeLC-MS/MS workflow is depicted in Fig 1. A total of 9 experimental samples (Table 2), and 9 control samples, were collected for mass spectrometry analysis (Fig 2). The control samples were the bulk protein lysate (input) of each experimental pulldown condition. The following two comparisons were performed to gain information regarding the differentially expressed proteome of *C*. *neoformans* strain H99 (WT) during the exposure to two stress conditions: [1] Comparison of the bulk protein cell lysate obtained from cells grown exponentially at ∼25°C vs. the lysate obtained from cells incubated at 37°C (Heat Stress); [2] Comparison of the bulk protein cell lysate obtained from cells grown exponentially at ∼25°C vs. the lysate obtained from stationary growth phase culture grown at ∼25°C (Nutrient deprivation stress).

**Table 2:** Samples collected for the subsequent MS analysis (N = 5).

	Strain		
Experimental condition	H99	LK60	LK140
25°C, SP	#1	#2	#3
25°C, EP	#4	#5	#6
37°C, EP	#7	#8	#9

### Global proteomic profiling: Changes to *C*. *neoformans* protein levels due to low nutrient availability

**Fig 3:**
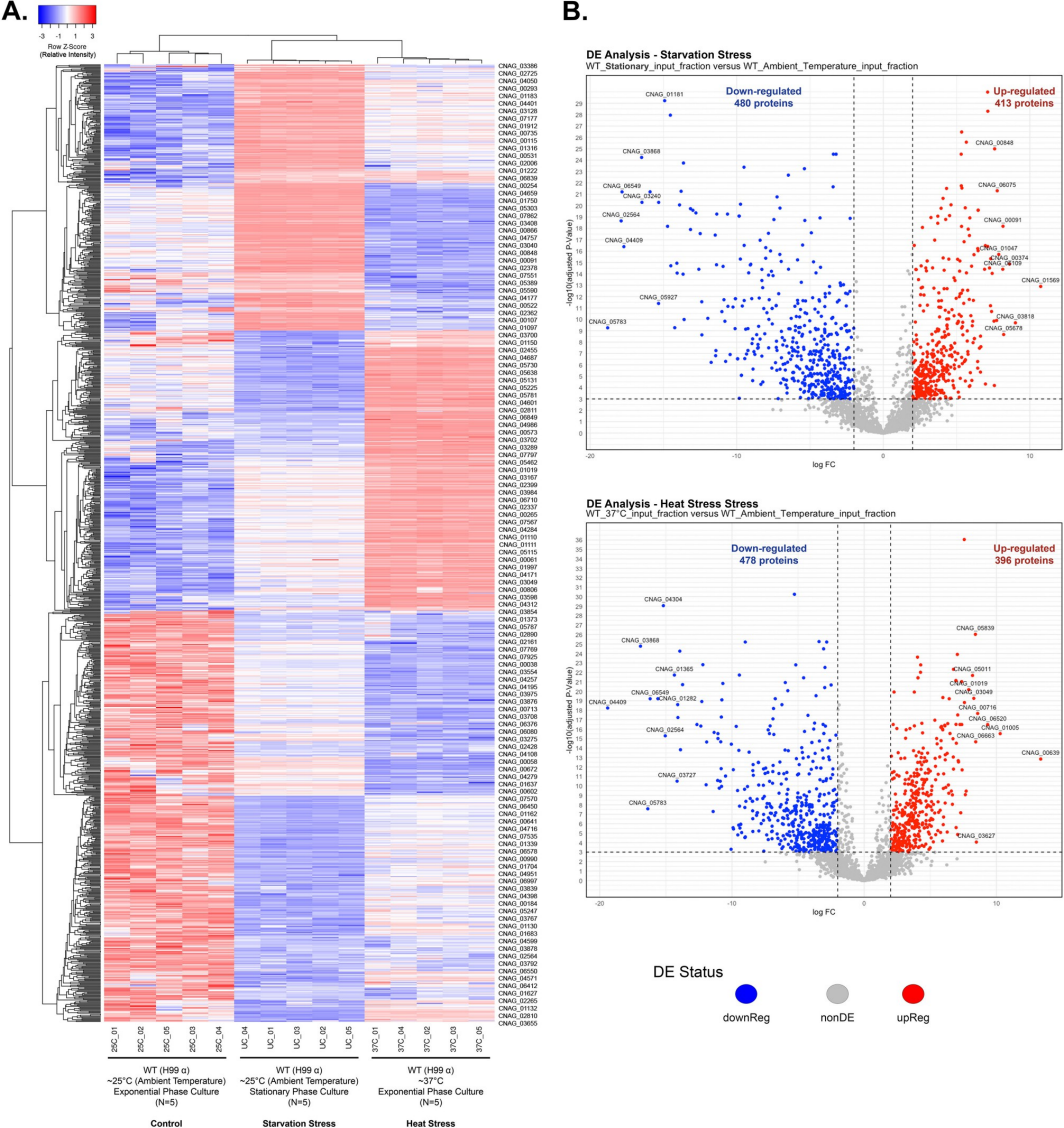
Heat stress and starvation stress associated proteome changes in *C*. *neoformans* revealed by label-free quantitative proteomics. (A) Heat map representation of differentially expressed proteins across the following conditions: ∼25°C during the exponential growth phase (control), ∼25°C during the stationary growth phase (starvation stress), and 37°C during the exponential growth phase (heat stress). The Log2 Cyclic Loess Normalized Exclusive Intensities of each protein per sample were transformed to Z-scores. Clustering was performed both on proteins and samples using an average linkage method and Kendall’s Tau distance measurement method. Upregulated and downregulated proteins are represented with red and blue colors, respectively. (B) Top: Volcano plot displaying results of differential expression analysis comparing proteome of control (∼25°C during exponential growth phase), and nutrient starvation stress (∼25°C during stationary growth phase) proteome. Bottom: Volcano plot displaying results of differential expression analysis comparing proteome of control (∼25°C during exponential growth phase), and heat stress (37°C during exponential growth phase) proteome. The dashed vertical lines differentiate the upregulated and downregulated proteins (absolute value FC > 2). The proteins above the dashed horizontal line represent the statistically significant proteins (BH-adjusted p < 0.001). Proteins highlighted in red are significantly upregulated, and proteins highlighted in blue are significantly downregulated.

The analysis of the bulk protein lysates from all the samples served as a background protein content in the input fractions that was critical in evaluating the septin interactome. The analysis of the bulk protein cell lysates from the wildtype strain H99 allowed to compare *C*. *neoformans* proteome between no stress and two stress conditions, nutrient deprivation, and high temperature. This analysis revealed that the proteome is differentially expressed both during heat stress at 37°C and during nutrient starvation associated with the stationary phase growth (Fig 3A). Changes to protein abundance with a log_2_FC >2 or <-2 (with adjusted p-value of 0.001) were considered significantly upregulated or downregulated, respectively.

**Fig 4:**
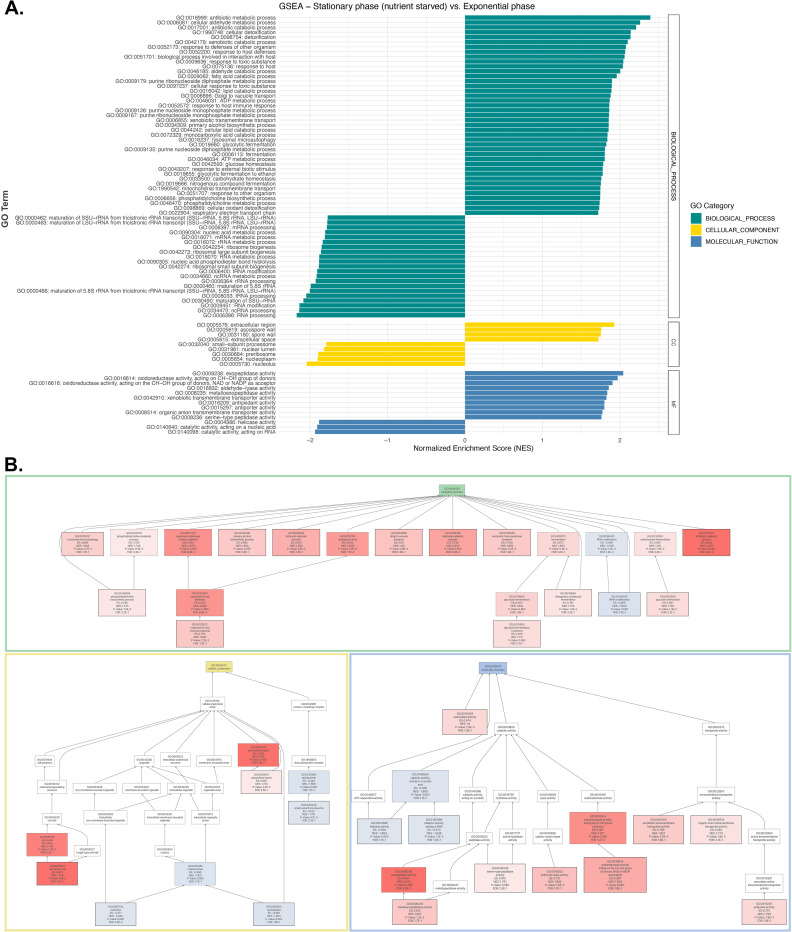
Gene set enrichment analysis (GSEA) of differentially expressed proteome in stationary growth phase versus exponential growth phase. (A) Bar plot for significantly enriched gene ontology (GO) categories with p-value < 0.05 and FDR < 0.25 from GSEA. To avoid overlooking potentially significant gene ontology terms, an FDR of 0.25 rather than 0.05 was used for GSEA. (B) Directed acyclic graph of enriched GO terms with GSEA. The GO terms with positive enrichment are highlighted in red; while the GO terms with negative enrichment are highlighted in blue.

Differential expression analysis revealed 413 significantly upregulated and 480 significantly downregulated proteins during the stationary versus exponential growth phase (Fig 3B) (Table 3). Gene set enrichment analysis (GSEA) demonstrated that glucose homeostasis, antibiotic catabolic process, fermentation, response to host defense, fatty acid catabolic process, lysosomal autophagy, and phosphatidylcholine biosynthetic process were among the biological processes that were positively enriched during stationary phase growth when compared to exponential culture (Fig 4A and 4B). In contrast, the biological process of tRNA modification had a negative enrichment score indicating that protein synthesis might be decreased during the stationary growth phase. Consistent with this possibility, the pre-ribosome was one of the cellular compartments with a negative enrichment score during stationary phase growth. In contrast, the extracellular region and ascospore wall compartments were among the cellular compartments with a positive enrichment score. This might be indicative of the importance of cell wall integrity during stress response (Fig 4A and 4B). Likewise, the claim that protein synthesis-related pathways might be suppressed during stationary phase growth is further supported by the significant negative enrichment score for the molecular function of catalytic activity on RNA and nucleic acid. Nonetheless, the catalytic activity on proteins, specifically exopeptidase activity was a positively enriched molecular function. This could indicate that protein synthesis is not completely compromised due to downregulation in tRNA modification proteins. Additionally, antioxidant activity, oxidoreductase activity, and transporter activity were also among the molecular functions with positive enrichment in stationary phase stressed cells (Fig 4A and 4B).

**Table 3:** Differentially expressed proteins in *C*. *neoformans* WT (H99) cultures exposed to either heat stress or nutrient deprivation. (The top 30 candidates of each condition are summarized in this table, the complete dataset can be found in the S3 Data).

Uniprot ID	Gene ID	Protein Description	MW (kDA)	logFC	P.Value	adj.P.Val
***Stationary Phase (Nutrient Starvation)***						
**J9VYP4**	CNAG_01569	Mitochondrial import inner membrane translocase subunit Tim21	38 kDa	10.7386168	6.9315E-15	1.2773E-13
**J9VL35**	CNAG_03818	Osomolarity two-component system, response regulator SSK1	140 kDa	9.01354323	1.9441E-11	2.0262E-10
**J9VIV5**	CNAG_00374	Senescence domain-containing protein	62 kDa	8.6106847	5.0989E-17	1.3649E-15
**J9VRI0**	CNAG_05678	Membrane protein	33 kDa	8.21320159	2.5965E-10	2.1792E-09
**J9VDW3**	CNAG_00091	Period circadian protein	53 kDa	8.1673787	1.3859E-20	6.5264E-19
**J9W049**	CNAG_06109	Cell surface protein	42 kDa	8.15127423	1.7092E-16	3.9513E-15
**J9VTQ3**	CNAG_01047	CND01770-like protein	14 kDa	7.87677157	6.0236E-18	1.9562E-16
**J9W025**	CNAG_06075	SBDS domain-containing protein	15 kDa	7.77333022	3.9009E-24	4.96E-22
**J9VPU6**	CNAG_03261	Zinc finger protein	32 kDa	7.7321878	1.1501E-11	1.2445E-10
**J9VK61**	CNAG_00848	Midasin	103 kDa	7.59563393	2.7958E-28	1.0157E-25
**J9VFV2**	CNAG_00827	Ribose 5-phosphate isomerase	18 kDa	7.58031136	2.0087E-05	6.5742E-05
**J9VE80**	CNAG_00485	Transmembrane protein	39 kDa	7.55283139	1.3329E-11	1.4302E-10
**J9VJA9**	CNAG_03991	Glycerophosphocholine acyltransferase 1	58 kDa	7.4299541	4.4171E-16	9.1323E-15
**J9VJQ8**	CNAG_01446	Heat shock protein 9/12-domain-containing protein	7 kDa	7.38584938	1.7035E-12	2.0531E-11
**J9VQK4**	CNAG_02118	PLAT domain-containing protein	41 kDa	7.33080713	1.5233E-17	4.6115E-16
**J9VNQ6**	CNAG_05783	Transcription initiation factor TFIIB	39 kDa	-18.805579	5.7549E-11	5.5857E-10
**J9VNE5**	CNAG_02564	tRNA-splicing endonuclease subunit Sen54	62 kDa	-17.884906	4.5159E-21	2.2085E-19
**J9VT84**	CNAG_06549	mRNA-capping enzyme subunit beta	44 kDa	-17.825342	5.61E-24	6.2027E-22
**J9VZG4**	CNAG_04409	Mucin	49 kDa	-17.697734	1.169E-18	4.0724E-17
**J9VGU4**	CNAG_03868	Hepatocellular carcinoma-associated antigen 59-domain-containing protein	34 kDa	-16.487606	2.4967E-27	5.772E-25
**J9VSV0**	CNAG_03240	alpha-1,2-Mannosidase	97 kDa	-16.464751	5.2557E-23	5.1405E-21
**J9VQ43**	CNAG_02306	Molybdopterin binding domain-containing protein	37 kDa	-15.911758	5.3278E-24	6.1584E-22
**J9VS54**	CNAG_05927	ATP synthase mitochondrial F1 complex assembly factor 1	43 kDa	-15.3327	2.919E-13	3.9908E-12
**J9VT10**	CNAG_01282	GRIP domain-containing protein	98 kDa	-15.325642	5.48E-23	5.1614E-21
**J9VL49**	CNAG_01181	Small subunit ribosomal protein S27Ae	18 kDa	-14.916612	4.6606E-33	5.926E-30
**J9VF33**	CNAG_00796	ABC multidrug transporter MDR1	152 kDa	-14.717844	1.4333E-20	6.6271E-19
**J9VZ71**	CNAG_04304	T-complex protein 1 subunit zeta	60 kDa	-14.525825	1.7509E-31	1.1131E-28
**J9VWD1**	CNAG_05360	ATP-dependent RNA helicase MAK5	85 kDa	-14.495982	7.6243E-17	1.9584E-15
**J9VX31**	CNAG_05585	Arf/Sar family protein	28 kDa	-14.228561	5.6124E-11	5.4684E-10
**J9VKC1**	CNAG_06741	ATP-dependent RNA helicase DBP10	88 kDa	-14.084074	4.3043E-17	1.1645E-15
*Heat Stress (37°C) *						
**J9VEK3**	CNAG_00639	OHCU_decarbox domain-containing protein	22 kDa	13.349515	8.6053E-15	1.4414E-13
**J9VKY5**	CNAG_01005	Glutaredoxin	16 kDa	10.2890332	1.0968E-17	2.9707E-16
**J9W182**	CNAG_06520	Methyltransf_25 domain-containing protein	26 kDa	9.33907912	8.9967E-19	3.3197E-17
**J9VHI6**	CNAG_00716	Cytochrome c	12 kDa	8.58129623	4.5879E-20	2.2039E-18
**J9VN56**	CNAG_03627	Peptidyl-prolyl cis-trans isomerase	17 kDa	8.485645	2.7664E-05	8.5999E-05
**J9VNY8**	CNAG_06663	Ubiquinol-cytochrome c reductase subunit 6	14 kDa	8.43669306	9.0341E-17	2.1699E-15
**J9VPH6**	CNAG_05839	Cytochrome c oxidase subunit	10 kDa	8.4130368	1.4871E-29	9.4654E-27
**J9VJP0**	CNAG_03049	Ubiquitin-like domain-containing protein	11 kDa	8.29049393	8.5495E-22	5.5813E-20
**J9VLE7**	CNAG_05011	Ferroxidase	21 kDa	8.19382097	1.7404E-24	2.11E-22
**J9VLJ9**	CNAG_01019	Superoxide dismutase [Cu-Zn] [SOD1]	16 kDa	7.90654249	8.7376E-23	6.7412E-21
**J9VQ81**	CNAG_02827	Ubiquitin-like protein Nedd8	8 kDa	7.71359385	4.0201E-11	3.5539E-10
**J9VJZ9**	CNAG_02926	Mitochondrial genome maintenance protein MGM101	30 kDa	7.65158141	1.0256E-10	8.4503E-10
**J9VWK2**	CNAG_04237	ABM domain-containing protein	12 kDa	7.58275692	2.7663E-21	1.5311E-19
**J9VTU2**	CNAG_05696	Ubiquitin-conjugating enzyme E2-16 kDa	16 kDa	7.56946674	3.549E-40	9.0357E-37
**J9VY76**	CNAG_06140	Long-chain fatty acid transporter	12 kDa	7.40490107	1.5156E-13	2.0309E-12
**J9VZG4**	CNAG_04409	Mucin	49 kDa	-19.402218	1.1434E-20	5.8221E-19
**J9VGU4**	CNAG_03868	Hepatocellular carcinoma-associated antigen 59-domain-containing protein	34 kDa	-16.917746	5.354E-28	1.7039E-25
**J9VNQ6**	CNAG_05783	Transcription initiation factor TFIIB	39 kDa	-16.364008	4.1858E-09	2.6249E-08
**J9VT84**	CNAG_06549	mRNA-capping enzyme subunit beta	44 kDa	-16.187868	1.0082E-21	6.0985E-20
**J9VSV0**	CNAG_03240	alpha-1,2-Mannosidase	97 kDa	-15.599226	9.7404E-22	6.0485E-20
**J9VZ71**	CNAG_04304	T-complex protein 1 subunit zeta	60 kDa	-15.186053	1.0489E-32	8.9018E-30
**J9VNE5**	CNAG_02564	tRNA-splicing endonuclease subunit Sen54	62 kDa	-15.04744	1.9332E-17	5.074E-16
**J9VKK4**	CNAG_01365	Reverse transcriptase domain-containing protein	85 kDa	-14.355011	1.5043E-24	1.9149E-22
**J9VGF7**	CNAG_03727	PH domain-containing protein	137 kDa	-14.150406	2.9605E-12	3.1406E-11
**J9VT10**	CNAG_01282	GRIP domain-containing protein	98 kDa	-14.104208	4.702E-21	2.5471E-19
**J9VF33**	CNAG_00796	ABC multidrug transporter MDR1	152 kDa	-14.089103	1.3267E-19	5.9261E-18
**J9VN33**	CNAG_02696	Exocyst protein	132 kDa	-13.942348	2.2294E-27	5.6759E-25
**J9VQH4**	CNAG_02762	F-type H-transporting ATPase subunit G	17 kDa	-13.892974	7.0624E-16	1.4984E-14
**J9VKK1**	CNAG_02745	AAA domain-containing protein	109 kDa	-13.73341	2.3298E-23	1.9772E-21
**J9VQ43**	CNAG_02306	Molybdopterin binding domain-containing protein	37 kDa	-12.649105	8.6533E-19	3.2399E-17

Network analysis via STRING [57] on upregulated and downregulated proteins during the stationary phase identified significant enrichment in metabolic pathways such as biosynthesis of secondary metabolites, pentose phosphate pathway, oxidative phosphorylation, fatty acid degradation, fructose and mannose metabolism, glycolysis/gluconeogenesis, methane metabolism, and citrate cycle (TCA cycle) among upregulated proteins. No significantly enriched KEGG pathways were found for downregulated proteins, though the septin ring and septin complex cellular components were notably downregulated (S1 Fig in S1 File).

### Global proteomic profiling: Changes to *C*. *neoformans* protein levels due to heat stress

**Fig 5:**
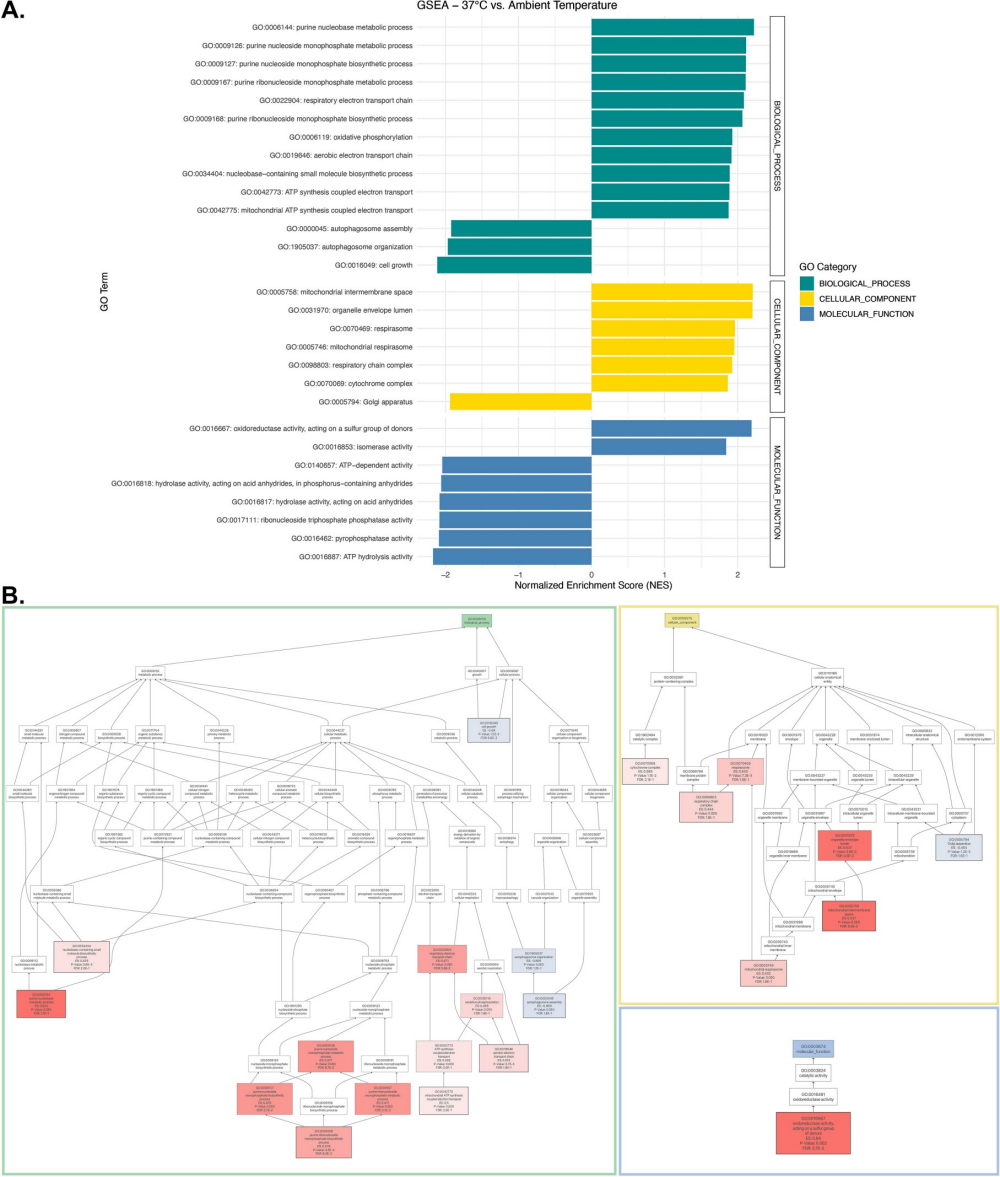
Gene set enrichment analysis (GSEA) of differentially expressed proteome at 37°C (heat stress) vs. ∼25°C (ambient temperature). (A) Bar plot illustrating significantly enriched gene ontology (GO) categories with p-value < 0.05 and FDR < 0.25 from GSEA. To account for the potential overlook of significant gene ontology terms, an FDR of 0.25 rather than 0.05 was used for GSEA. (B) Directed acyclic graph of enriched GO terms from GSEA, with positive enrichment are highlighted in red, while the GO terms with negative enrichment are highlighted in blue.

The differential expression analysis comparing the proteomes identified 396 proteins as significantly upregulated and 478 proteins as significantly downregulated after wild type exponential growth culture, initially grown at ∼25°C, was exposed to 37°C heat stress for two hours (Fig 3B) (Table 3). Gene Set Enrichment Analysis (GSEA) was conducted to identify the gene ontology terms significantly enriched during heat stress in *C*. *neoformans*. The GSEA revealed significant positive enrichment in the following biological processes: oxidative phosphorylation, mitochondrial ATP synthesis coupled transport, purine nucleobase metabolic process, and respiratory electron transport chain (Fig 5A and 5B). In contrast, biological processes such as autophagosome assembly/organization and cell growth exhibited significantly negative enrichment (Fig 5A and 5B). Additionally, cellular components like the mitochondria and respirasome demonstrated a positive enrichment score, whereas the Golgi apparatus was the only cellular compartment with a negative enrichment score. Among molecular functions, ATP-dependent activity, ATP hydrolysis, and pyrophosphatase activity were negatively enriched, while isomerase activity and oxidoreductase activity were positively enriched (Fig 5A and 5B).

A STRING network analysis was performed on the significantly upregulated and downregulated proteins during heat stress, independently. This analysis revealed that upregulated proteins were enriched in metabolic pathways including purine metabolism, carbon metabolism, glycolysis/gluconeogenesis, and biosynthesis of amino acids. Conversely, downregulated proteins were significantly enriched in the pathway of protein processing in the endoplasmic reticulum (S2 Fig in S1 File). Notably, the STRING analysis also suggested that septin complex is significantly downregulated at 37°C (S2 Fig in S1 File).

Comparing the heat stress and stationary growth phase (nutrient starvation) proteomes unveiled 162 overlapping upregulated proteins (S3A Fig in S1 File) and 255 overlapping downregulated proteins (S4A Fig in S1 File). A subsequent STRING network analysis on both sets of shared proteins revealed significant enrichment for RNA processing, ribosome biogenesis, cellular nitrogen compound metabolic process, mating projection, septin complex, and septin ring among the shared downregulated proteins (S4B Fig in S1 File). In contrast, shared upregulated proteins were significantly enriched for the biosynthesis of amino acids, citrate cycle (TCA), glycolysis/gluconeogenesis, and 2-Oxocarboxylic acid metabolism (S3B Fig in S1 File). Furthermore, the STRING-generated protein-protein interaction network for the shared upregulated proteins was significantly enriched (p-value: 0.000168), indicating a higher degree of interactions than expected from a random set of proteins. Collectively, these data outline the significantly upregulated and downregulated pathways during stress response in *C*. *neoformans*. Given the objective of this study to profile the protein interactome of Cdc3 and Cdc10, the significant enrichment of the septin complex & septin ring among the shared downregulated proteins warrants further attention.

### Septin interactome analysis: Identification of septin Cdc10 protein interactome via *co*-IP-MS

In this study, the interacting partners of septin proteins Cdc10 and Cdc3 were identified. Utilizing bait proteins with the mCherry fluorescent tag, immunoprecipitation was carried out using RFP-Trap agarose beads (Chromotek). The proteins extracted were then eluted and subjected to GeLC-MS/MS analysis, facilitating the identification of the bait protein (in this case, Cdc10 and Cdc3) and potential interacting partners via mass spectrometry. However, the co-IP-MS approach using a tagged protein as bait could potentially yield false positives. To mitigate this risk, an established practice is to employ a protein extract derived from a wild type strain that does not express the tagged bait protein, serving as a negative control [58, 59]. Only proteins exhibiting a logFC >2 and an adjusted p-value of <0.05 were considered sufficiently enriched when comparing the protein extract containing the tagged bait septin protein against the control. However, only those with a logFC >2 and an adjusted p-value of <0.001 were regarded as top candidates.

**Fig 6:**
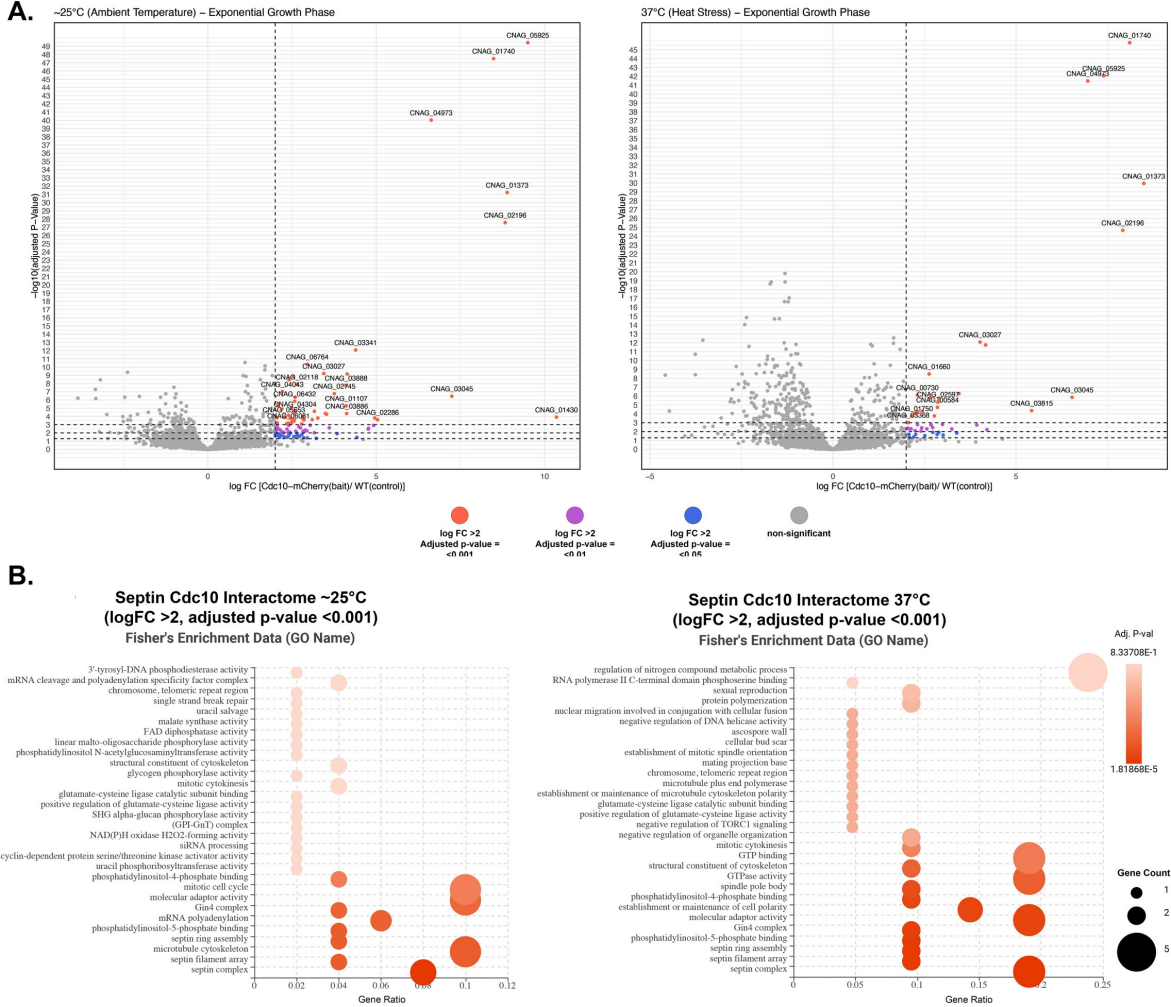
Characterization of Cdc10-mCherry interactome at ∼25°C and 37°C (heat stress). (A) Volcano plots of protein interactome results show proteins that are significantly enriched by Cdc10-mCherry co-immunoprecipitation from samples grown at ∼25 and 37°C, respectively. Significant interacting partners were determined by statistical t-test using logFC >2 and FDR of 0.05. The most significant candidates (highlighted) were considered those with a logFC >2 and FDR of 0.001. (B) Dot plots of functional enrichment analysis of septin Cdc10 protein interactome obtained from both temperature conditions using Fisher’s Exact Test. Gene count refers to the number of genes enriched in a GO term, and gene ratio is the percentage of total differential expressed genes in the given GO term.

At 25°C, 51 proteins were identified as significantly enriched in Cdc10-mCherry-expressing strain over the negative control (Table 4) (Fig 6A). Additionally, 22 proteins were deemed significantly enriched in Cdc10-mCherry-expressing strain over the negative control at 37°C (heat stress) (Table 5) (Fig 6A). However, significant enrichment of septin Cdc10 was unattainable in stationary growth phase samples. Although the protein Cdc10 was among the peptides identified in the pulldown (S3 Data), statistical analysis via a t-test did not validate Cdc10 as significantly enriched when compared to the negative control (S5 Fig in S1 File). In contrast, the entire septin complex was identified in the Cdc10 pulldown at both 25 and 37°C from exponentially grown cultures, which provided a strong validation for our approach (Fig 6A).

**Table 4:** List of significantly enriched (logFC >2 and adjusted p-value of <0.001) septin Cdc10 interacting partners based on samples incubated at ∼25°C (The significant binding partners list with logFC >2 and adjusted p-value of <0.05 can be found in S1 Table).

Uniprot ID	Gene ID	Protein Description	logFC	P.Value	adj.P.Val
**J9VUF2**	CNAG_05925	Septin ring protein CDC3	9.50067754	1.41E-53	3.60E-50
**J9VU34**	CNAG_01740	Septin ring protein CDC12	8.48429311	2.46E-51	3.14E-48
**J9VJ34**	CNAG_04973	Importin N-terminal domain-containing protein	6.63199126	1.11E-43	9.45E-41
**J9VMT9**	CNAG_01373	Septin ring protein CDC10	8.88837331	9.45E-35	6.04E-32
**J9VSM5**	CNAG_02196	Septin ring protein CDC11	8.83048158	5.33E-31	2.73E-28
**J9VT26**	CNAG_03341	DNA replication licensing factor MCM2	4.38989448	1.97E-15	8.39E-13
**J9VI22**	CNAG_06764	Short-chain dehydrogenase	2.95301731	1.43E-13	4.56E-11
**J9VPN4**	CNAG_03027	RRM domain-containing protein	3.44251215	2.84E-12	6.06E-10
**J9VWW6**	CNAG_05895	Protein CFT1	4.13907167	3.54E-12	6.97E-10
**J9VUN4**	CNAG_02434	Copper chaperone	2.54319131	8.35E-12	1.34E-09
**J9VRP5**	CNAG_05761	Cyclin-dependent kinases regulatory subunit	2.40652079	2.52E-11	3.39E-09
**J9VQK4**	CNAG_02118	PLAT domain-containing protein	2.64826061	1.02E-10	1.25E-08
**J9VGX5**	CNAG_03888	TPR_REGION domain-containing protein	4.10647724	1.52E-10	1.74E-08
**J9VH97**	CNAG_04043	CENP-V/GFA domain-containing protein	2.21129821	1.05E-09	9.60E-08
**J9VKK1**	CNAG_02745	AAA domain-containing protein	3.75469909	1.97E-09	1.68E-07
**J9VPM1**	CNAG_03045	Uncharacterized protein	7.2446064	4.57E-09	3.44E-07
**J9VHU3**	CNAG_02694	RICIN domain-containing protein	2.59133529	6.60E-09	4.53E-07
**J9W3A6**	CNAG_06432	Probable acetate kinase	2.57797242	2.82E-08	1.44E-06
**J9VZQ5**	CNAG_01970	CUE domain-containing protein	2.09134461	6.64E-08	3.15E-06
**J9VR06**	CNAG_01107	1-acyl-sn-glycerol-3-phosphate acyltransferase	4.1093202	1.09E-07	5.07E-06
**J9VKY5**	CNAG_01005	Glutaredoxin	2.08428301	1.18E-07	5.40E-06
**J9VH55**	CNAG_00575	Catalase	2.48285435	2.22E-07	9.46E-06
**J9VRE2**	CNAG_06666	Alpha-1,4 glucan phosphorylase	2.1999015	3.03E-07	1.25E-05
**J9VRK0**	CNAG_05703	Rho GTPase activator	3.16391853	6.23E-07	2.38E-05
**J9VZ71**	CNAG_04304	T-complex protein 1 subunit zeta	2.59200393	6.76E-07	2.54E-05
**J9VMV3**	CNAG_02793	Kynurenine 3-monooxygenase [BNA4]	2.56825057	8.80E-07	3.14E-05
**J9VQ43**	CNAG_02306	Molybdopterin binding domain-containing protein	3.48948846	1.25E-06	4.32E-05
**J9VJ17**	CNAG_03886	ELYS domain-containing protein	4.12305741	1.32E-06	4.44E-05
**J9VRR8**	CNAG_05787	Symplekin	3.52407636	1.67E-06	5.42E-05
**J9VTQ9**	CNAG_01627	Amino oxidase	2.39571357	2.35E-06	7.25E-05
**J9VKG1**	CNAG_01196	Tyrosyl-DNA phosphodiesterase 1	2.81532581	3.78E-06	0.00010468
**J9VJS2**	CNAG_01430	DASH complex subunit Duo1	10.3536759	4.55E-06	0.00012003
**J9W232**	CNAG_05653	Malate synthase	2.2573149	4.86E-06	0.00012562
**J9VKW5**	CNAG_00184	Pre-mRNA cleavage complex 2 protein Pcf11	2.29002765	4.83E-06	0.00012562
**J9VKK4**	CNAG_01365	Reverse transcriptase domain-containing protein	2.45260151	5.68E-06	0.00014249
**J9VUW0**	CNAG_02344	Uracil phosphoribosyl transferase	2.05210264	5.79E-06	0.0001428
**J9W0X0**	CNAG_06376	E3 ubiquitin-protein ligase PEP5	4.95824302	6.48E-06	0.00015337
**J9VWU3**	CNAG_04358	Iron-sulfur clusters transporter ATM1, mitochondrial	3.26533214	6.70E-06	0.00015649
**J9VE31**	CNAG_00429	tRNA-dihydrouridine synthase 1	2.8310883	9.71E-06	0.00021239
**J9VS16**	CNAG_07782	Oxidoreductase	3.09781418	1.17E-05	0.00024462
**J9VQ58**	CNAG_02286	Cytoplasmic protein	5.04092421	1.24E-05	0.00025495
**J9W1V7**	CNAG_01889	Glutathione S-transferase	2.56413623	1.31E-05	0.00026242
**J9VGG8**	CNAG_03738	Pantetheine-phosphate adenylyl transferase	2.85467071	1.31E-05	0.00026242
**J9VUT0**	CNAG_01807	ATP-dependent helicase	2.50181501	1.83E-05	0.0003314
**J9VP98**	CNAG_05758	Protein transport protein SFT2	2.55199886	2.24E-05	0.00039301
**J9VIM7**	CNAG_05042	Carnitine acetyltransferase	2.05558297	3.64E-05	0.00058902
**J9VQA8**	CNAG_01341	Mannose-6-phosphate isomerase	2.47996273	3.80E-05	0.00060353
**T2BNL9**	CNAG_01341	Mannose-6-phosphate isomerase, variant	2.47996273	3.80E-05	0.00060353
**J9W2F6**	CNAG_06081	Glucose oxidase	2.39430826	4.25E-05	0.00065153
**J9VJD3**	CNAG_00584	Profilin	2.3371529	5.23E-05	0.00078206
**J9VRI8**	CNAG_05688	V-type proton ATPase subunit F	2.78702894	5.34E-05	0.00079372

**Table 5:** List of significantly enriched (logFC >2 and adjusted p-value of <0.001) septin Cdc10 interacting partners based on samples incubated at 37°C (The significant binding partners list with logFC >2 and adjusted p-value of <0.05 can be found in S2 Table).

Uniprot ID	Gene ID	Protein Description	logFC	P.Value	adj.P.Val
**J9VU34**	CNAG_01740	Septin ring protein CDC12	8.10521185	6.25E-50	1.60E-46
**J9VUF2**	CNAG_05925	Septin ring protein CDC3	7.39355965	6.78E-46	8.66E-43
**J9VJ34**	CNAG_04973	Importin N-terminal domain-containing protein	6.96066216	3.91E-45	3.33E-42
**J9VMT9**	CNAG_01373	Septin ring protein CDC10	8.49115168	1.79E-33	1.14E-30
**J9VSM5**	CNAG_02196	Septin ring protein CDC11	7.91542203	4.27E-28	2.18E-25
**J9VPN4**	CNAG_03027	RRM domain-containing protein	4.01984211	6.49E-15	8.29E-13
**J9VT26**	CNAG_03341	DNA replication licensing factor MCM2	4.17076867	1.70E-14	1.74E-12
**J9W1B2**	CNAG_01660	eIF-2B GDP-GTP exchange factor subunit alpha	2.62777777	6.95E-11	3.23E-09
**J9VGX5**	CNAG_03888	TPR_REGION domain-containing protein	3.44169864	2.55E-08	5.30E-07
**J9VME1**	CNAG_00730	ABC multidrug transporter AFR1 [ATP-binding cassette transporter]	2.30543595	3.81E-08	7.60E-07
**J9VPM1**	CNAG_03045	Uncharacterized protein	6.53273489	7.08E-08	1.36E-06
**J9W340**	CNAG_06352	CAP-Gly domain-containing protein	2.64829063	8.14E-08	1.52E-06
**J9VTL1**	CNAG_01587	RNA polymerase-associated protein LEO1	2.88713011	9.70E-08	1.73E-06
**J9VQW4**	CNAG_02597	RNA-binding protein	2.87254561	2.66E-07	4.12E-06
**J9VJD3**	CNAG_00584	Profilin	2.85466575	1.42E-06	1.86E-05
**J9VIS6**	CNAG_03815	Uncharacterized protein	5.42624789	3.63E-06	4.36E-05
**J9VGH9**	CNAG_00292	Zinc finger protein	2.44362949	4.23E-06	5.00E-05
**J9VK83**	CNAG_01270	W2 domain-containing protein	2.30681513	4.83E-06	5.55E-05
**J9VTQ9**	CNAG_01627	Amino oxidase	2.24365788	8.24E-06	9.04E-05
**J9VQG8**	CNAG_03267	Splicing factor 3B subunit 2	2.77022713	1.73E-05	0.00017276
**J9VU43**	CNAG_01750	Hsp72-like protein	2.15098227	1.74E-05	0.00017317
**J9VQP8**	CNAG_03368	Microtubule Associated protein	2.05717738	0.00011753	0.00095935

Functional enrichment analysis of Cdc10’s interactome (candidates with logFC >2 and FDR <0.001) spotlighted septin filament array, septin assembly, and molecular adaptor activity as some of the GO terms with the highest gene ratio and gene count (Fig 6B). Other notable GO terms identified at ∼25°C for the Cdc10 interactome included structural constituent of the cytoskeleton, Gin4 complex, phosphatidylinositol-4,5-phosphate binding, and cyclin-dependent protein serine/threonine kinase activator activity (Fig 6B). Expectedly, functional enrichment of Cdc10’s interactome during heat stress exhibited common elements with the ambient temperature interactome such as septin ring assembly, septin filament array, Gin4 complex, phosphatidylinositol-4,5-phosphate binding, and molecular adaptor activity (Fig 6B). However, the analysis also uncovered GO terms specific to Cdc10’s interactome during heat stress: negative regulation of TORC1 signaling, negative regulation of organelle organization, positive regulation of glutamate-cysteine ligase activity, establishment or maintenance of microtubule cytoskeleton polarity, and regulation of nitrogen compound metabolic process (Fig 6B).

A comparison of Cdc10’s interactome between ambient temperature and heat stress (candidates with logFC >2 and FDR <0.001) identified 11 proteins common to both conditions (S6A Fig in S1 File). Further, the STRING protein-protein interaction (PPI) network analysis of the shared interacting proteins of Cdc10 during ambient temperature and heat stress displayed a high enrichment score (p-value: 0.00035), indicating more interactions than expected from a random protein set (S6B Fig in S1 File). Among the enriched gene ontology terms in the STRING network were septin ring organization, cytoskeleton-dependent cytokinesis, molecular adaptor activity, cell division site, and cell cortex.

### Septin interactome analysis: Identification of septin Cdc3 protein interactome via *co*-IP-MS

**Fig 7:**
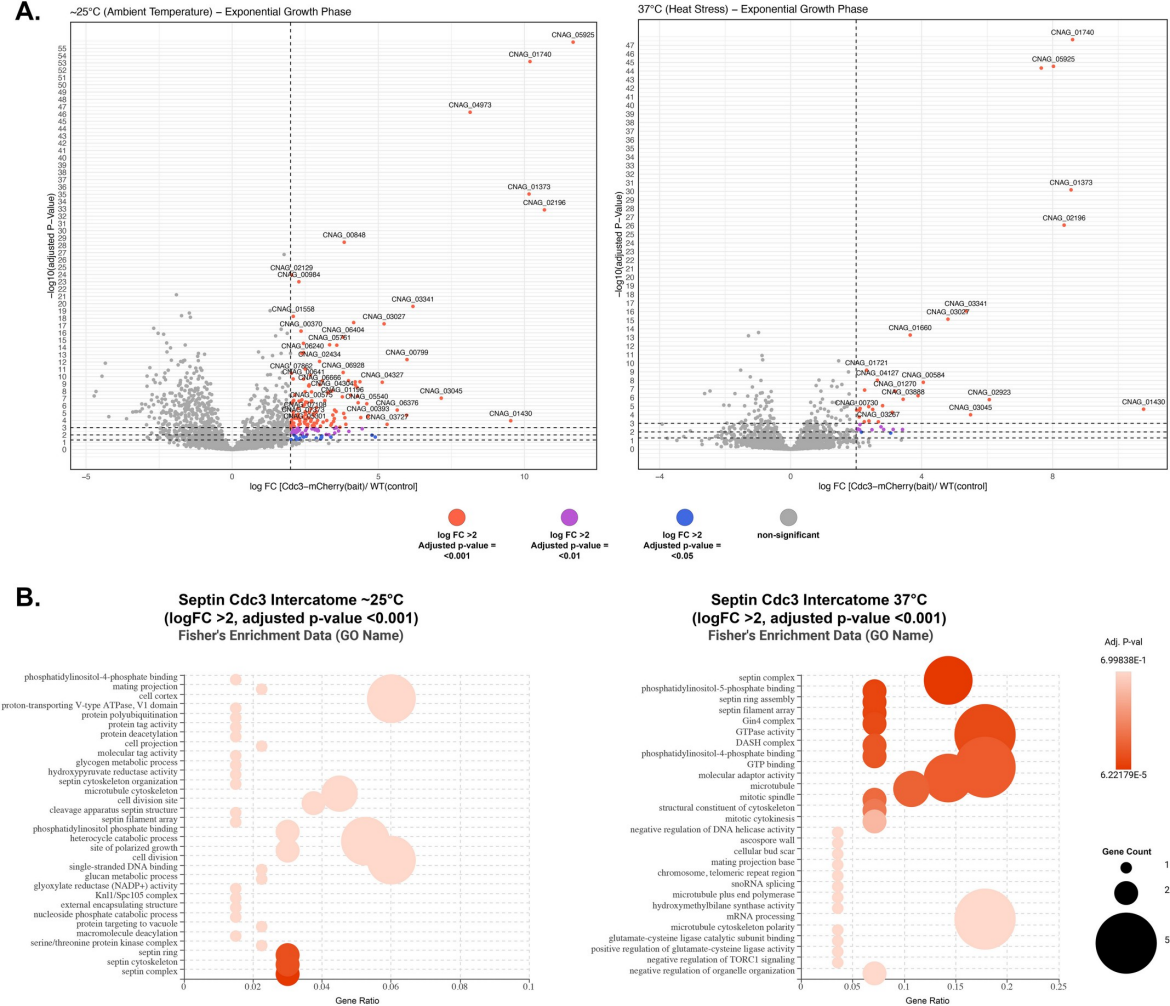
Characterization of Cdc3-mCherry interactome at ∼25°C and 37°C (heat stress). (A) Volcano plots of protein interactome results showing proteins that are significantly enriched by Cdc3-mCherry co-immunoprecipitation during ambient temperature and heat stress, respectively. Significant interacting partners were determined by statistical t-test using logFC >2 and FDR of 0.05. The most significant candidates (highlighted) were considered those with a logFC >2 and FDR of 0.001. (B) Dot plots of functional enrichment analysis of septin Cdc3 protein interactome during ambient temperature and heat stress, respectively, using Fisher’s Exact Test. Gene count refers to the number of genes enriched in a GO term, and gene ratio is the percentage of total differential expressed genes in the given GO term.

Mass spectrometry (MS) analysis identified 135 proteins significantly enriched in the Cdc3-mCherry pulldown from samples grown at ∼25°C (Table 6) (Fig 7A), and 29 significantly enriched proteins during heat stress (37°C) over the negative control (Table 7) (Fig 7A). Similar to the Cdc10 pulldown during stationary phase, the interactome of Cdc3 was not significantly identifiable in the stationary growth phase. The peptide corresponding to Cdc3 was identified in the Cdc3-mCherry co-immunoprecipitation (S3 Data); however, a statistical t-test analysis did not show significant enrichment of septin Cdc3 in the sample (S7 Fig in S1 File). Reassuringly, the entire septin complex was identified in the Cdc3-mCherry pulldown and subsequent MS analysis from exponentially grown cultures from both ∼25 and 37°C (Fig 7A) (Table 7).

**Table 6:** List of significantly enriched (logFC >2 and adjusted p-value of <0.001) septin Cdc3 interacting partners based on samples incubated at ∼25°C. (The significant binding partners list with logFC >2 and adjusted p-value of <0.05 can be found in S3 Table).

Uniprot ID	Gene ID	Protein Description	logFC	P.Value	adj.P.Val
**J9VUF2**	CNAG_05925	Septin ring protein CDC3	11.6649795	5.49E-60	1.40E-56
**J9VU34**	CNAG_01740	Septin ring protein CDC12	10.1903397	5.04E-57	6.45E-54
**J9VJ34**	CNAG_04973	Importin N-terminal domain-containing protein	8.14080986	6.76E-50	5.77E-47
**J9VMT9**	CNAG_01373	Septin ring protein CDC10	10.1573249	1.46E-38	9.36E-36
**J9VSM5**	CNAG_02196	Septin ring protein CDC11	10.6833569	2.71E-36	1.39E-33
**J9VK61**	CNAG_00848	Midasin	3.83365326	8.96E-32	3.82E-29
**J9VVE7**	CNAG_02129	ULP_PROTEASE domain-containing protein	2.03476974	3.70E-27	1.18E-24
**J9VLN0**	CNAG_00984	Glucose and ribitol dehydrogenase	2.28266662	3.52E-26	1.00E-23
**J9VT26**	CNAG_03341	DNA replication licensing factor MCM2	6.18520256	1.04E-22	2.42E-20
**J9VU15**	CNAG_01558	Chlorophyll synthesis pathway protein BchC	2.09000073	3.27E-21	5.59E-19
**J9VI22**	CNAG_06764	Short-chain dehydrogenase	4.156766	2.62E-20	3.94E-18
**J9VPN4**	CNAG_03027	RRM domain-containing protein	5.19795504	4.15E-20	5.90E-18
**J9VLE6**	CNAG_00370	Polyubiquitin	2.3563116	5.05E-19	5.88E-17
**J9VXH6**	CNAG_01920	Polyubiquitin	2.3563116	5.05E-19	5.88E-17
**J9VVZ2**	CNAG_06404	Metallothionein	3.79453963	4.19E-18	3.83E-16
**J9VS67**	CNAG_04903	Pollen-specific leucine-rich repeat extensin-like protein 1	2.43869922	3.32E-17	2.83E-15
**J9VRP5**	CNAG_05761	Cyclin-dependent kinases regulatory subunit	3.33372919	5.32E-17	4.39E-15
**J9VRG9**	CNAG_04127	Pre-mRNA-splicing factor CWC24	3.58005623	6.42E-17	4.98E-15
**J9VL36**	CNAG_06905	Cytoplasmic protein	2.44144667	7.39E-16	5.11E-14
**J9VW50**	CNAG_06240	Protein disulfide-isomerase	2.41144159	9.52E-16	6.41E-14
**O94746**	CNAG_03682	FK506-binding protein 1 [FRR1]	2.38164997	1.02E-15	6.57E-14
**J9VMN7**	CNAG_00799	Cellulase	5.98117538	8.59E-15	4.68E-13
**J9VUN4**	CNAG_02434	Copper chaperone	2.99024378	1.70E-14	8.37E-13
**J9VZ65**	CNAG_01721	Porphobilinogen deaminase	2.49870885	2.18E-13	9.15E-12
**J9VR41**	CNAG_06928	PH domain-containing protein	3.79781637	7.25E-13	2.77E-11
**J9VYC0**	CNAG_07862	Fumarate reductase	2.02433208	9.78E-13	3.52E-11
**J9VH97**	CNAG_04043	CENP-V/GFA domain-containing protein	2.67214408	1.90E-12	6.47E-11
**J9VN13**	CNAG_06576	Allergen	2.08751204	6.96E-12	2.12E-10
**J9VJH9**	CNAG_00641	Transcription elongation factor SPT5	2.44275635	7.40E-12	2.18E-10
**J9VLS0**	CNAG_00921	Glutathione transferase	3.98045482	1.30E-11	3.65E-10
**J9VIQ4**	CNAG_03789	Ribosome assembly factor mrt4	3.08349274	1.41E-11	3.88E-10
**J9VGX5**	CNAG_03888	TPR_REGION domain-containing protein	4.37201964	1.90E-11	5.17E-10
**J9VWI5**	CNAG_05409	Replication factor C subunit 2/4	4.20954168	1.97E-11	5.32E-10
**J9VS18**	CNAG_04327	Vigilin 1	5.13512451	2.26E-11	6.04E-10
**J9VRE2**	CNAG_06666	Alpha-1,4 glucan phosphorylase	2.99419828	5.49E-11	1.33E-09
**J9VKK1**	CNAG_02745	AAA domain-containing protein	4.20687175	5.64E-11	1.35E-09
**J9VIR0**	CNAG_07808	alanine—glyoxylate transaminase	2.63377368	5.82E-11	1.38E-09
**J9VYM9**	CNAG_01554	Sorting nexin-4	2.61688065	8.21E-11	1.87E-09
**J9VZQ5**	CNAG_01970	CUE domain-containing protein	2.63103227	9.06E-11	2.02E-09
**J9VUS1**	CNAG_01796	Uncharacterized protein	4.27175627	1.36E-10	2.93E-09
**J9VZ71**	CNAG_04304	T-complex protein 1 subunit zeta	3.42453735	4.55E-10	8.62E-09
**J9VQK4**	CNAG_02118	PLAT domain-containing protein	2.49455795	6.76E-10	1.23E-08
**J9VN96**	CNAG_01261	Myosin I binding protein	2.71488219	6.92E-10	1.26E-08
**J9VK83**	CNAG_01270	W2 domain-containing protein	3.30727209	7.15E-10	1.29E-08
**J9VTQ9**	CNAG_01627	Amino oxidase	3.27002019	1.04E-09	1.82E-08
**J9VPH6**	CNAG_05839	Cytochrome c oxidase subunit	2.14381566	2.08E-09	3.37E-08
**J9VE31**	CNAG_00429	tRNA-dihydrouridine synthase 1	4.05655868	2.24E-09	3.61E-08
**J9VIU7**	CNAG_03835	DASH complex subunit DAD3	4.28805373	3.24E-09	4.88E-08
**J9VKG1**	CNAG_01196	Tyrosyl-DNA phosphodiesterase 1	3.77024576	4.06E-09	6.01E-08
**J9VPM1**	CNAG_03045	Uncharacterized protein	7.15380646	6.51E-09	9.10E-08
**J9VJV8**	CNAG_01391	Hsp90 chaperone protein kinase-targeting subunit	2.38538484	1.15E-08	1.53E-07
**J9VZC5**	CNAG_05379	Regucalcin	2.10586013	1.48E-08	1.91E-07
**J9VWW6**	CNAG_05895	Protein CFT1	3.16860414	1.55E-08	1.98E-07
**J9VX79**	CNAG_01794	2-hydroxyacid dehydrogenase	2.7242679	1.69E-08	2.15E-07
**J9VKF2**	CNAG_02801	Thioredoxin	2.34076273	1.84E-08	2.31E-07
**J9VHF9**	CNAG_02850	Glucan endo-1,3-alpha-glucosidase agn1	2.28699817	1.89E-08	2.35E-07
**J9VJQ8**	CNAG_01446	Heat shock protein 9/12-domain-containing protein	2.08306393	2.04E-08	2.51E-07
**J9VH55**	CNAG_00575	Catalase	2.70902555	2.59E-08	3.06E-07
**J9VSQ9**	CNAG_02161	Inositol hexakisphosphate and diphosphoinositol-pentakisphosphate kinase	2.51398472	2.65E-08	3.11E-07
**J9W3A6**	CNAG_06432	Probable acetate kinase	2.57221693	2.99E-08	3.50E-07
**J9VR06**	CNAG_01107	1-acyl-sn-glycerol-3-phosphate acyltransferase	4.3098568	3.32E-08	3.83E-07
**J9VI77**	CNAG_03602	U3 small nucleolar RNA-associated protein 5	2.05534773	3.94E-08	4.44E-07
**O13465**	CNAG_05540	Urease [URE1]	4.60572348	4.83E-08	5.30E-07
**J9VPD5**	CNAG_02542	protein-ribulosamine 3-kinase	2.4721859	7.18E-08	7.46E-07
**J9VQ13**	CNAG_01439	U4/U6 small nuclear ribonucleoprotein PRP4	2.42318606	1.15E-07	1.14E-06
**J9VKK4**	CNAG_01365	Reverse transcriptase domain-containing protein	2.88298554	1.88E-07	1.80E-06
**J9VHU3**	CNAG_02694	RICIN domain-containing protein	2.24953941	2.41E-07	2.27E-06
**J9VMV3**	CNAG_02793	Kynurenine 3-monooxygenase [BNA4]	2.70031216	2.90E-07	2.63E-06
**J9W0X0**	CNAG_06376	E3 ubiquitin-protein ligase PEP5	5.64714992	4.55E-07	3.90E-06
**J9VIT7**	CNAG_05097	ATP-dependent (S)-NAD(P)H-hydrate dehydratase	3.50403019	4.59E-07	3.91E-06
**J9VS16**	CNAG_07782	Oxidoreductase	3.55727922	7.75E-07	6.30E-06
**J9VMJ0**	CNAG_02117	NAD-binding Rossmann fold oxidoreductase	2.87707775	8.07E-07	6.49E-06
**J9VQT6**	CNAG_07108	Tip120-family protein	2.49936787	8.14E-07	6.53E-06
**J9VS15**	CNAG_05886	Ubiquitin-conjugating enzyme E2	2.08939616	9.04E-07	7.16E-06
**J9VY38**	CNAG_04684	Protein transport protein SEC23	2.64165456	9.80E-07	7.58E-06
**J9VEV2**	CNAG_00466	Adaptin ear-binding coat-associated protein 2	2.50633604	1.26E-06	9.51E-06
**J9VSU3**	CNAG_02130	RNA-binding protein 39	3.81659604	1.53E-06	1.13E-05
**J9VWU8**	CNAG_05480	Phosphotransferase	2.78513479	1.70E-06	1.25E-05
**J9VL98**	CNAG_04955	Oxidoreductase	2.25831662	2.15E-06	1.53E-05
**J9VVN1**	CNAG_04771	Rab family protein	2.50832175	2.29E-06	1.62E-05
**J9VKE8**	CNAG_06710	ADF-H domain-containing protein	2.09497743	2.66E-06	1.85E-05
**J9VRR8**	CNAG_05787	Symplekin	3.44349186	2.67E-06	1.85E-05
**J9VZG4**	CNAG_04409	Mucin	5.96965635	2.89E-06	1.99E-05
**J9VJ63**	CNAG_00508	Vacuolar protein sorting-associated protein vps17	2.80206983	3.12E-06	2.13E-05
**J9VPN3**	CNAG_05307	Beta-catenin-like protein 1	2.1071495	3.29E-06	2.24E-05
**J9VGQ7**	CNAG_00393	1,4-alpha-glucan-branching enzyme	4.64695469	3.90E-06	2.61E-05
**J9W232**	CNAG_05653	Malate synthase	2.26563543	4.53E-06	3.01E-05
**J9VQH4**	CNAG_02762	F-type H-transporting ATPase subunit G	4.6412414	4.79E-06	3.15E-05
**J9VF80**	CNAG_07373	Carbamoyl-phosphate synthase, large subunit	2.42673454	5.23E-06	3.42E-05
**J9W095**	CNAG_04786	Histone deacetylation protein Rxt3	4.39931238	6.53E-06	4.20E-05
**T2BPP0**	CNAG_04786	Histone deacetylation protein Rxt3	4.39931238	6.53E-06	4.20E-05
**J9VX51**	CNAG_05410	B30.2/SPRY domain-containing protein	2.58284562	7.00E-06	4.42E-05
**J9VV27**	CNAG_07164	Scramblase-domain-containing protein	3.85676962	7.02E-06	4.42E-05
**J9VJD3**	CNAG_00584	Profilin	2.61962838	7.61E-06	4.74E-05
**J9VKW5**	CNAG_00184	Pre-mRNA cleavage complex 2 protein Pcf11	2.22202086	8.46E-06	5.23E-05
**J9VIY8**	CNAG_05122	Homoserine O-acetyltransferase	2.14121765	8.49E-06	5.23E-05
**J9VKQ6**	CNAG_01314	Vacuolar protein	3.49479807	9.36E-06	5.73E-05
**J9VVN4**	CNAG_06098	Glucosamine-6-phosphate isomerase	2.3028627	1.09E-05	6.56E-05
**J9VMK3**	CNAG_00760	Methylenetetrahydrofolate reductase	2.50695428	1.24E-05	7.31E-05
**J9VRK0**	CNAG_05703	Rho GTPase activator	2.70878731	1.32E-05	7.70E-05
**J9VQ08**	CNAG_01444	DNA-directed RNA polymerase II subunit RPB4	2.03682439	1.55E-05	8.97E-05
**J9VP42**	CNAG_05933	Sec1-like protein	2.92328571	1.75E-05	0.00010107
**J9W1V7**	CNAG_01889	Glutathione S-transferase	2.52017669	1.77E-05	0.00010212
**J9VGU4**	CNAG_03868	Hepatocellular carcinoma-associated antigen 59-domain-containing protein	3.23676532	1.82E-05	0.00010447
**J9VJS2**	CNAG_01430	DASH complex subunit Duo1	9.52978935	2.00E-05	0.00011351
**J9VJG5**	CNAG_05103	Transcription initiation factor TFIIF subunit beta	2.31300007	2.11E-05	0.00011955
**J9VQ05**	CNAG_02906	E2 ubiquitin-conjugating enzyme	3.46954612	2.50E-05	0.00014013
**J9VTE8**	CNAG_04690	Acylpyruvate hydrolase	2.68862442	2.53E-05	0.00014153
**J9VNZ4**	CNAG_06673	Maintenance of telomere capping protein 1	2.01889463	2.86E-05	0.00015733
**J9VDS2**	CNAG_00045	Proteasome inhibitor PI31 subunit	2.46930944	3.01E-05	0.00016519
**J9VTI8**	CNAG_04642	Tetraspanin Tsp2	3.11291721	3.32E-05	0.00018008
**J9VSC3**	CNAG_04443	DLH domain-containing protein	2.04855587	3.85E-05	0.00020294
**J9VLD8**	CNAG_05001	DUF953 domain-containing protein	2.88317841	4.40E-05	0.00022746
**J9VGD8**	CNAG_03708	Signal recognition particle subunit SRP72	2.10553122	4.55E-05	0.00023462
**J9VRI8**	CNAG_05688	V-type proton ATPase subunit F	2.80377513	4.86E-05	0.00024939
**J9VJD9**	CNAG_05301	Coronin	2.48752985	4.94E-05	0.00025307
**J9VGM4**	CNAG_00351	DBR1 domain-containing protein	3.40293994	5.53E-05	0.00028153
**J9VUT0**	CNAG_01807	ATP-dependent helicase	2.32795166	5.86E-05	0.0002956
**J9VJ19**	CNAG_04957	RRM Nup35-type domain-containing protein	3.88900454	7.00E-05	0.00034913
**J9VH39**	CNAG_00560	V-type H-transporting ATPase subunit E	2.00237785	7.07E-05	0.00035147
**J9VW66**	CNAG_06635	General negative regulator of transcription subunit	2.59039624	7.06E-05	0.00035147
**J9VGF7**	CNAG_03727	PH domain-containing protein	5.3000881	7.09E-05	0.00035162
**J9VWC9**	CNAG_04159	RBR-type E3 ubiquitin transferase	2.54971782	7.46E-05	0.00036641
**J9VN33**	CNAG_02696	Exocyst protein	2.28504247	7.89E-05	0.00038379
**J9VW37**	CNAG_04108	Protein-serine/threonine kinase	2.02452826	8.17E-05	0.0003947
**J9VQ88**	CNAG_03162	Vacuolar protein	3.37882551	9.98E-05	0.00047296
**J9VZC6**	CNAG_01802	Fe-S cluster assembly protein DRE2	2.74233699	0.00011523	0.00053808
**J9VUN1**	CNAG_04395	Protein BFR2	2.01511739	0.00012542	0.00057931
**J9VM44**	CNAG_00637	Cystathionine beta-synthase	2.80810355	0.00012657	0.00058359
**J9W086**	CNAG_06147	NADH-ubiquinone oxidoreductase 21.3 kDa subunit	3.0210744	0.00013671	0.00062583
**J9VZ01**	CNAG_04207	Protein CASP	2.12362848	0.00014886	0.00067423
**J9VRG3**	CNAG_06689	Cytoplasmic protein	2.58141518	0.00016245	0.00072549
**J9VPR8**	CNAG_02453	Metal ion binding	3.54771962	0.00019356	0.00084383
**J9VK75**	CNAG_02851	Threonine aldolase	3.68701469	0.00020182	0.00087832
**J9VUG6**	CNAG_04315	PHD-type domain-containing protein	2.00865782	0.00022804	0.0009824

**Table 7:** List of significantly enriched (logFC >2 and adjusted p-value of <0.001) septin Cdc3 interacting partners based on samples incubated at 37°C. (The significant binding partners list with logFC >2 and adjusted p-value of <0.05 can be found in S4 Table).

Uniprot ID	Gene ID	Protein Description	logFC	P.Value	adj.P.Val
**J9VU34**	CNAG_01740	Septin ring protein CDC12	8.60862215	8.75E-52	2.24E-48
**J9VUF2**	CNAG_05925	Septin ring protein CDC3	8.02385722	2.22E-48	2.84E-45
**J9VJ34**	CNAG_04973	Importin N-terminal domain-containing protein	7.65241598	5.27E-48	4.49E-45
**J9VMT9**	CNAG_01373	Septin ring protein CDC10	8.56174123	1.05E-33	6.73E-31
**J9VSM5**	CNAG_02196	Septin ring protein CDC11	8.34971345	1.68E-29	8.61E-27
**J9VT26**	CNAG_03341	DNA replication licensing factor MCM2	5.35319538	1.93E-19	8.24E-17
**J9VPN4**	CNAG_03027	RRM domain-containing protein	4.80360187	2.07E-18	7.56E-16
**J9W1B2**	CNAG_01660	eIF-2B GDP-GTP exchange factor subunit alpha	3.65011891	1.87E-16	5.31E-14
**J9VZ65**	CNAG_01721	Porphobilinogen deaminase	2.31950541	3.57E-12	6.42E-10
**J9VRG9**	CNAG_04127	Pre-mRNA-splicing factor CWC24	2.65023068	7.14E-11	9.61E-09
**J9VJD3**	CNAG_00584	Profilin	4.05055944	1.44E-10	1.67E-08
**J9VI22**	CNAG_06764	Short-chain dehydrogenase	2.25854258	1.45E-09	1.32E-07
**J9VK83**	CNAG_01270	W2 domain-containing protein	3.1864576	2.17E-09	1.79E-07
**J9VIU7**	CNAG_03835	DASH complex subunit DAD3	3.89086667	9.23E-09	6.05E-07
**J9VGX5**	CNAG_03888	TPR_REGION domain-containing protein	3.43503934	2.68E-08	1.56E-06
**J9VMD7**	CNAG_02923	Large subunit ribosomal protein L14	6.06550528	3.06E-08	1.74E-06
**J9VVN1**	CNAG_04771	Rab family protein	2.80759066	2.00E-07	8.81E-06
**J9W491**	CNAG_05629	Endoribonuclease YSH1	2.38493006	2.82E-07	1.13E-05
**J9VJ33**	CNAG_07361	Thioesterase superfamily protein	2.12198528	4.72E-07	1.83E-05
**J9VJS2**	CNAG_01430	DASH complex subunit Duo1	10.7765749	6.14E-07	2.25E-05
**J9VTL1**	CNAG_01587	RNA polymerase-associated protein LEO1	2.50553463	6.63E-07	2.39E-05
**J9VLP6**	CNAG_05101	U3 small nucleolar RNA-associated protein 12	2.10441257	8.19E-07	2.83E-05
**J9VME1**	CNAG_00730	ABC multidrug transporter AFR1 [ATP-binding cassette transporter]	2.01694213	8.77E-07	2.91E-05
**J9VE31**	CNAG_00429	tRNA-dihydrouridine synthase 1	3.102257	1.67E-06	5.28E-05
**J9VPM1**	CNAG_03045	Uncharacterized protein	5.49322323	3.27E-06	9.94E-05
**J9VTF7**	CNAG_01150	Omega-6 fatty acid desaturase (Delta-12 desaturase)	2.09273004	6.27E-06	0.00017817
**J9VV13**	CNAG_02282	Carboxypeptidase A4	2.39139336	2.22E-05	0.00052583
**J9VQP8**	CNAG_03368	Microtubule Associated protein	2.24947245	3.00E-05	0.00065071
**J9VQG8**	CNAG_03267	Splicing factor 3B subunit 2	2.67816834	3.05E-05	0.00065362

Functional enrichment of Cdc3’s interactome (candidates with logFC >2 and FDR <0.001) at ∼25°C revealed the following GO terms with the highest gene ratio and gene count: septin complex, septin cytoskeleton, cell cortex, microtubule cytoskeleton, and cell division (Fig 7B). Additionally, GO terms such as phosphatidylinositol phosphate binding, heterocycle catabolic process, and proton-transporting V-type ATPase, V1 domain, were also identified for Cdc3’s interactome at ∼25°C (Fig 7B).

During heat stress (37°C), functional enrichment for Cdc3’s proteome (candidates with logFC >2 and FDR <0.001) uncovered GO terms with the highest gene count and gene ratio including septin complex, Gin4 complex, molecular adaptor activity, GTP binding, GTPase activity, DASH complex, microtubule, and phosphatidylinositol-5-phosphate binding (Fig 7B). Noteworthy among the identified GO terms for Cdc3’s proteome during heat stress were the following: negative regulation of TORC1 signaling, negative regulation of organelle organization, positive regulation of glutamate-cysteine ligase activity, and mRNA processing (Fig 7B).

Comparison of Cdc3’s interactome (candidates with logFC >2 and FDR <0.001) from samples incubated at ∼25°C and those from 37°C identified 18 proteins common to both (S8A Fig in S1 File). These overlapping proteins reassuringly included the remaining 3 septins. A STRING PPI network analysis was conducted on these eighteen candidates, revealing gene ontology enrichments in the categories of cytoskeleton-dependent cytokinesis, mitotic cytokinesis, cell division, GTPase activity, septin complex, cell cortex, and microtubule cytoskeleton. Moreover, the interaction network showcased a significant enrichment (p-value: 0.00459), indicating a higher interaction level within the network than what would be expected from a random protein set (S8B Fig in S1 File).

### Characterizing the septin-complex interactome in *C*. *neoformans*: Comparison of Cdc3’s and Cdc10’s binding partners

To elucidate the common proteins between the Cdc10 and Cdc3 septin proteins interactome, a comparative analysis was performed on the significantly enriched proteins for both Cdc10 and Cdc3 at ambient temperature (∼25°C) and heat stress conditions (37°C), with a log2 fold change (logFC) greater than 2 and an adjusted p-value less than 0.05. The adjusted p-value cutoff was relaxed from 0.001 to 0.05 to ensure potential significant candidates were not overlooked. At ambient temperature (∼25°C), 112 proteins were identified as significantly enriched for septin Cdc10 (S1 Table), while 46 proteins were significantly enriched during heat stress (37°C) (S2 Table). The gene set functional enrichment analysis on the STRING PPI network for the 112 identified candidate binding partners for Cdc10 at ambient temperature highlighted several significantly enriched cellular components: septin complex, septin ring, cytoplasm, and intracellular anatomical structure (S9 Fig in S1 File).

Although the list of significantly enriched candidate binding partners for Cdc10 during heat stress is smaller in quantity than at ambient temperature (S1 and S2 Tables), the STRING PPI network’s gene set functional enrichment analysis for the 46 proteins that interact with Cdc10 during heat stress revealed a broader range of identified gene ontology terms (S10 Fig in S1 File). The biological processes significantly enriched for Cdc10’s interactome during heat stress include cytoskeleton-dependent cytokinesis and mitotic cytokinesis, with microtubule plus-end binding being the enriched molecular function. The enriched cellular components were septin complex, septin ring, spindle, microtubule cytoskeleton, cell cortex, and intracellular anatomical structure (S10 Fig in S1 File).

For septin Cdc3’s interactome (logFC >2 and adjusted p-value of <0.05), 196 proteins were identified as significantly enriched at ambient temperature (S3 Table), and 40 proteins during heat stress (S4 Table). The gene set functional enrichment analysis for the 196 proteins associated with Cdc3 at ambient temperature revealed septin complex, cytoplasm, intracellular anatomical structure, and cellular anatomical entity as the enriched cellular compartments (S11 Fig in S1 File). Furthermore, enzyme and protein binding were among the molecular functions enriched for Cdc3’s interactome at ambient temperature (S11 Fig in S1 File).

Similar to Cdc10’s interactome at 37°C (heat stress), the number of candidate binding partners of Cdc3 was smaller for heat stress than ambient temperature (S3 and S4 Tables). However, the STRING PPI network’ gene set functional enrichment analysis for Cdc3’s heat stress interactome showcased a wider range of gene ontology terms. The enriched cellular components for Cdc3’s interactome at 37°C include septin complex, septin ring, microtubule cytoskeleton, cell cortex, and ribonucleoprotein complex (S12 Fig in S1 File). Additionally, mRNA processing, cytoskeleton-dependent cytokinesis, and mitotic cytokinesis were significantly enriched biological processes for Cdc3’s interactome during heat stress (S12 Fig in S1 File).

**Fig 8:**
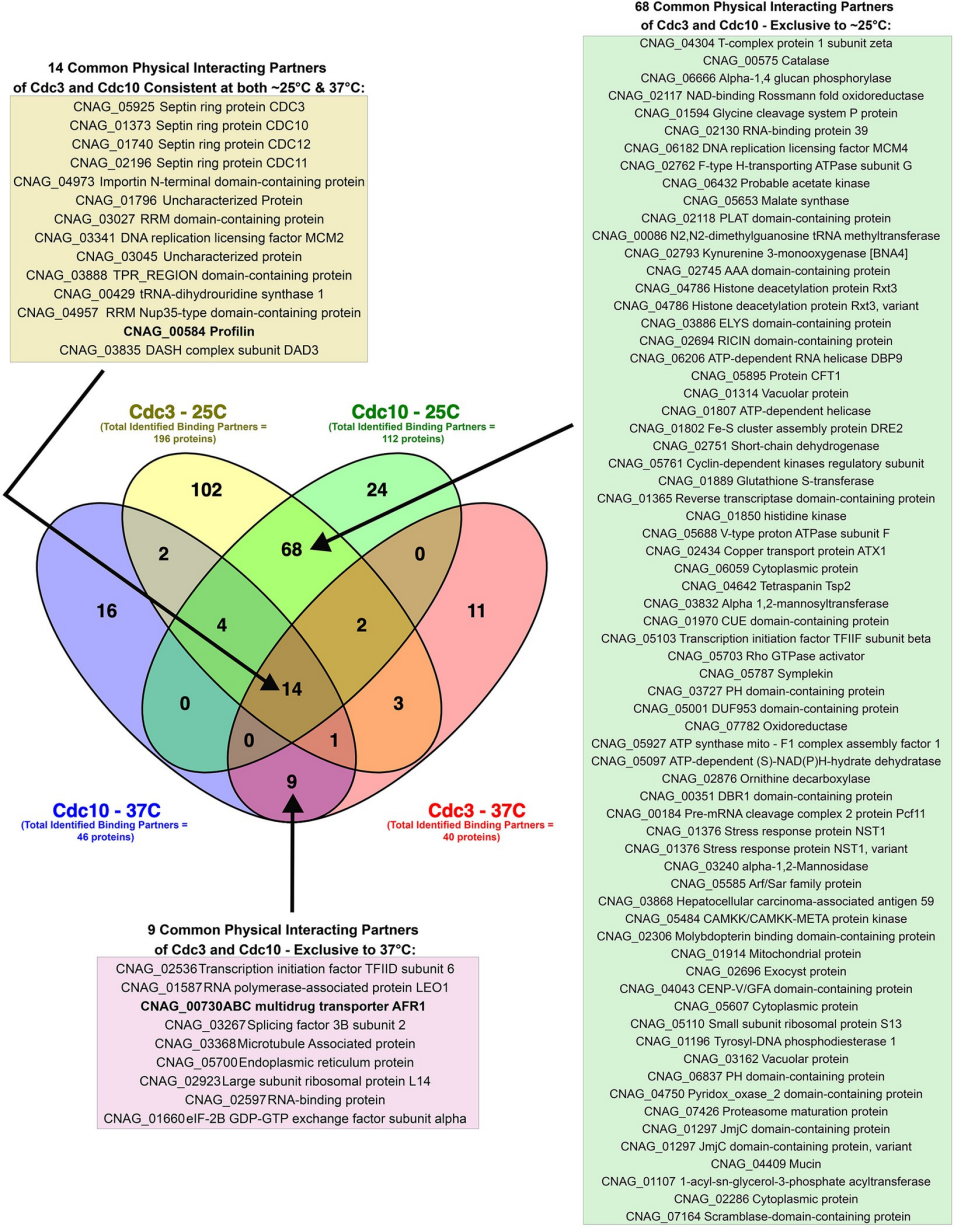
Overlap between Cdc10 and Cdc3 protein interactome. Significant interacting partners were determined by statistical t-test using logFC >2 and adjusted p-value of 0.05. Venn Diagram showing the overlap of significantly enriched proteins for co-immunoprecipitation of both Cdc3-mCherry and Cdc10-mCherry between ambient temperature (∼25°C) and heat stress (37°C).

The functional enrichment analyses of Cdc10’s and Cdc3’s independent interactomes during both ambient temperature and heat stress conditions offered further insights into the common pathways both septins might engage in. A total of 14 proteins were consistently identified for both septins at both temperature conditions (Fig 8).

**Fig 9:**
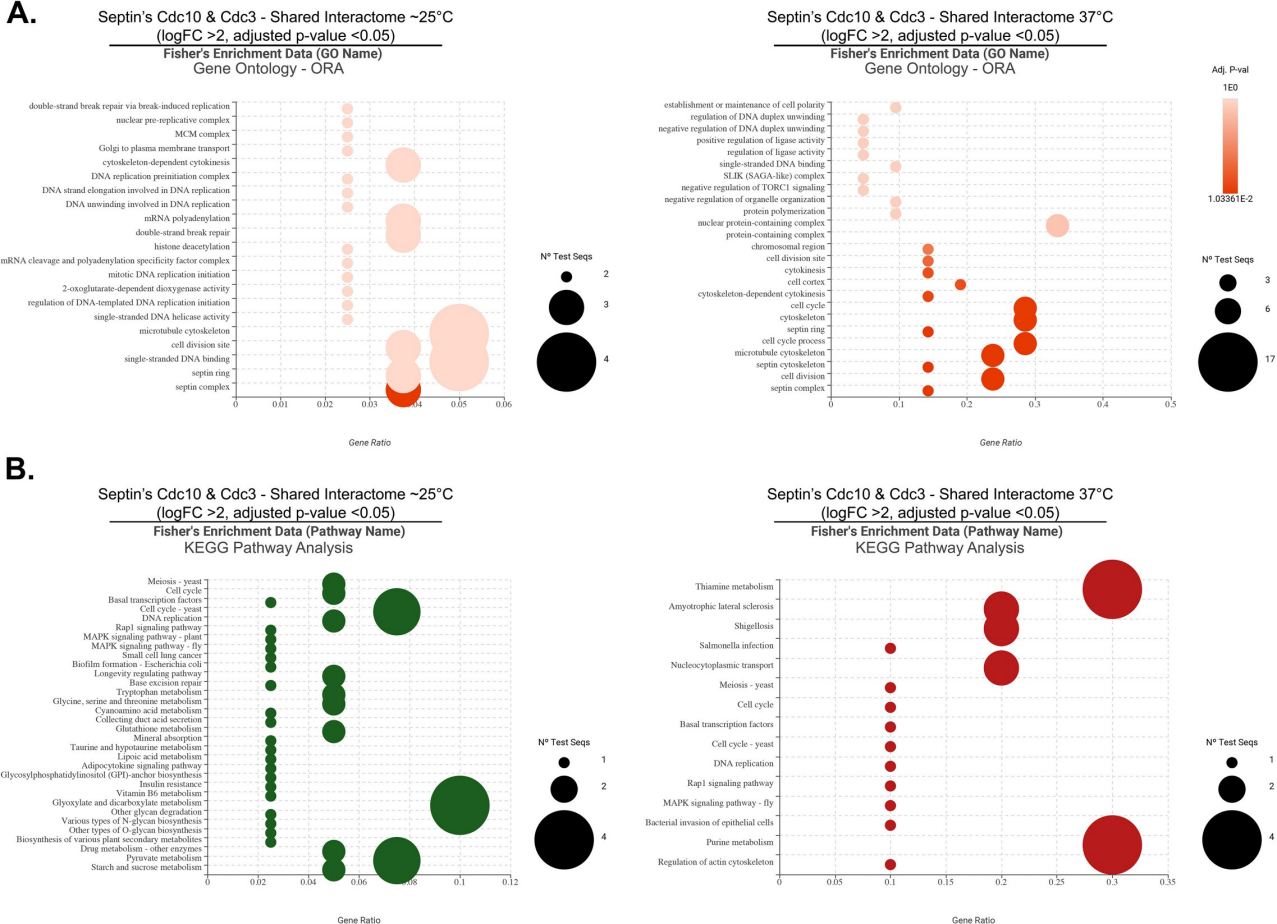
Septin Cdc3 and Cdc10 shared protein enrichment analysis. (A) Dot plots of functional enrichment analysis of septin Cdc10 and Cdc3 shared protein interactome during ambient temperature (∼25°C) and heat stress (37°C) using Fisher’s Exact Test. (B) Dot plots of KEGG pathway functional analysis of septin Cdc10 and Cdc3 shared protein interactome during ambient temperature and heat stress. Gene count refers to the number of genes enriched in a GO term, and gene ratio is the percentage of total differential expressed genes in the given GO term.

Furthermore, 68 proteins were identified as interacting with both septin Cdc3 and Cdc10 exclusively at 25°C (Fig 8), whereas 9 proteins were found to interact with Cdc10 and Cdc3 exclusively at 37°C (Fig 8). To delve into the interactions of both Cdc3 and Cdc10 in the context of the septin complex, a functional enrichment analysis employing Fisher’s exact test was conducted on all proteins identified as consistently interacting with both septins at 25°C. Hence, the 68 proteins (exclusive to ambient temperature), and the 14 proteins identified across all ambient temperature and heat stress conditions for both septins were regarded as part of the ambient temperature septin complex interactome (Fig 8). The inclusion of the set of 14 proteins in this analysis is justified as these peptides have indeed been identified during ambient temperature growth for both septin Cdc10 and Cdc3. Functional enrichment analysis utilizing Fisher’s exact test for the both Cdc3 and Cdc10 associated proteome at ambient temperature revealed the septin complex, septin ring, cell division site, microtubule cytoskeleton, cytoskeleton-dependent cytokinesis, mRNA polyadenylation, and double-strand break repair as the enriched GO terms for ambient temperature (Fig 9A). Furthermore, the following enriched KEGG pathways for the septin interactome at ambient temperature were the following: cell cycle, glyoxylate and dicarboxylate metabolism, pyruvate metabolism, longevity regulating pathway, base excision repair, and the MPAK signaling pathway (Fig 9B).

A total of 25 proteins were identified as part of the septin interactome at 37°C (Fig 8). Out of the 25 proteins, 14 are consistently expressed across both temperature conditions (as described above) and 9 are exclusively enriched at 37°C. Consequently, the 9 proteins (exclusive to 37°C), and the 14 proteins identified across all ambient temperature and heat stress conditions for both septins were regarded as part of the heat stress septin complex interactome (Fig 8). In addition, functional enrichment analysis utilizing Fisher’s exact test for both Cdc3 and Cdc10 associated proteome during heat stress revealed the following enriched GO terms: cell division, microtubule cytoskeleton, cell cycle, septin complex, septin ring, nuclear protein-containing complex, negative regulation of TORC1 signaling, and negative regulation of organelle organization (Fig 9A). Furthermore, the following KEGG pathway were among the significantly enriched in the septin Cdc3 and Cdc10 shared interactome during heat stress: regulation of actin cytoskeleton, nucleocytoplasmic transport, thiamine metabolism, cell cycle, purine metabolism, Rap1 signaling, and MAPK signaling pathway (Fig 9B).

The STRING PPI network for the significantly enriched binding partners of septins across all conditions displayed a significant enrichment value (S13B Fig in S1 File), indicating a higher level of interactions than expected from a random protein set. The two identified local STRING clusters for Cdc3 and Cdc10 during growth at ∼25°C and heat stress were the septin complex & cell cortex, and MAPK signaling pathway-yeast (S13B Fig in S1 File).

### Cdc10 associates with the actin-binding protein profilin

One of the most significant binding partners identified in this study is profilin (CNAG_00584). profilin was identified as associated with both Cdc3 and Cdc10 at both ∼25 and 37°C (Figs 6 and 7) (Tables 4–7). Profilin binds to actin, a key component of the cytoskeleton, and regulates its polymerization and depolymerization. In higher eukaryotes, this allows cells to undergo shape changes and move in response to external signals [60].

**Fig 10:**
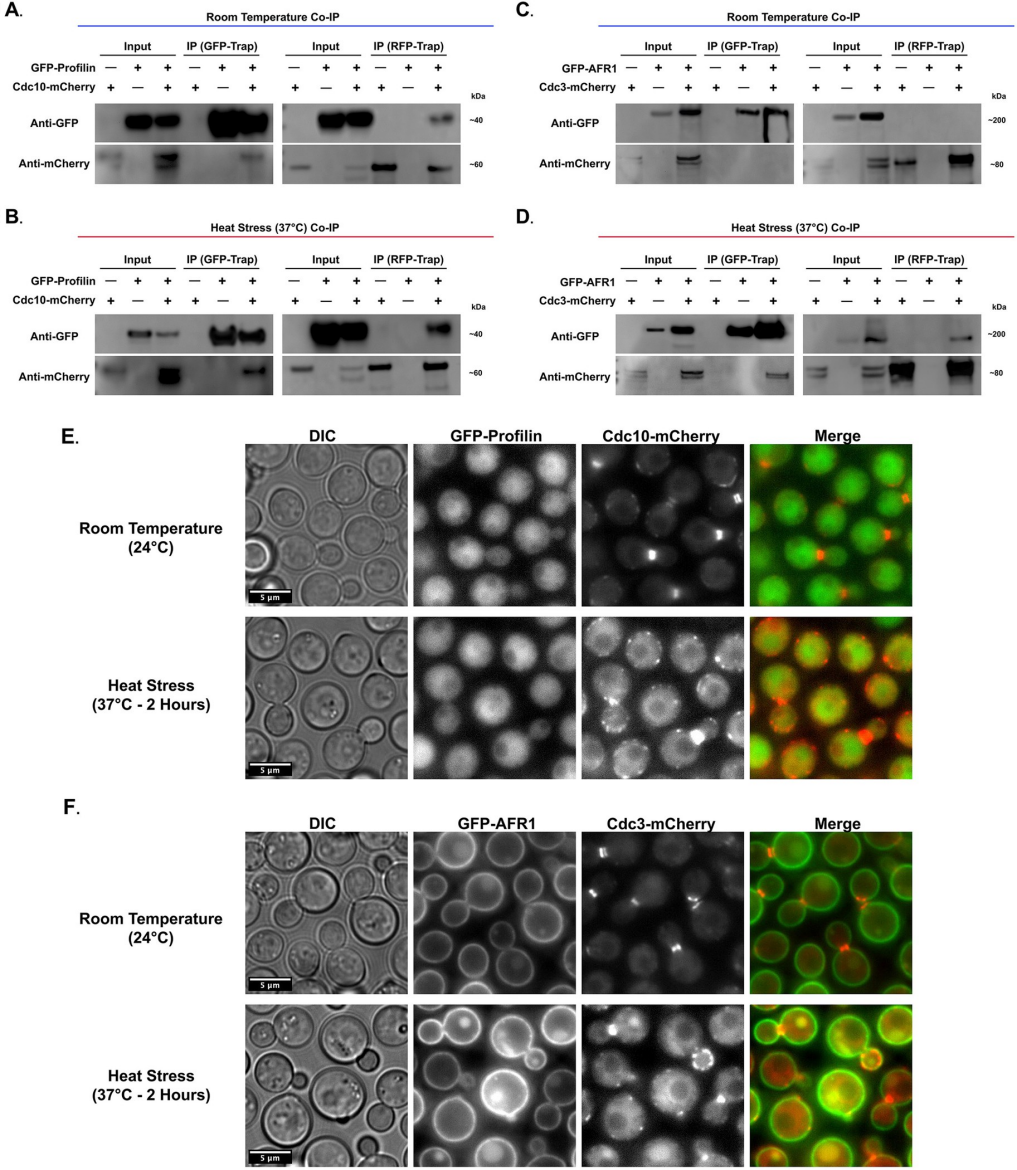
Co-immunoprecipitation and fluorescence microscopy of septins Cdc3 and Cdc10 and their interacting partners profilin and AFR1. A co-IP of the Cdc10-mCherry using the GFP-Trap resin and a co-IP of the GFP-profilin using the RFP-Trap resin, at (A) 25°C and (B) heat stress (37°C). A co-IP of the Cdc3-mCherry using the GFP-Trap resin and a co-IP of the GFP-AFR1 using the RFP-Trap resin, at (C) 25°C and (D) heat stress (37°C). Strain that expresses only Cdc10-mCherry or Cdc3-mcherry, and strains that express only GFP-tagged interacting proteins were used as negative controls. The membranes were initially probed with an anti-GFP antibody and imaged and subsequently membranes were stripped and probed with an anti-RFP antibody to detect the precipitated GFP-tagged and mCherry-tagged proteins, respectively. (E) Cells expressing GFP-profilin and Cdc10-mCherry were visualized via live fluorescent microscopy from a culture grown at ∼25°C and after 2 hours of incubation at 37°C. GFP-profilin doesn’t show co-localization with septin Cdc10-mCherry. (F) Cells expressing GFP-Afr1 and Cdc3-mCherry were visualized via live fluorescent microscopy from a culture grown at ∼25°C and after 2 hours of incubation at 37°C. GFP-AFR1 does not colocalize with Cdc3-mCherry at 25°C; however, it co-localizes partially during high temperature stress.

To validate the mass spectrometry data, and confirm the association of Cdc10 with profilin, GFP-profilin was co-expressed with Cdc10-mCherry in *C*. *neoformans*, and the Cdc10-mCherry was precipitated with RFP-Trap resin. GFP-profilin was efficiently co-precipitated from cells that also expressed Cdc10-mCherry; importantly, the GFP-profilin did not show affinity to the RFP-Trap resin (Fig 10A and 10B). In addition, GFP-profilin was precipitated with GFP-Trap resin from cells expressing Cdc10-mCherry, and Cdc10-mCherry was successfully co-precipitated (Fig 10B).

Live fluorescent microscopy revealed that at ∼25°C, Cdc10-mCherry localized to the mother-bud neck, as expected, whereas GFP-profilin fluorescence was mostly cytoplasmic (Fig 10E). At 37°C, while GFP-profilin remained cytoplasmic, the Cdc10-mCherry formed punctate localization (in addition to its localization at the mother-bud neck) that likely corresponded to the plasma membrane (Fig 10E). Although there was no evident co-localization observed for GFP-profilin and Cdc10-mCherry, the association of Cdc10 with profilin detected via co-immunoprecipitation was independent of growth temperature, which is in accord with the mass spectrometry data (Fig 10A, 10B and 10E). It is also worth noting that profilin (CNAG_00584) was among the proteins that were identified as significantly upregulated with a LogFC of 3.446 during heat stress when compared to ∼25°C (S3 Data). Consequently, we hypothesize that septins possibly contribute to heat stress response via their association with profilin and the actin-cytoskeleton. Furthermore, the microtubule cytoskeleton was one of the enriched gene ontology terms in the functional enrichment conducted on the shared interactome of Cdc3 and Cdc10 both at ambient temperature (∼25°C), and during heat stress (37°C) (Figs 6 and 7).

### Cdc3 associates with the ATP-binding cassette transporter Afr1, and partially colocalizes with Afr1 during heat stress

The mass spectrometry data revealed both septin proteins Cdc3 and Cdc10 associate with Afr1 (CNAG_00730) exclusively during heat stress (37°C) (Figs 6 and 7) (Tables 4–7). *AFR1*, which stands for Antifungal Resistance 1, encodes an ATP-binding cassette (ABC) transporter protein that is likely associated with the plasma membrane in *C*. *neoformans* [61, 62].

To validate the mass spectrometry data and confirm the association of Cdc3 with Afr1, GFP-Afr1 was co-expressed with Cdc3-mCherry, and the Cdc3-mCherry was precipitated with RFP-Trap resin. GFP-Afr1 was successfully co-precipitated from cells that also expressed Cdc3-mCherry when cells were incubated at 37°C but not from cultures incubated at ∼25°C. In addition, the GFP chimera did not show affinity to the RFP-Trap resin (Fig 10C and 10D). Reassuringly, GFP-Afr1 was precipitated from cells expressing Cdc3-mCherry with GFP-Trap resin, and Cdc3-mCherry was successfully co-precipitated when cells were grown at 37°C but no association was detected when cells were grown at ∼25°C. (Fig 10C and 10D).

Live fluorescent microscopy revealed that at ∼25°C, Cdc3-mCherry localized to the mother-bud neck, as expected, whereas GFP-Afr1 fluorescence was mostly visible as a rim of the cells likely corresponding to the predicted plasma membrane localization (Fig 10F). At ∼25°C, there was no evident co-localization observed for GFP-Afr1 and Cdc3-mCherry. At 37°C, while GFP-Afr1 remained localized to the rim of the cell, likely corresponding to the plasma membrane, the Cdc3-mCherry formed punctate localization that partially overlapped with localization of GFP-Afr1 (Fig 10F). Thus, the partial co-localization between GFP-Afr1 and Cdc3-mCherry detected at 37°C is consistent with the association of Cdc3-mCherry with GFP-Afr1 that was detected both via co-immunoprecipitation and mass spectrometry exclusively at 37°C (Fig 10C, 10D and 10F).

## Discussion

The analysis of the proteomic landscapes of *C*. *neoformans* strain H99 under two stress conditions, nutrient deprivation and temperature shift from ∼25 to 37°C, has provided insight into the molecular mechanisms of adaptation to the host environment. Our proteomic profiling underscored numerous upregulated and downregulated processes that collectively drive both the stationary phase growth, and the heat stress response of *C*. *neoformans*. Among the upregulated processes, elements involved in heat shock response, antioxidative defense, and protein refolding were identified, depicting a concerted effort to maintain cellular integrity under stress conditions. In contrast, processes related to cellular replication, certain metabolic pathways, and protein synthesis were downregulated, potentially revealing a resource conservation strategy employed by the fungus under environmental stress. Remarkably, all four septin proteins were downregulated during heat stress and stationary phase growth in *C*. *neoformans*. Additional support for this finding comes from our investigation of *C*. *neoformans* transcriptome upon 37°C shift, where the expression levels of all septin-encoding genes were significantly lower as compared to ∼25°C (Stempinski P, Kozubowski L, unpublished data). These findings appear to contradict the essential role for septins Cdc3 and Cdc12 in proliferation at 37°C and at conditions that trigger/interrupt stress response, including interruption of plasma membrane, cell wall integrity or inhibition of stress-related phosphatase, calcineurin [8, 63, 64]. However, the importance of a gene in stress response is seldom reflected by high expression during stress [65]. Thus, the interplay between septin proteins and their interactors may represent a coherent adaptive strategy to rewire cellular circuitry in favor of survival under hostile conditions.

Additional insights into our findings are provided by a comparative analysis with a recent study by Stempinski, Zielinski et al., which explored *C*. *neoformans* gene deletion strains exhibiting growth defects at high temperatures [64]. Our proteomic profiling data at 37°C (heat stress) overlapped with eight downregulated proteins and four upregulated proteins delineated in the aforementioned study. Notably, the downregulation of proteins such as pyruvate dehydrogenase kinase (CNAG_04108), Cdc11 (CNAG_02196), Cdc12 (CNAG_01740), and cytoskeletal regulatory protein-binding protein (CNAG_01918) in our data hints at a complex interplay of metabolic and cytoskeletal regulatory pathways under thermal stress. Meanwhile, the upregulation of proteins like endoplasmic reticulum protein (CNAG_02891), CMGC/MAPK protein kinase (CNAG_04514), and mitochondrial manganese superoxide dismutase (CNAG_04388) underscore the organism’s endeavor to uphold cellular homeostasis and combat oxidative stress during heat stress. These data not only corroborate the findings from the previous study but also broaden the scope of our understanding regarding the heat stress response in *C*. *neoformans*.

### Uncovering septin interactions in *C*. *neoformans*

The elution fractions based on the strain H99 provided an avenue to control for potential false positives in the analysis of septin-binding partners; the proteins that bind to the ChromoTek® agarose beads RFP-TRAP (affinity resin coupled with RFP Nanobody/VHH) in these control pulldowns were considered contaminants. Thus, a comparison of the peptides detected in the elution fraction of the strain H99 and the elution fraction that was based on the strains expressing mCherry tagged septin proteins provided a comprehensive list of proteins that represented candidate binding partners of septins. Arguably, the more stringent control could have been a strain that expresses the mCherry protein, which would have accounted for those proteins that have affinity to this fluorescent tag. In that regard, proteins identified as binding partners of only one of the septins (either Cdc3-mCherry or Cdc10-mCherry) may be considered most likely physiologically relevant. Nonetheless, the inclusion of 5 biological replicates and the correction of the results by the relative abundance of the candidate proteins in the cell lysate (the enrichment in the pulldown versus abundance in the protein lysate) provided a robust list of *bona fide* septin interactors with high confidence level.

Our investigation into septins protein interactome elucidates their fundamental role as scaffolding entities, orchestrating a plethora of cellular processes essential for maintaining cellular homeostasis. These processes include cell division, membrane trafficking, and cellular signaling [12]. A notable validation of our findings was attained through the successful elution of the entire septin complex during both Cdc3-mCherry and Cdc10-mCherry pulldown assays at both ambient temperature (∼25°C) and heat stress (37°) conditions. However, we were not able to effectively pull down the bait proteins from the stationary phase cultures, suggesting a phase-specific modulation of septin complex architecture and dynamics. This deviation underscores the necessity for further examination into the spatiotemporal dynamics governing septin assembly and function across various environmental conditions.

### Association of septins with profilin

Profilin was a significant and confirmed interacting partner for the shared interactome of Cdc3 and Cdc10 during both ambient temperature (∼25°C) and heat stress (37°C). The role of profilin during thermal stress is well documented. Polymerized actin (F-actin) is inherently unstable at high temperatures, and profilin helps maintain actin in its filamentous state during heat stress, possibly by stimulating actin polymerization in a proper spatiotemporal manner [66].

The association of septin Cdc3 and Cdc10 with profilin during both ambient temperature (∼25°C) and heat stress (37°C) conditions implicates the septin complex might have a role in heat stress response in *C*. *neoformans* by contributing to F-actin stability. This is further supported by our finding that profilin was among the proteins identified as significantly upregulated, with a LogFC of 3.446, at 37°C (S3 Data). Moreover, Cdc3 and Cdc10’s association with profilin might link the septin complex to more than one stress response pathway, as discussed below.

Profilin helps protect cells from thermal damage by stabilizing the cytoskeleton and preventing protein denaturation. In *S*. *cerevisiae*, profilin plays a significant role in maintaining normal levels of F-actin, especially in response to shifts to high temperature [66]. However, profilin is not essential for maintaining F-actin concentrations during steady-state growth at room temperature [66]. These findings may provide an explanation as to why in *C*. *neoformans* Cdc3 and Cdc12 are only essential during stress, including growth at 37°C.

In addition to actin, profilin interacts with other proteins, such as integrins and growth factors, to coordinate cell signaling and behavior [67]. Profilin also binds to membrane phosphoinositides such as P­I(3,4,5)P­3 and P­I(4,5)P­2, which inhibits profilin’s interactions with G-actin and proline-rich stretches [68–72]. Association of profilin with membrane phosphoinositides has been proposed to regulate the temporal and spatial levels of profilin-actin by two possible mechanisms [67, 73]. One possibility is that external signal-mediated phosphorylation of phospholipase C hydrolyzes P­I(4,5)P­2, releasing membrane-bound profilin to presumably facilitate actin assembly by the proteins formin and Ena/VASP. Second, sequestration of profilin to membrane regions with high concentrations of P­I(4,5)P­2 could increase the level of free G-actin, unbound to profilin, which might preferentially incorporate into branched actin filament networks generated by the Arp2/3 complex [73, 74].

Profilin has also been identified as having a role in stress granules in yeast and mammalian cells [75]. Stress granules are formed in response to stress conditions such as heat shock or oxidative stress [76–78]. Profilin 1 localizes to stress granules upon exposure to diverse environmental stressors. This links profilin 1, the newest amyotrophic lateral sclerosis (ALS) disease protein, to stress granules. The study by Figley, et al. also found that ALS-linked profilin 1 mutations alter stress granule dynamics, providing a potential mechanism to explain their role in disease [75]. Notably, in our study, one of the pathways with highest gene count and gene ratio that was identified in the KEGG functional enrichment for the shared proteome of Cdc3 and Cdc10 during heat stress (37°C) is ALS disease protein. Strikingly, a recent study in *C*. *neoformans* identified septin Cdc3 as one of the targets (direct or indirect) of stress related phosphatase calcineurin, in addition to other targets including proteins involved in RNA processing, stability, and translation, which colocalize together with calcineurin in stress granules/P-bodies upon thermal stress [79]. Moreover, *C*. *neoformans* strains lacking Cdc3 or Cdc12 are inviable in the absence of calcineurin activity at 25°C [64]. These findings and data presented here collectively suggest that septins Cdc3 and Cdc10 are involved actin dynamics and stress granule formation and/or dynamics during heat stress through their association with profilin.

Given the above findings, we predicted the GFP-profilin would form fluorescent puncta at the plasma membrane and/or at the stress granules/P-bodies when *C*. *neoformans* is shifted to 37°C. Interestingly, we observed Cdc3-mCherry and Cdc10-mCherry forming puncta at the plasma membrane and in the cytoplasm (which may correspond to stress granules) specifically at 37°C. However, GFP-profilin exhibited an overall cytoplasmic signal at both ∼25 and 37°C. One explanation for this inconsistency is that the plasma membrane signal of GFP-profilin is not discernible due to the overexpression of the chimera, in contrast to the native level of expression of Cdc3-mCherry and Cdc10-mCherry. A non-exclusive possibility is that the GFP-profilin may not be fully functional while still retaining its capacity to associate with the septins.

### The connection between profilin, septins, and the MAPK pathway

The highly conserved Mitogen-Activated Protein Kinase (MAPK) pathway facilitates responses to changes in environmental conditions and various other stimuli; It is a three-tiered cascade of protein kinases [80]. The components of these pathways and the mechanisms by which they operate were first identified and characterized in *S*. *cerevisiae* and are now known to have been conserved during the evolution of the entire eukaryotic kingdom [81]. Furthermore, these pathways regulate downstream molecular and cellular processes and are controlled by regulatory factors both internal and external to the pathways [82, 83].

*S*. *cerevisiae’s* profilin deficient mutants exhibit various abnormalities such as sensitivity to caffeine and NaCl, growth defects at altered temperatures, accumulation of intracellular vesicular structures, and a random budding pattern. Nonetheless, the profilin-deficient phenotype can be suppressed by the Rho2 signaling pathway [84]. Rho2 is a small GTPase of the Rho/Rac family of Ras-like proteins, which is involved in the establishment of cell polarity and in microtubule assembly [85, 86]. The only suppressor that can correct the phenotype of the profilin-deficient *S*. *cerevisiae* strain is Rho2p, which suggests that the suppressor acts through the Rho2p signaling pathway to repolarize cortical actin patches [84]. The *C*. *neoformans* homologue for Rho2, Rho family GTPase–Rho104 (CNAG_06606), was identified here as a significant interacting protein of Cdc10 during heat stress (37°) (S2 Table). This implicates septin Cdc10 in cytoskeleton stability via its association with profilin and Rho2’s homologue. Furthermore, Rho2 has been identified as a component of the MAPK signaling pathway in *S*. *pombe* [87–89].

Rho2 is a target of the farnesyltransferase Cpp1 and functions upstream of the Pck2–Pmk1 MAPK signaling pathway [90]. Moreover, Rho2 is involved in the regulation of cell wall integrity [90]. The palmitoylation of Rho2 enhances its plasma membrane localization and function, which is essential for signaling to the cell integrity pathway during vegetative growth and in response to stress. In the context of the MAPK pathway, Rho2 and Rho3 regulate the cell integrity MAPK pathway in an antagonistic fashion. The palmitoylation of Rho2 by Erf2 is essential for the signaling to the cell integrity pathway [91]. It is plausible that Cdc10’s interaction with the Rho2 homologue in *C*. *neoformans* during heat stress is important for Rho2’s function at the plasma membrane and subsequent MAPK signaling. Furthermore, the MAPK signaling was among the significantly enriched KEGG pathways identified here by the shared interactome functional enrichment analysis (Fig 9B).

In our study, Cdc10 and Cdc3 also associated with other crucial elements of the MAPK pathway including a Rab GTPase and a Rho GTPase activator [92, 93]. Cdc3 associated significantly with the Rab family protein (CNAG_04771) during both ambient temperature (∼25°C), and heat stress (37°C) (Fig 7 and S8 Fig in S1 File). In *S*. *cerevisiae*, a Rab escort protein has been identified as a regulator of the MAPK pathway that controls filamentous growth [93].

During ambient temperature (∼25°C), both septin Cdc10 and Cdc3 were found to interact with the Rho GTPase activator, Lrg1 (CNAG_05703) (Tables 4, 5 and Fig 8). The homologue of this protein in *S*. *cerevisiae* is a GTPase-activating protein (GAP) that contains a Rho1p-specific GAP activity. Furthermore, it is a negative regulator of the Pkc1 mediated cell wall integrity signaling pathway, and a negative regulator of cell wall 1,3-β-glucan biosynthesis [94–96]. This further implicates a functional connection between septins and components of the MAPK signaling pathway involved in cell wall integrity in *C*. *neoformans*. Moreover, Rho1 is involved in septum formation in fission yeast [86]. Remarkably, the Rho GTPase activator Lrg1 also controls small GTPase Cdc42 in *S*. *pombe* and *Candida albicans* [97–99]. Therefore, Lrg1 is not only involved in maintaining cell wall integrity in these fungi but it also interacts with Cdc42, which is a key component in the regulation of septin filaments [100]. In *C*. *neoformans*, loss of Cdc42 function results in cytokinesis defects due to loss of the organization of septin proteins [101, 102]. Thus, the putative interaction of Cdc3 and Cdc10 with the Rho GTPase activator Lrg1 suggests a link between septins and cell wall integrity via the MAPK pathway.

In *C*. *neoformans*, the MAPK pathway is involved in regulating virulence and environmental adaptation. Activation of the pathway in response to host signals and environmental cues leads to the regulation of genes involved in capsule formation, melanin production, and stress responses [103, 104]. Thus, its presence in the septin interactome suggests potential involvement in cell wall maintenance and repair, which are pivotal for fungal survival. Additionally, given the interactions between septins and the plasma membrane, the MAPK pathway’s association may signify a regulatory mechanism that ensures plasma membrane stability and lipid homeostasis under various environmental conditions. In *C*. *albicans*, septin proteins have been found to scaffold cell-wall proteins via the cell wall integrity (CWI) MAPK pathway [12, 14, 105, 106]. Additionally, septin proteins in *Aspergillus nidulans* promote cell wall integrity and homeostasis in lipid metabolism through crosstalk with the MAPK CWI pathway [107]. Thus, the putative cross-regulatory relationship between septin proteins and the MAPK pathway in *C*. *neoformans* warrants further investigation and may provide insights into cryptococcal adaptability and pathogenicity.

### Association of septins with Afr1

Our findings suggest that both Cdc3 and Cdc10 associate with Afr1 and these interactions are more pronounced at 37°C. Importantly, GFP-Afr1 exhibited localization in *C*. *neoformans* that is consistent with the predicted association with the plasma membrane (Fig 10). ABC transporters are a large family of membrane-associated proteins that use ATP to pump substrates across cellular membranes [108–110]. The Afr1 (Antifungal Resistance 1) protein, a member of the ABC family, functions as an efflux pump that exports antifungal drugs like fluconazole out of fungal cells, thereby reducing their intracellular accumulation and conferring resistance [111]. Overexpression of *AFR1* leads to upregulation of this efflux activity, resulting in high-level fluconazole resistance in *C*. *neoformans* both *in vitro* and *in vivo* [112–114]. Interestingly, beyond mediating drug resistance, the Afr1 protein also appears to contribute directly to the virulence of *C*. *neoformans*. Strains overexpressing *AFR1* were found to be significantly more virulent than wild-type in mouse models of cryptococcosis, regardless of the infection route (intravenous or inhalational). This enhanced virulence was linked to the increased intracellular survival of these strains inside macrophages, which are key immune cells that normally kill and clear *C*. *neoformans* [62, 112, 113]. Intriguingly, our microscopy data suggest that at 37°C, septins accumulate at the plasma membrane where they may interact with Afr1 and other stress related proteins and contribute to stress response (Fig 10). Therefore, the association of septin proteins with Afr1 during heat stress suggests that septins might contribute to virulence via a pathway independent of their role in cytokinesis that may involve localization to the plasma membrane, a possibility that should be addressed in future studies.

### Other notable potential septin-interacting proteins

Another important candidate protein for the shared interactome of Cdc10 and Cdc3 during both ambient temperature (∼25°C) and heat stress (37°C) is importin (CNAG_04973) (Fig 8). Notably, importins have been recently identified as regulators during meiosis and cytokinesis in a study by Beaudet, et al. [115]. In mammalian cells, the C-terminal nuclear localization sequence (NLS) of anillin is required for its localization and function during cytokinesis [115]. Furthermore, binding to active RhoA facilitates a conformational change in anillin that increases accessibility to the C-terminus, and importin binding stabilizes this conformation to facilitate anillin’s recruitment to the equatorial cortex. The study found that mutating the NLS alters anillin’s cortical dynamics. It also found that increasing active RhoA facilitates importin binding, while inactive RhoA or mutating the RhoA-binding domain (RBD) decreases importin binding. Anillin’s localization and function are abolished when NLS mutations are combined with additional point mutations in the C-terminus domain that weaken its interface with the RBD [115]. Thus, the relative position of the RBD and C-terminus is essential for anillin’s cortical recruitment, but also for ensuring that importin can be outcompeted by phospholipids and/or other cortical components [115]. In addition, septin recruitment in mammalian cells depends on anillin [116]. However, septins can also interact with F-actin and myosin II, which gives rise to multiple possibilities for how septins might be recruited to the contractile ring (CR) in an anillin-dependent manner. Septin recruitment to the CR appears to require the anillin C-terminus uniquely and specifically, which must be both competent to bind active RhoA-GTP and contain its PH domain. Thus, Rho1 GTPase controls anillo-septin assembly and facilitates contractile ring closure during cytokinesis [116]. Therefore, the link between importin and septin proteins suggests a potential mechanism for their coordinated regulation during cytokinesis. Importin binding to anillin stabilizes its conformation, facilitating its recruitment to the equatorial cortex [115]. This recruitment is crucial for anillin’s cortical dynamics and function [115]. Meanwhile, *Drosophila melanogaster*’s septin recruitment to the contractile ring also depends on anillin, specifically its C-terminus, which interacts with active RhoA-GTP and contains a PH domain [117, 118]. The Rho1 GTPase further controls anillo-septin assembly and facilitates contractile ring closure [119]. Thus, the existing literature supports the claim that importin is part of the septin complex interactome. The functional enrichment of the shared interactome of Cdc10 and Cdc3 revealed cytokinesis and cytoskeleton-dependent cytokinesis as GO terms with significant gene count and gene ratio (Fig 9A). Furthermore, cell cycle was among the significantly enriched KEGG pathways (Fig 9B). In *C*. *neoformans* anillin-like protein, a homologue of the *S*. *cerevisiae* Bud4, is crucial for proper organization and dynamics of the septin ring and the loss of Bud4 results in a phenotype strikingly similar to that of the septin deletion mutants [120]. The role of anillin-like proteins in septin organization during cytokinesis appears to be a conserved feature in fungi [16, 121–123]. Further studies investigating the interplay between importin, Bud4 homologue, and septins in *C*. *neoformans* will provide insights into the regulatory network governing cytokinesis in this species that may be conserved in other eukaryotes.

There are other noteworthy physical protein interactors of the septin proteins Cdc10 and Cdc3 that are also informative regarding septin function in *C*. *neoformans*. Several proteins of the DASH complex (Fig 8), which is involved with microtubules when the kinetochore attaches to the spindle, and plays a role in spindle attachment, chromosome segregation and spindle stability [124–126]. This might be an indication that septin proteins interact with the spindle stability and/or positioning during cytokinesis, similar to the roles implicated in *S*. *cerevisiae* [21]. In addition, several PH domain-containing proteins were identified as binding partners of both septin Cdc10 and Cdc3 (Fig 8) (Tables 6, 7), consistent with the association of septins with the plasma membrane in *C*. *neoformans*.

### Concluding remarks

This study constitutes the first comprehensive evaluation of the protein interactome of the septins Cdc3 and Cdc10 in *C*. *neoformans*, which should facilitate future investigations to determine the roles of septins in cell division and stress response in this fungal pathogen. In addition, this study has also provided the differentially expressed proteomic landscape of *C*. *neoformans* during heats tress and stationary growth phase compared to the exponential growth at ambient temperature control. Consequently, we have not only provided candidates for the proteins that belong to the septin complex interactome in *C*. *neoformans*, but we have also provided the context in which they may be interacting.

Fungal pathogens like *C*. *neoformans* encounter diverse environmental stressors during host colonization and infection. It is plausible that the putative association of the MAPK pathway with septins facilitates adaptive responses to these stressors. Septins are known to be involved in cytokinesis and morphogenesis, which are essential for pathogenicity. The MAPK pathway may modulate septin functions to aid in the fungus’s ability to adapt and thrive within the host. These interactions underscore the pivotal roles of septin proteins in mediating cellular responses to environmental stressors, potentially forming a regulatory nexus essential for *C*. *neoformans’* environmental adaptation and offering a framework for deeper insights at the mechanistic level. Furthermore, understanding the role of the MAPK pathway in the septin interactome has potential therapeutic implications, as disrupting this pathway could compromise cell wall integrity and plasma membrane stability, both essential for pathogenicity. The presence of the MAPK pathway within the septin interactome adds a layer of complexity to our understanding of fungal biology, particularly in the context of stress responses, cell wall integrity, and plasma membrane stability. Deciphering these intricate molecular relationships could deepen our understanding of fungal pathogenesis and offer new strategies for combating cryptococcal and other fungal infections.

## Supporting information

S1 File S1–S13 Figs. (PDF)

S1 Table Cdc10 binding partners at 25°C with logFC >2 and adjusted p-value of <0.05. (XLSX)

S2 Table Cdc10 binding partners at 37°C with logFC >2 and adjusted p-value of <0.05. (XLSX)

S3 Table Cdc3 binding partners at 25°C with logFC >2 and adjusted p-value of <0.05. (XLSX)

S4 Table Cdc3 binding partners at 37°C with logFC >2 and adjusted p-value of <0.05. (XLSX)

S1 Data The map of plasmid pSM1. (PDF)

S2 Data The map of plasmid pSM2. (PDF)

S3 Data The complete data set. (XLSX)

S1 Raw images Raw data related to Fig 10A–10D. (PDF)

## References

[1] Rajasingham R, Govender NP, Jordan A, Loyse A, Shroufi A, Denning DW, et al. The global burden of HIV-associated *cryptococcal* infection in adults in 2020: a modelling analysis. Lancet Infect Dis. 2022 Dec;22(12):1748–55. 36049486 10.1016/S1473-3099(22)00499-6 PMC9701154

[2] Zhao Y, Ye L, Zhao F, Zhang L, Lu Z, Chu T, et al. *Cryptococcus neoformans*, a global threat to human health. Infect Dis Poverty. 2023 Mar 17;12(1):20. 36932414 10.1186/s40249-023-01073-4 PMC10020775

[3] Idnurm A, Bahn YS, Nielsen K, Lin X, Fraser JA, Heitman J. Deciphering the model pathogenic fungus *Cryptococcus neoformans*. Nat Rev Microbiol. 2005;3(10):753–64. 16132036 10.1038/nrmicro1245

[4] Bojarczuk A, Miller KA, Hotham R, Lewis A, Ogryzko N V., Kamuyango AA, et al. *Cryptococcus neoformans* Intracellular Proliferation and Capsule Size Determines Early Macrophage Control of Infection. Sci Rep. 2016;6(January):1–15. 26887656 10.1038/srep21489 PMC4757829

[5] Mednick AJ, Nosanchuk JD, Casadevall A. Melanization of *Cryptococcus neoformans* affects lung inflammatory responses during *cryptococcal* infection. Infect Immun. 2005;73(4):2012–9. 15784542 10.1128/IAI.73.4.2012-2019.2005 PMC1087470

[6] Johnston SA, May RC. *Cryptococcus* interactions with macrophages: Evasion and manipulation of the phagosome by a fungal pathogen. Cell Microbiol. 2013;15(3):403–11. 23127124 10.1111/cmi.12067

[7] Ma H, Croudace JE, Lammas DA, May RC. Expulsion of Live Pathogenic Yeast by Macrophages. Current Biology. 2006;16(21):2156–60. doi: 10.1016/j.cub.2006.09.032 17084701

[8] Kozubowski L, Heitman J. Septins enforce morphogenetic events during sexual reproduction and contribute to virulence of *Cryptococcus neoformans*. Mol Microbiol. 2010 Feb;75(3):658–75. 19943902 10.1111/j.1365-2958.2009.06983.x PMC3699866

[9] Fung KYY, Dai L, Trimble WS. Cell and molecular biology of septins. 1st ed. Vol. 310, International Review of Cell and Molecular Biology. Elsevier Inc.; 2014. 289–339 p. 24725429 10.1016/B978-0-12-800180-6.00007-4

[10] Pan F, Malmberg RL, Momany M. Analysis of septins across kingdoms reveals orthology and new motifs. BMC Evol Biol. 2007;7:1–17. 17601340 10.1186/1471-2148-7-103 PMC1931588

[11] Weirich CS, Erzberger JP, Barral Y. The septin family of GTPases: Architecture and dynamics. Nat Rev Mol Cell Biol. 2008;9(6):478–89. doi: 10.1038/nrm2407 18478031

[12] Mostowy S, Cossart P. Septins: the fourth component of the cytoskeleton. Nat Rev Mol Cell Biol. 2012 Mar 8;13(3):183–94. doi: 10.1038/nrm3284 22314400

[13] Douglas LM, Alvarez FJ, Mccreary C, Konopka JB. Septin Function in Yeast Model Systems and Pathogenic Fungi MINIREVIEW Septin Function in Yeast Model Systems and Pathogenic Fungi. Society. 2005;4(9):1503–12. 10.1128/EC.4.9.1503-1512.2005 PMC1214204 16151244

[14] Gladfelter AS, Pringle JR, Lew DJ. The septin cortex at the yeast mother-bud neck. Curr Opin Microbiol. 2001 Dec 1;4(6):681–9. doi: 10.1016/s1369-5274(01)00269-7 11731320

[15] McMurray MA, Bertin A, Garcia G, Lam L, Nogales E, Thorner J. Septin Filament Formation Is Essential in Budding Yeast. Dev Cell. 2011 Apr 19;20(4):540–9. doi: 10.1016/j.devcel.2011.02.004 21497764 PMC3079881

[16] Chen X, Wang K, Svitkina T, Bi E. Critical Roles of a RhoGEF-Anillin Module in Septin Architectural Remodeling during Cytokinesis. Current Biology. 2020 Apr;30(8):1477–1490.e3. doi: 10.1016/j.cub.2020.02.023 32197082 PMC7176533

[17] Bi E, Maddox P, Lew DJ, Salmon ED, McMillan JN, Yeh E, et al. Involvement of an Actomyosin Contractile Ring in *Saccharomyces cerevisiae* Cytokinesis. J Cell Biol. 1998 Sep 7;142(5):1301–12. 9732290 10.1083/jcb.142.5.1301 PMC2149343

[18] Lippincott J, Li R. Dual Function of Cyk2, a cdc15/PSTPIP Family Protein, in Regulating Actomyosin Ring Dynamics and Septin Distribution. J Cell Biol. 1998 Dec 28;143(7):1947–60. doi: 10.1083/jcb.143.7.1947 9864366 PMC2175218

[19] Dobbelaere J, Barral Y. Spatial Coordination of Cytokinetic Events by Compartmentalization of the Cell Cortex. Science (1979). 2004 Jul 16;305(5682):393–6. doi: 10.1126/science.1099892 15256669

[20] Bi E, Park HO. Cell polarization and cytokinesis in budding yeast. Genetics. 2012 Jun;191(2):347–87. doi: 10.1534/genetics.111.132886 22701052 PMC3374305

[21] Castillon GA, Adames NR, Rosello CH, Seidel HS, Longtine MS, Cooper JA, et al. Septins Have a Dual Role in Controlling Mitotic Exit in Budding Yeast. Current Biology. 2003 Apr;13(8):654–8. doi: 10.1016/s0960-9822(03)00247-1 12699621

[22] Versele M, Thorner J. Septin collar formation in budding yeast requires GTP binding and direct phosphorylation by the PAK, Cla4. Journal of Cell Biology. 2004 Mar 1;164(5):701–15. doi: 10.1083/jcb.200312070 14993234 PMC2172161

[23] Wloka C, Nishihama R, Onishi M, Oh Y, Hanna J, Pringle JR, et al. Evidence that a septin diffusion barrier is dispensable for cytokinesis in budding yeast. bchm. 2011 Aug 1;392(8–9):813–29. doi: 10.1515/BC.2011.083 21824009

[24] Glomb O, Gronemeyer T. Septin organization and functions in budding yeast. Front Cell Dev Biol. 2016;4(NOV):1–6. doi: 10.3389/fcell.2016.00123 27857941 PMC5093138

[25] Fares H, Goetsch L, Pringle JR. Identification of a developmentally regulated septin and involvement of the septins in spore formation in *Saccharomyces cerevisiae*. J Cell Biol. 1996 Feb;132(3):399–411. 8636217 10.1083/jcb.132.3.399 PMC2120726

[26] De Virgilio C, DeMarini DJ, Pringle JR. SPR28, a sixth member of the septin gene family in *Saccharomyces cerevisiae* that is expressed specifically in sporulating cells. Microbiology (Reading). 1996 Oct;142 (Pt 10):2897–905. 8885406 10.1099/13500872-142-10-2897

[27] Tanaka-Takiguchi Y, Kinoshita M, Takiguchi K. Septin-Mediated Uniform Bracing of Phospholipid Membranes. Current Biology. 2009;19(2):140–5. doi: 10.1016/j.cub.2008.12.030 19167227

[28] Bridges AA, Zhang H, Mehta SB, Occhipinti P, Tani T, Gladfelter AS. Septin assemblies form by diffusion-driven annealing on membranes. Proc Natl Acad Sci U S A. 2014;111(6):2146–51. doi: 10.1073/pnas.1314138111 24469790 PMC3926015

[29] Bridges AA, Jentzsch MS, Oakes PW, Occhipinti P, Gladfelter AS. Micron-scale plasma membrane curvature is recognized by the septin cytoskeleton. Journal of Cell Biology. 2016;213(1):23–32. doi: 10.1083/jcb.201512029 27044896 PMC4828694

[30] Yi-Wei Huang, Ming Yan, Richard F. Collins, Jessica E. DiCiccio, Sergio Grinstein and WST. Mammalian Septins Are Required for Phagosome Formation. Mol Biol Cell. 2008;19(April):1717–26. 10.1091/mbc.E07-07-0641 PMC2291437 18272790

[31] Tokhtaeva E, Capri J, Marcus EA, Whitelegge JP, Khuzakhmetova V, Bukharaeva E, et al. Septin dynamics are essential for exocytosis. Journal of Biological Chemistry. 2015;290(9):5280–8297. doi: 10.1074/jbc.M114.616201 25575596 PMC4342448

[32] Qicong Hu, Ljiljana Milenkovic, Hua Jin, Matthew P. Scott, Maxence V. Nachury, Elias T. Spiliotis and WJN. A Septin Diffusion Barrier at the Base of the Primary Cilium Maintains Ciliary Membrane Protein Distribution. Science (1979). 2011;23(July):1–7. 10.1126/science.1191054 PMC3092790 20558667

[33] Estey MP, Kim MS, Trimble WS. Septins. Current Biology. 2011;21(10):384–7. doi: 10.1016/j.cub.2011.03.067 21601794

[34] Nakahira M, Macedo JNA, Seraphim TV, Cavalcante N, Souza TACB, Damalio JCP, et al. A Draft of the Human Septin Interactome. PLoS One. 2010;5(11). doi: 10.1371/journal.pone.0013799 21082023 PMC2970546

[35] Gönczi M, Dienes B, Dobrosi N, Fodor J, Balogh N, Oláh T, et al. Septins, a cytoskeletal protein family, with emerging role in striated muscle. J Muscle Res Cell Motil. 2020;(Cc). doi: 10.1007/s10974-020-09573-8 31955380 PMC8332580

[36] Neubauer K, Zieger B. The Mammalian Septin Interactome. Front Cell Dev Biol. 2017;5(February):1–9. 28224124 10.3389/fcell.2017.00003 PMC5293755

[37] Barve G, Sridhar S, Aher A, Sahani MH, Chinchwadkar S, Singh S, et al. Septins are involved at the early stages of macroautophagy in *S*. *cerevisiae*. J Cell Sci. 2018 Feb 1;131(4). 10.1242/jcs.209098 PMC5868950 29361537

[38] Barve G, Sanyal P, Manjithaya R. Septin localization and function during autophagy. Curr Genet. 2018 Oct 12;64(5):1037–41. doi: 10.1007/s00294-018-0834-8 29651536

[39] Renz C, Oeljeklaus S, Grinhagens S, Warscheid B, Johnsson N, Gronemeyer T. Identification of cell cycle dependent interaction partners of the septins by quantitative mass spectrometry. PLoS One. 2016;11(2):1–21. doi: 10.1371/journal.pone.0148340 26871441 PMC4752459

[40] Kozubowski L, Aboobakar EF, Cardenas ME, Heitman J. Calcineurin Colocalizes with P-Bodies and Stress Granules during Thermal Stress in *Cryptococcus neoformans*. Eukaryot Cell. 2011 Nov;10(11):1396–402. 21724937 10.1128/EC.05087-11 PMC3209054

[41] Davidson RC, Cruz MC, Sia RAL, Allen B, Alspaugh JA, Heitman J. Gene Disruption by Biolistic Transformation in Serotype D Strains of *Cryptococcus neoformans*. Fungal Genetics and Biology. 2000 Feb;29(1):38–48. 10779398 10.1006/fgbi.1999.1180

[42] Dena L. Toffaletti, Jennifer L. Tenor, John R. Perfect. Biolistic Transformation of *Cryptococcus neoformans*. In: McClelland EE, editor. Cryptococcus neoformans: Methods and Protocols, Methods in Molecular Biology. New York, NY: Springer US; 2024. p. 59–79. 10.1007/978-1-0716-3722-7_5 38758311

[43] Perfect JR, Ketabchi N, Cox GM, Ingram CW. Karyotyping of *Cryptococcus neoformans* as an Epidemiological Tool. J Clin Microbiol. 1993;31(12):3305–9. 8308124 10.1128/jcm.31.12.3305-3309.1993 PMC266409

[44] Byrum SD, Burdine MS, Orr L, Mackintosh SG, Authier S, Pouliot M, et al. Time-and radiation-dose dependent changes in the plasma proteome after total body irradiation of non-human primates: Implications for biomarker selection. PLoS One. 2017;12(3):1–15. doi: 10.1371/journal.pone.0174771 28350824 PMC5370149

[45] Byrum SD, Loughran AJ, Beenken KE, Orr LM, Storey AJ, Mackintosh SG, et al. Label-Free Proteomic Approach to Characterize Protease-Dependent and -Independent Effects of sarA Inactivation on the *Staphylococcus aureus* Exoproteome. J Proteome Res. 2018;17(10):3384–95. 30209945 10.1021/acs.jproteome.8b00288 PMC6209314

[46] Vazquez JH, Clemens MM, Allard FD, Yee EU, Kennon-McGill S, Mackintosh SG, et al. Identification of Serum Biomarkers to Distinguish Hazardous and Benign Aminotransferase Elevations. Toxicological Sciences. 2019 Oct 25;173(2):244–54. 10.1093/toxsci/kfz222 PMC8445655 31651977

[47] Searle BC, Pino LK, Egertson JD, Ting YS, Lawrence RT, MacLean BX, et al. Chromatogram libraries improve peptide detection and quantification by data independent acquisition mass spectrometry. Nat Commun. 2018 Dec 3;9(1):5128. doi: 10.1038/s41467-018-07454-w 30510204 PMC6277451

[48] Janbon G, Ormerod KL, Paulet D, Byrnes EJ, Yadav V, Chatterjee G, et al. Analysis of the genome and transcriptome of *Cryptococcus neoformans var*. *grubii* reveals complex RNA expression and microevolution leading to virulence attenuation. Freitag M, editor. PLoS Genet. 2014 Apr 17;10(4):e1004261. 24743168 10.1371/journal.pgen.1004261 PMC3990503

[49] Thurman TJ, Washam CL, Alkam D, Bird JT, Gies A, Dhusia K, et al. proteoDA: a package for quantitative proteomics. J Open Source Softw [Internet]. 2023 May 30;8(85):5184. Available from: https://joss.theoj.org/papers/10.21105/joss.05184

[50] Ritchie ME, Phipson B, Wu D, Hu Y, Law CW, Shi W, et al. limma powers differential expression analyses for RNA-sequencing and microarray studies. Nucleic Acids Res. 2015 Apr 20;43(7):e47–e47. doi: 10.1093/nar/gkv007 25605792 PMC4402510

[51] Babicki S, Arndt D, Marcu A, Liang Y, Grant JR, Maciejewski A, et al. Heatmapper: web-enabled heat mapping for all. Nucleic Acids Res. 2016 Jul 8;44(W1):W147–53. doi: 10.1093/nar/gkw419 27190236 PMC4987948

[52] Subramanian A, Tamayo P, Mootha VK, Mukherjee S, Ebert BL, Gillette MA, et al. Gene set enrichment analysis: A knowledge-based approach for interpreting genome-wide expression profiles. Proceedings of the National Academy of Sciences. 2005 Oct 25;102(43):15545–50. doi: 10.1073/pnas.0506580102 16199517 PMC1239896

[53] Al-Shahrour F, Díaz-Uriarte R, Dopazo J. FatiGO: a web tool for finding significant associations of Gene Ontology terms with groups of genes. Bioinformatics. 2004 Mar 1;20(4):578–80. doi: 10.1093/bioinformatics/btg455 14990455

[54] Schneider CA, Rasband WS, Eliceiri KW. NIH Image to ImageJ: 25 years of image analysis. Nat Methods. 2012 Jul 28;9(7):671–5. doi: 10.1038/nmeth.2089 22930834 PMC5554542

[55] Schindelin J, Arganda-Carreras I, Frise E, Kaynig V, Longair M, Pietzsch T, et al. Fiji: an open-source platform for biological-image analysis. Nat Methods. 2012 Jul 28;9(7):676–82. doi: 10.1038/nmeth.2019 22743772 PMC3855844

[56] Hecht M, Rösler R, Wiese S, Johnsson N, Gronemeyer T. An interaction network of the human SEPT9 established by quantitative mass spectrometry. G3: Genes, Genomes, Genetics. 2019;9(6):1869–80. doi: 10.1534/g3.119.400197 30975701 PMC6553528

[57] Szklarczyk D, Kirsch R, Koutrouli M, Nastou K, Mehryary F, Hachilif R, et al. The STRING database in 2023: protein–protein association networks and functional enrichment analyses for any sequenced genome of interest. Nucleic Acids Res. 2023 Jan 6;51(D1):D638–46. doi: 10.1093/nar/gkac1000 36370105 PMC9825434

[58] Van Andel E, Roosjen M, Van der Zanden S, Lange SC, Weijers D, Smulders MMJ, et al. Highly Specific Protein Identification by Immunoprecipitation–Mass Spectrometry Using Antifouling Microbeads. ACS Appl Mater Interfaces. 2022 May 25;14(20):23102–16. doi: 10.1021/acsami.1c22734 35536557 PMC9136845

[59] Smits AH, Jansen PWTC, Poser I, Hyman AA, Vermeulen M. Stoichiometry of chromatin-associated protein complexes revealed by label-free quantitative mass spectrometry-based proteomics. Nucleic Acids Res. 2013 Jan 1;41(1):e28–e28. doi: 10.1093/nar/gks941 23066101 PMC3592467

[60] Carlsson L, Nyström LE, Sundkvist I, Markey F, Lindberg U. Actin polymerizability is influenced by profilin, a low molecular weight protein in non-muscle cells. J Mol Biol. 1977 Sep;115(3):465–83. doi: 10.1016/0022-2836(77)90166-8 563468

[61] Posteraro B, Sanguinetti M, Sanglard D, La Sorda M, Boccia S, Romano L, et al. Identification and characterization of a *Cryptococcus neoformans* ATP binding cassette (ABC) transporter‐encoding gene, *CnAFR1*, involved in the resistance to fluconazole. Mol Microbiol. 2003 Jan 10;47(2):357–71. 12519188 10.1046/j.1365-2958.2003.03281.x

[62] Sanguinetti M, Posteraro B, La Sorda M, Torelli R, Fiori B, Santangelo R, et al. Role of *AFR1*, an ABC Transporter-Encoding Gene, in the In Vivo Response to Fluconazole and Virulence of *Cryptococcus neoformans*. Infect Immun. 2006 Feb;74(2):1352–9. 16428784 10.1128/IAI.74.2.1352-1359.2006 PMC1360305

[63] Kozubowski L, Lee SC, Heitman J. Signalling pathways in the pathogenesis of *Cryptococcus*. Cell Microbiol. 2009 Mar;11(3):370–80. 19170685 10.1111/j.1462-5822.2008.01273.x PMC3310389

[64] Stempinski PR, Zielinski JM, Dbouk NH, Huey ES, McCormack EC, Rubin AM, et al. Genetic contribution to high temperature tolerance in *Cryptococcus neoformans*. Genetics. 2021 Mar 3;217(1). 10.1093/genetics/iyaa009 PMC8045695 33683363

[65] Gibney PA, Lu C, Caudy AA, Hess DC, Botstein D. Yeast metabolic and signaling genes are required for heat-shock survival and have little overlap with the heat-induced genes. Proc Natl Acad Sci U S A. 2013 Nov 12;110(46):E4393–402. doi: 10.1073/pnas.1318100110 24167267 PMC3831991

[66] Yeh J, Haarer BK. Profilin is required for the normal timing of actin polymerization in response to thermal stress. FEBS Lett. 1996 Dec 2;398(2–3):303–7. doi: 10.1016/s0014-5793(96)01259-8 8977127

[67] Bezanilla M, Gladfelter AS, Kovar DR, Lee WL. Cytoskeletal dynamics: A view from the membrane. Journal of Cell Biology [Internet]. 2015 May 11;209(3):329–37. Available from: https://rupress.org/jcb/article/209/3/329/38164/Cytoskeletal-dynamics-A-view-from-the doi: 10.1083/jcb.201502062 25963816 PMC4427793

[68] Moens PDJ, Bagatolli LA. Profilin binding to sub-micellar concentrations of phosphatidylinositol (4,5) bisphosphate and phosphatidylinositol (3,4,5) trisphosphate. Biochimica et Biophysica Acta (BBA)—Biomembranes. 2007 Mar;1768(3):439–49. doi: 10.1016/j.bbamem.2006.12.012 17275780

[69] Lambrechts A, Jonckheere V, Dewitte D, Vandekerckhove J, Ampe C. Mutational analysis of human profilin I reveals a second PI(4,5)-P2 binding site neighbouring the poly(L-proline) binding site. BMC Biochem. 2002;3(1):12. 12052260 10.1186/1471-2091-3-12 PMC116585

[70] Lu PJ, Shieh WR, Rhee SG, Yin HL, Chen CS. Lipid Products of Phosphoinositide 3-Kinase Bind Human Profilin with High Affinity. Biochemistry. 1996 Jan 1;35(44):14027–34. doi: 10.1021/bi961878z 8909300

[71] Lassing I, Lindberg U. Specificity of the interaction between phosphatidylinositol 4,5-bisphosphate and the profilin:actin complex. J Cell Biochem. 1988 Jul;37(3):255–67. doi: 10.1002/jcb.240370302 2842351

[72] Lassing I, Lindberg U. Specific interaction between phosphatidylinositol 4,5-bisphosphate and profilactin. Nature. 1985 Apr 1;314(6010):472–4. doi: 10.1038/314472a0 2984579

[73] Goldschmidt-Clermont PJ, Kim JW, Machesky LM, Rhee SG, Pollard TD. Regulation of Phospholipase C-γ1 by Profilin and Tyrosine Phosphorylation. Science (1979). 1991 Mar 8;251(4998):1231–3. 10.1126/science.1848725 1848725

[74] Sagot I, Rodal AA, Moseley J, Goode BL, Pellman D. An actin nucleation mechanism mediated by Bni1 and profilin. Nat Cell Biol. 2002 Aug;4(8):626–31. doi: 10.1038/ncb834 12134165

[75] Figley MD, Bieri G, Kolaitis RM, Taylor JP, Gitler AD. Profilin 1 Associates with Stress Granules and ALS-Linked Mutations Alter Stress Granule Dynamics. Journal of Neuroscience. 2014 Jun 11;34(24):8083–97. doi: 10.1523/JNEUROSCI.0543-14.2014 24920614 PMC4051967

[76] Grousl T, Vojtova J, Hasek J, Vomastek T. Yeast stress granules at a glance. Yeast. 2022 Apr 30;39(4):247–61. doi: 10.1002/yea.3681 34791685

[77] Palangi F, Samuel SM, Thompson IR, Triggle CR, Emara MM. Effects of oxidative and thermal stresses on stress granule formation in human induced pluripotent stem cells. PLoS One. 2017 Jul 26;12(7):e0182059. doi: 10.1371/journal.pone.0182059 28746394 PMC5528897

[78] Fan AC, Leung AKL. RNA Granules and Diseases: A Case Study of Stress Granules in ALS and FTLD. In 2016. p. 263–96. 10.1007/978-3-319-29073-7_11 PMC5247449 27256390

[79] Park HS, Chow EWL, Fu C, Soderblom EJ, Moseley MA, Heitman J, et al. Calcineurin Targets Involved in Stress Survival and Fungal Virulence. PLoS Pathog. 2016 Sep;12(9):e1005873. doi: 10.1371/journal.ppat.1005873 27611567 PMC5017699

[80] Chen RE, Thorner J. Function and regulation in MAPK signaling pathways: Lessons learned from the yeast *Saccharomyces cerevisiae*. Biochimica et Biophysica Acta (BBA)—Molecular Cell Research. 2007 Aug;1773(8):1311–40. 17604854 10.1016/j.bbamcr.2007.05.003 PMC2031910

[81] WIDMANN C, GIBSON S, JARPE MB, JOHNSON GL. Mitogen-Activated Protein Kinase: Conservation of a Three-Kinase Module From Yeast to Human. Physiol Rev. 1999 Jan 1;79(1):143–80. doi: 10.1152/physrev.1999.79.1.143 9922370

[82] Prabhakar A, González B, Dionne H, Basu S, Cullen PJ. Spatiotemporal control of pathway sensors and cross-pathway feedback regulate a differentiation MAPK pathway in yeast. J Cell Sci. 2021 Aug 1;134(15). doi: 10.1242/jcs.258341 34347092 PMC8353523

[83] Correia I, Alonso-Monge R, Pla J. MAPK cell-cycle regulation in *Saccharomyces cerevisiae* and *Candida albicans*. Future Microbiol. 2010 Jul;5(7):1125–41. 20632810 10.2217/fmb.10.72

[84] Marcoux N, Cloutier S, Zakrzewska E, Charest PM, Bourbonnais Y, Pallotta D. Suppression of the Profilin-Deficient Phenotype by the RHO2 Signaling Pathway in *Saccharomyces cerevisiae*. Genetics. 2000 Oct 1;156(2):579–92. 11014808 10.1093/genetics/156.2.579 PMC1461282

[85] Madaule P, Axel R, Myers AM. Characterization of two members of the rho gene family from the yeast *Saccharomyces cerevisiae*. Proc Natl Acad Sci U S A. 1987 Feb;84(3):779–83. 3543936 10.1073/pnas.84.3.779 PMC304299

[86] Madden K, Snyder M. Cell polarity and morphogenesis in budding yeast. Annu Rev Microbiol. 1998;52:687–744. doi: 10.1146/annurev.micro.52.1.687 9891811

[87] Arellano M, Valdivieso MH, Calonge TM, Coll PM, Duran A, Perez P. *Schizosaccharomyces pombe* protein kinase C homologues, pck1p and pck2p, are targets of rho1p and rho2p and differentially regulate cell integrity. J Cell Sci. 1999 Oct;112 (Pt 20):3569–78. 10504305 10.1242/jcs.112.20.3569

[88] Iwaki N, Karatsu K, Miyamoto M. Role of guanine nucleotide exchange factors for Rho family GTPases in the regulation of cell morphology and actin cytoskeleton in fission yeast. Biochem Biophys Res Commun. 2003 Dec 12;312(2):414–20. doi: 10.1016/j.bbrc.2003.10.140 14637153

[89] Soto T, Villar-Tajadura MA, Madrid M, Vicente J, Gacto M, Pérez P, et al. Rga4 modulates the activity of the fission yeast cell integrity MAPK pathway by acting as a Rho2 GTPase-activating protein. J Biol Chem. 2010 Apr 9;285(15):11516–25. doi: 10.1074/jbc.M109.071027 20164182 PMC2857030

[90] Ma Y, Kuno T, Kita A, Asayama Y, Sugiura R. Rho2 Is a Target of the Farnesyltransferase Cpp1 and Acts Upstream of Pmk1 Mitogen-activated Protein Kinase Signaling in Fission Yeast. Mol Biol Cell. 2006 Dec;17(12):5028–37. doi: 10.1091/mbc.e06-08-0688 17005909 PMC1679671

[91] Sánchez-Mir L, Franco A, Martín-García R, Madrid M, Vicente-Soler J, Soto T, et al. Rho2 Palmitoylation Is Required for Plasma Membrane Localization and Proper Signaling to the Fission Yeast Cell Integrity Mitogen-Activated Protein Kinase Pathway. Mol Cell Biol. 2014 Jul 1;34(14):2745–59. doi: 10.1128/MCB.01515-13 24820419 PMC4097651

[92] Van Aelst L, D’Souza-Schorey C. Rho GTPases and signaling networks. Genes Dev [Internet]. 1997 Sep 15;11(18):2295–322. Available from: http://genesdev.cshlp.org/lookup/doi/10.1101/gad.11.18.2295 9308960 10.1101/gad.11.18.2295

[93] Jamalzadeh S, Pujari AN, Cullen PJ. A Rab escort protein regulates the MAPK pathway that controls filamentous growth in yeast. Sci Rep [Internet]. 2020 Dec 17;10(1):22184. Available from: https://www.nature.com/articles/s41598-020-78470-4 doi: 10.1038/s41598-020-78470-4 33335117 PMC7746766

[94] Müller L, Xu G, Wells R, Hollenberg CP, Piepersberg W. LRG1 is expressed during sporulation in *Saccharomyces cerevisiae* and contains motifs similar to LIM and rho/racGAP domains. Nucleic Acids Res. 1994 Aug 11;22(15):3151–4. 8065929 10.1093/nar/22.15.3151 PMC310289

[95] Lorberg A, Schmitz HP, Jacoby JJ, Heinisch JJ. Lrg1p functions as a putative GTPase-activating protein in the Pkc1p-mediated cell integrity pathway in *Saccharomyces cerevisiae*. Mol Genet Genomics. 2001 Nov;266(3):514–26. 11713681 10.1007/s004380100580

[96] Watanabe D, Abe M, Ohya Y. Yeast Lrg1p acts as a specialized RhoGAP regulating 1,3-beta-glucan synthesis. Yeast. 2001 Jul;18(10):943–51. doi: 10.1002/yea.742 11447600

[97] Tatebe H, Nakano K, Maximo R, Shiozaki K. Pom1 DYRK regulates localization of the Rga4 GAP to ensure bipolar activation of Cdc42 in fission yeast. Curr Biol. 2008 Mar 11;18(5):322–30. doi: 10.1016/j.cub.2008.02.005 18328707 PMC2277499

[98] Xie JL, Grahl N, Sless T, Leach MD, Kim SH, Hogan DA, et al. Signaling through Lrg1, Rho1 and Pkc1 Governs *Candida albicans* Morphogenesis in Response to Diverse Cues. PLoS Genet. 2016 Oct 27;12(10):e1006405. 27788136 10.1371/journal.pgen.1006405 PMC5082861

[99] Chen T, Wagner AS, Tams RN, Eyer JE, Kauffman SJ, Gann ER, et al. Lrg1 Regulates β (1,3)-Glucan Masking in *Candida albicans* through the Cek1 MAP Kinase Pathway. mBio. 2019 Oct 29;10(5). 10.1128/mBio.01767-19 PMC6751057 31530671

[100] Sadian Y, Gatsogiannis C, Patasi C, Hofnagel O, Goody RS, Farkašovský M, et al. The role of Cdc42 and Gic1 in the regulation of septin filament formation and dissociation. Elife. 2013 Nov 28;2. doi: 10.7554/eLife.01085 24286829 PMC3840788

[101] Ballou ER, Nichols CB, Miglia KJ, Kozubowski L, Alspaugh JA. Two CDC42 paralogues modulate *Cryptococcus neoformans* thermotolerance and morphogenesis under host physiological conditions. Mol Microbiol. 2010 Feb;75(3):763–80. 20025659 10.1111/j.1365-2958.2009.07019.x PMC3685590

[102] Ballou ER, Kozubowski L, Nichols CB, Alspaugh JA. Ras1 Acts through Duplicated Cdc42 and Rac Proteins to Regulate Morphogenesis and Pathogenesis in the Human Fungal Pathogen *Cryptococcus neoformans*. PLoS Genet. 2013 Aug 8;9(8):e1003687. 23950731 10.1371/journal.pgen.1003687 PMC3738472

[103] Jung KW, Bahn YS. The Stress-Activated Signaling (SAS) Pathways of a Human Fungal Pathogen, *Cryptococcus neoformans*. Mycobiology. 2009 Sep;37(3):161–70. 23983528 10.4489/MYCO.2009.37.3.161 PMC3749383

[104] de Oliveira HC, Rossi SA, García-Barbazán I, Zaragoza Ó, Trevijano-Contador N. Cell Wall Integrity Pathway Involved in Morphogenesis, Virulence and Antifungal Susceptibility in *Cryptococcus neoformans*. J Fungi (Basel). 2021 Oct 5;7(10). 10.3390/jof7100831 PMC8540506 34682253

[105] Blankenship JR, Fanning S, Hamaker JJ, Mitchell AP. An Extensive Circuitry for Cell Wall Regulation in *Candida albicans*. Hull CM, editor. PLoS Pathog [Internet]. 2010 Feb 5;6(2):e1000752. Available from: https://dx.plos.org/10.1371/journal.ppat.1000752 20140194 10.1371/journal.ppat.1000752 PMC2816693

[106] Caudron F, Barral Y. Septins and the Lateral Compartmentalization of Eukaryotic Membranes. Dev Cell [Internet]. 2009 Apr;16(4):493–506. Available from: https://linkinghub.elsevier.com/retrieve/pii/S1534580709001397 doi: 10.1016/j.devcel.2009.04.003 19386259

[107] Mela A, Momany M. Septins coordinate cell wall integrity and lipid metabolism in a sphingolipid-dependent process. J Cell Sci. 2022 Mar 1;135(5). 10.1242/jcs.258336 33912961

[108] Rees DC, Johnson E, Lewinson O. ABC transporters: the power to change. Nat Rev Mol Cell Biol. 2009 Mar;10(3):218–27. doi: 10.1038/nrm2646 19234479 PMC2830722

[109] Wilkens S. Structure and mechanism of ABC transporters. F1000Prime Rep. 2015 Feb 3;7. doi: 10.12703/P7-14 25750732 PMC4338842

[110] Flatt S, Busiello DM, Zamuner S, De Los Rios P. ABC transporters are billion-year-old Maxwell Demons. Commun Phys. 2023 Aug 8;6(1):205. doi: 10.1038/s42005-023-01320-y 38665399 PMC11041718

[111] Chang M, Sionov E, Khanal Lamichhane A, Kwon-Chung KJ, Chang YC. Roles of Three *Cryptococcus neoformans* and *Cryptococcus gattii* Efflux Pump-Coding Genes in Response to Drug Treatment. Antimicrob Agents Chemother. 2018 Apr;62(4). 10.1128/AAC.01751-17 PMC5913978 29378705

[112] Sionov E, Chang YC, Garraffo HM, Kwon-Chung KJ. Heteroresistance to Fluconazole in *Cryptococcus neoformans* Is Intrinsic and Associated with Virulence. Antimicrob Agents Chemother. 2009 Jul;53(7):2804–15. 19414582 10.1128/AAC.00295-09 PMC2704677

[113] Orsi CF, Colombari B, Ardizzoni A, Peppoloni S, Neglia R, Posteraro B, et al. The ABC transporter-encoding gene *AFR1* affects the resistance of *Cryptococcus neoformans* to microglia-mediated antifungal activity by delaying phagosomal maturation. FEMS Yeast Res. 2009 Mar;9(2):301–10. 19220870 10.1111/j.1567-1364.2008.00470.x

[114] Oliveira NK, Bhattacharya S, Gambhir R, Joshi M, Fries BC. Novel ABC Transporter Associated with Fluconazole Resistance in Aging of *Cryptococcus neoformans*. Journal of Fungi. 2022 Jun 28;8(7):677. 35887434 10.3390/jof8070677 PMC9320417

[115] Beaudet D, Pham N, Skaik N, Piekny A. Importin binding mediates the intramolecular regulation of anillin during cytokinesis. Mol Biol Cell. 2020 May 15;31(11):1124–39. doi: 10.1091/mbc.E20-01-0006 32238082 PMC7353161

[116] Carim SC, Hickson GRX. The Rho1 GTPase controls anillo-septin assembly to facilitate contractile ring closure during cytokinesis. iScience. 2023 Jun;26(6):106903. doi: 10.1016/j.isci.2023.106903 37378349 PMC10291328

[117] Liu J, Fairn GD, Ceccarelli DF, Sicheri F, Wilde A. Cleavage Furrow Organization Requires PIP2-Mediated Recruitment of Anillin. Current Biology. 2012 Jan;22(1):64–9. doi: 10.1016/j.cub.2011.11.040 22197245

[118] Oegema K, Savoian MS, Mitchison TJ, Field CM. Functional Analysis of a Human Homologue of the *Drosophila* Actin Binding Protein Anillin Suggests a Role in Cytokinesis. J Cell Biol. 2000 Aug 7;150(3):539–52. 10931866 10.1083/jcb.150.3.539 PMC2175195

[119] Sun L, Guan R, Lee IJ, Liu Y, Chen M, Wang J, et al. Mechanistic Insights into the Anchorage of the Contractile Ring by Anillin and Mid1. Dev Cell. 2015 May;33(4):413–26. doi: 10.1016/j.devcel.2015.03.003 25959226 PMC4449299

[120] Peng CA, Altamirano S, Paladugu N, Crowe LP, Aboobakar IF, Chandrasekaran S, et al. Role of the anillin-like protein in growth of *Cryptococcus neoformans* at human host temperature. Fungal Genetics and Biology [Internet]. 2022 May;160:103697. Available from: https://linkinghub.elsevier.com/retrieve/pii/S108718452200041X 35472450 10.1016/j.fgb.2022.103697 PMC12395795

[121] Tasto JJ, Morrell JL, Gould KL. An anillin homologue, Mid2p, acts during fission yeast cytokinesis to organize the septin ring and promote cell separation. J Cell Biol. 2003 Mar 31;160(7):1093–103. doi: 10.1083/jcb.200211126 12668659 PMC2172762

[122] Kang PJ, Hood-DeGrenier JK, Park HO. Coupling of septins to the axial landmark by Bud4 in budding yeast. J Cell Sci. 2013 Mar 1;126(Pt 5):1218–26. 23345395 10.1242/jcs.118521 PMC3635463

[123] Wu H, Guo J, Zhou YT, Gao XD. The anillin-related region of Bud4 is the major functional determinant for Bud4’s function in septin organization during bud growth and axial bud site selection in budding yeast. Eukaryot Cell. 2015 Mar;14(3):241–51. doi: 10.1128/EC.00268-14 25576483 PMC4346561

[124] Li J mei, Li Y, Elledge SJ. Genetic Analysis of the Kinetochore DASH Complex Reveals an Antagonistic Relationship with the Ras/Protein Kinase A Pathway and a Novel Subunit Required for Ask1 Association. Mol Cell Biol. 2005 Jan 1;25(2):767–78. doi: 10.1128/MCB.25.2.767-778.2005 15632076 PMC543429

[125] Miranda JL, Wulf P De, Sorger PK, Harrison SC. The yeast DASH complex forms closed rings on microtubules. Nat Struct Mol Biol. 2005 Feb 10;12(2):138–43. doi: 10.1038/nsmb896 15640796

[126] Foley EA, Kapoor TM. Microtubule attachment and spindle assembly checkpoint signalling at the kinetochore. Nat Rev Mol Cell Biol. 2013 Jan 21;14(1):25–37. doi: 10.1038/nrm3494 23258294 PMC3762224

