# Supplementary material for: Quantitative analysis of septin Cdc10 & Cdc3-associated proteome during stress response in the fungal pathogen Cryptococcus neoformans
Source: PLoS One. 2024 Dec 17;19(12):e0313444. doi: 10.1371/journal.pone.0313444 (PMC11651612; doi:10.1371/journal.pone.0313444)
Supplement: S1 File — (PDF) [file pone.0313444.s001.pdf]

S1Fig.

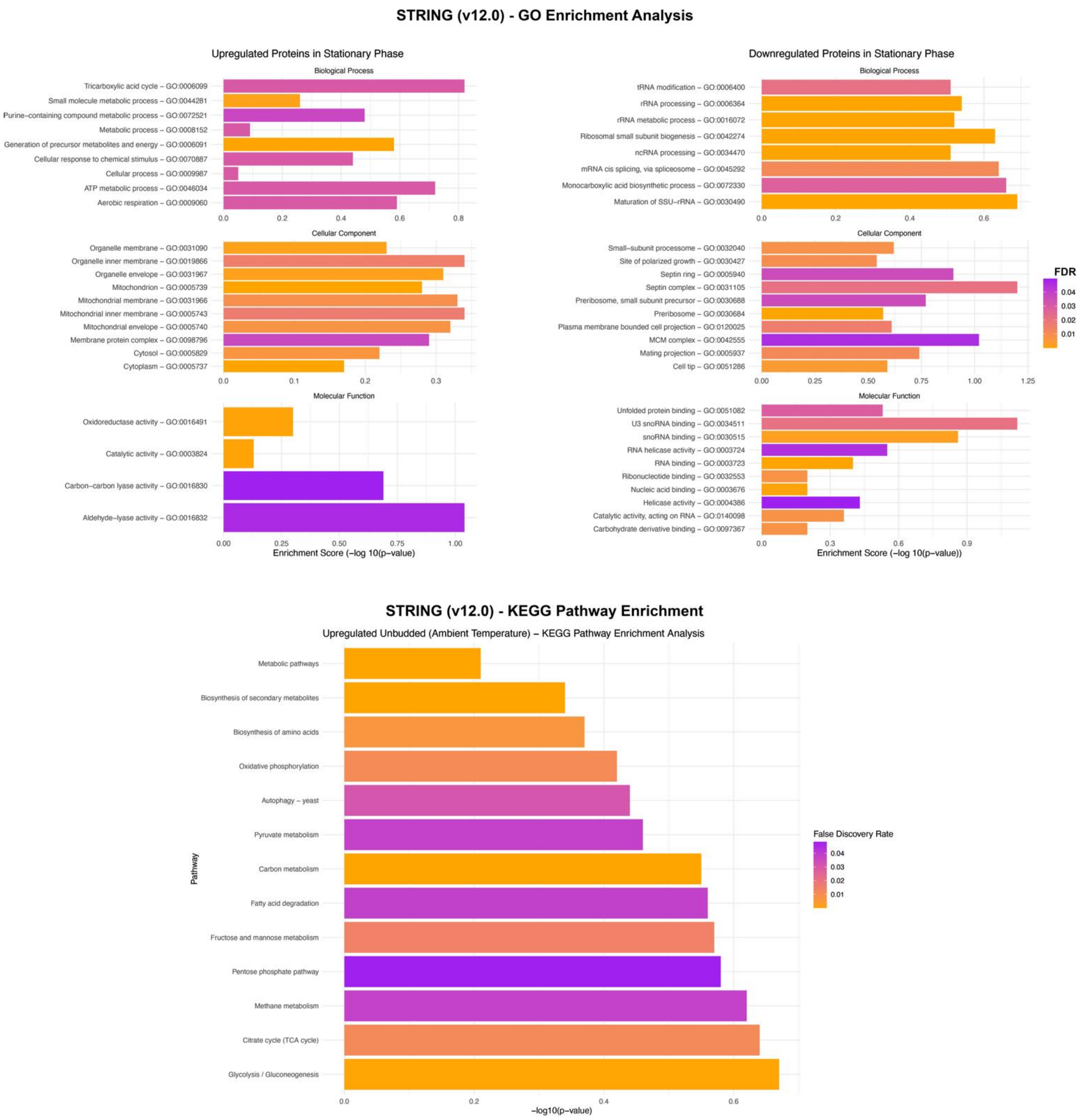

S1Fig. STRING gene set analysis of proteins differentially expressed during stationary growth phase. Top: Gene Ontology analysis of proteins significantly upregulated and downregulated during stationary growth phase. Bottom: KEGG pathway enrichment analysis of proteins significantly upregulated and downregulated during stationary growth phase.

S2Fig.

STRING (v12.0) - GO Enrichment Analysis

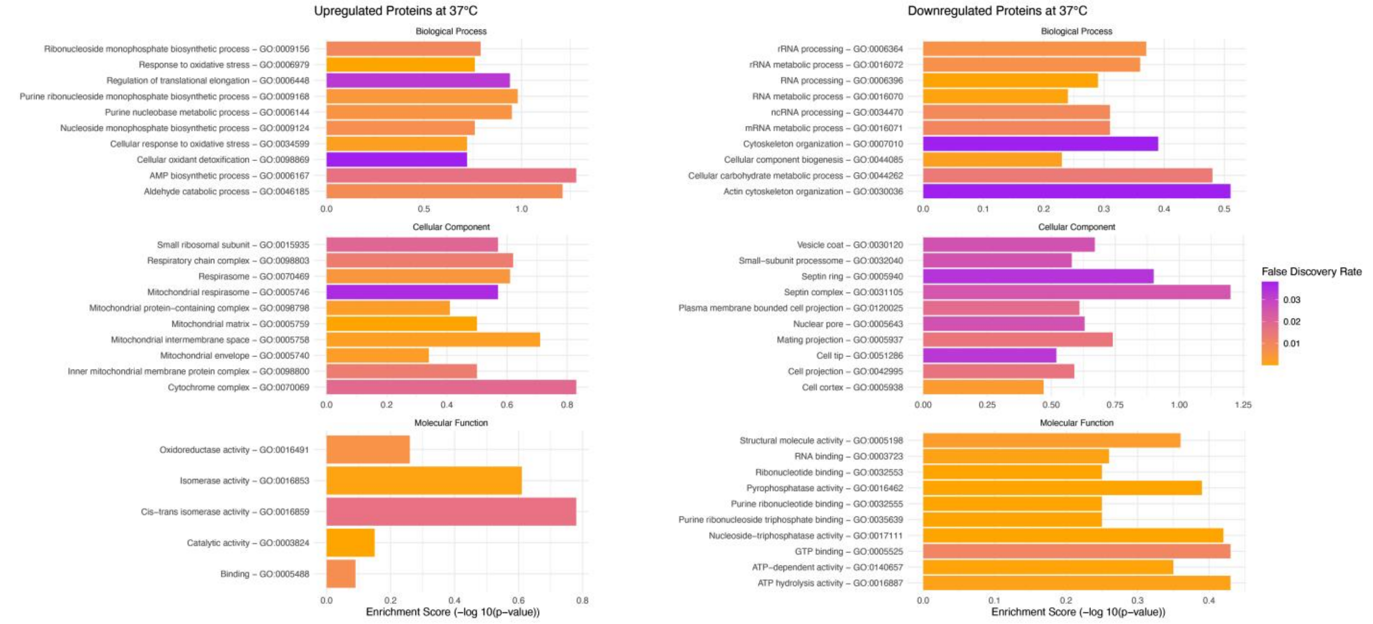

STRING (v12.0) - KEGG Pathway Enrichment

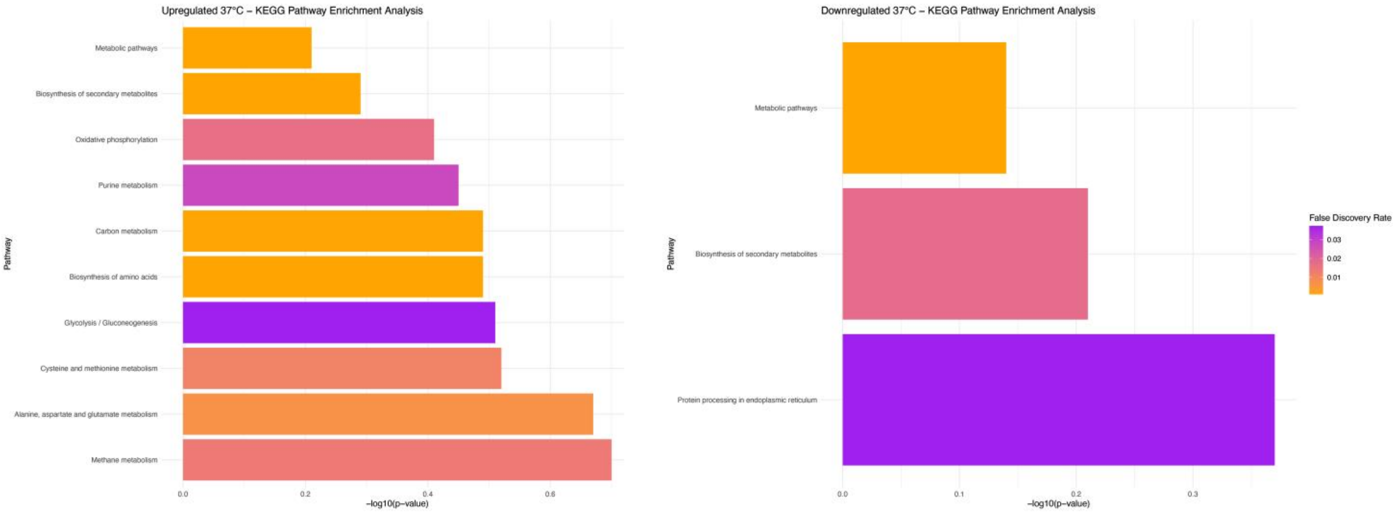

S2Fig. STRING gene set analysis of proteins differentially expressed during heat stress (37°C). Top: Gene Ontology analysis of proteins significantly upregulated and downregulated during heat stress (37°C). Bottom: KEGG pathway enrichment analysis of proteins significantly upregulated and downregulated during heat stress (37°C).

S3Fig.

A.

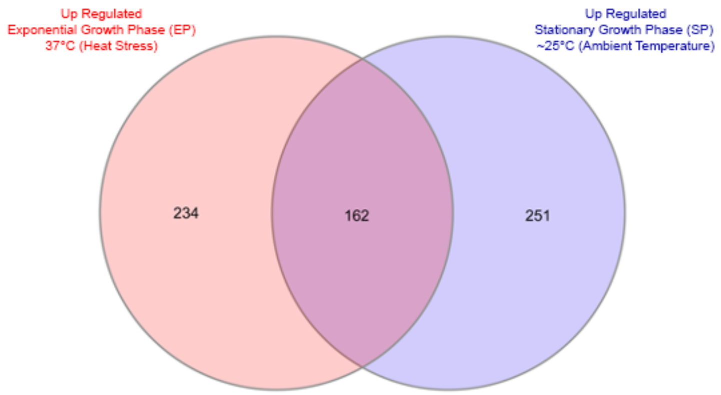

B.

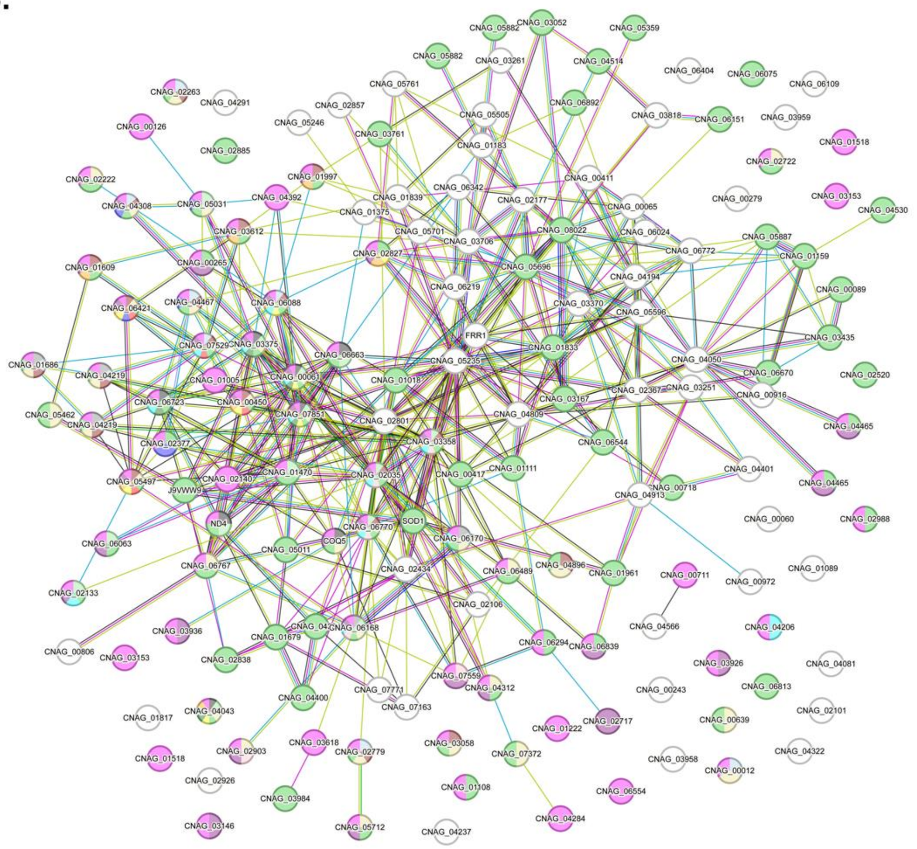

PPI enrichment p-value: 0.000168

KEEG Pathways Description	Strength	FDR
Valine, leucine and isoleucine biosynthesis	1.01	0.0222
Butanoate metabolism	0.96	0.0092
2-Oxocarboxylic acid metabolism	0.83	0.0013
Citrate Cycle (TCA)	0.79	0.0335
Glycolysis/ Gluconeogenesis	0.68	0.0369
Carbon metabolism	0.63	0.00052
Biosynthesis of amino acids	0.58	0.00069
Biosynthesis of secondary metabolites	0.42	5.26e-05
Metabolic Pathways	0.24	5.26e-05

Biological Process (GO) Description	Strength	FDR
Aldehyde catabolic process	1.58	0.0018
Aerobic Respiration	0.75	0.467
Generation of precursor metabolites and energy	0.72	0.00055
Small molecule catabolic process	0.57	0.0250
Carboxylic acid metabolic process	0.45	0.0077
Small molecule metabolic process	0.39	0.00055
Cellular metabolic process	0.14	0.0270

S3Fig. Overlap between upregulated proteins between the following conditions: heat stress and stationary growth phase (nutrient starvation). (A) Venn diagram showing shared proteins between upregulated in heat stress, and nutrient starvation conditions, respectively. (B) STRING protein-protein interaction (PPI) network of 162 proteins that are significantly upregulated during both heat stress and nutrient starvation. The PPI network nodes represent proteins, and are color coded according to STRING gene set analysis functional enrichment (KEGG Pathway and

Gene Ontology-Biological Process). The edges represent protein-protein associations: curated databases (light blue), magenta (experimentally determined), gene neighborhood (green), gene fusions (red), gene co-occurrence (dark blue), text mining (lime green), co-expression (black), and protein homology (light purple).

S4Fig.

A.

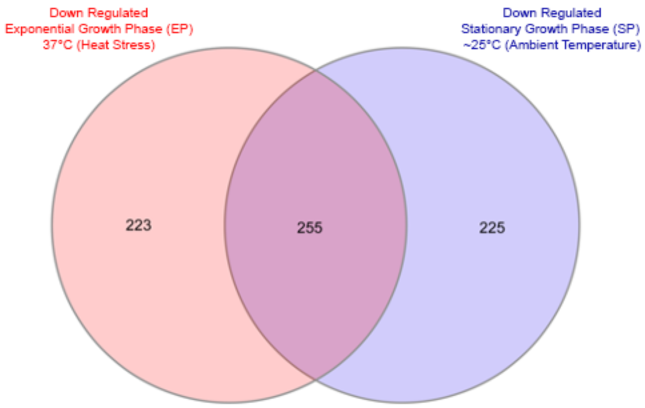

B.

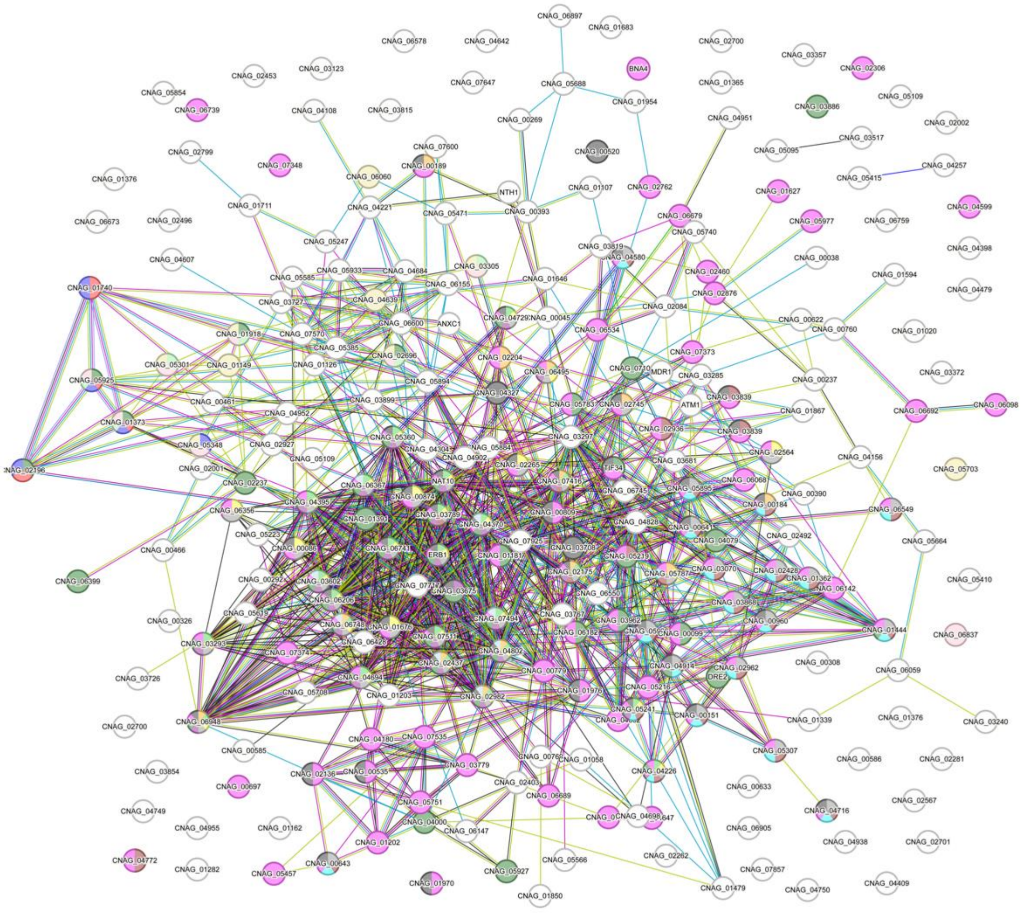

Biological Process (GO) Description	Strength	FDR
RNA phosphodiester bond hydrolysis	0.56	0.0133
rRNA processing	0.53	8.25e-05
ncRNA processing	0.47	2.81e-05
RNA processing	0.46	8.11e-08
mRNA metabolic process	0.46	0.00017
mRNA processing	0.45	0.0039
Ribosome biogenesis	0.42	0.0018
Cellular component biogenesis	0.29	0.00080
Cellular nitrogen compound metabolic process	0.26	4.06e-07

Molecular Function (GO) Description	Strength	FDR
RNA binding	10.39	9.82e-05

Cellular Component (GO) Description	Strength	FDR
Septin complex	1.47	0.0034
Septin ring	1.16	0.0038
Mating projection	0.88	0.0094
Plasma membrane bounded cell projection	0.78	0.0050
Small-subunit processome	0.74	0.0066
Cell tip	0.69	0.0062

S4Fig. Overlap between downregulated proteins between the following conditions: heat stress and stationary growth phase (nutrient starvation). (A) Venn diagram showing shared proteins between upregulated in heat stress, and nutrient starvation conditions, respectively. (B) STRING protein-protein interaction (PPI) network of 255 proteins that are significantly downregulated during both heat stress and nutrient starvation. The PPI network nodes represent proteins, and are color coded according to STRING gene set analysis functional enrichment (Gene Ontology Analysis).

The edges represent protein-protein associations: curated databases (light blue), magenta (experimentally determined), gene neighborhood (green), gene fusions (red), gene co-occurrence (dark blue), text mining (lime green), co-expression (black), and protein homology (light purple).

S5Fig.

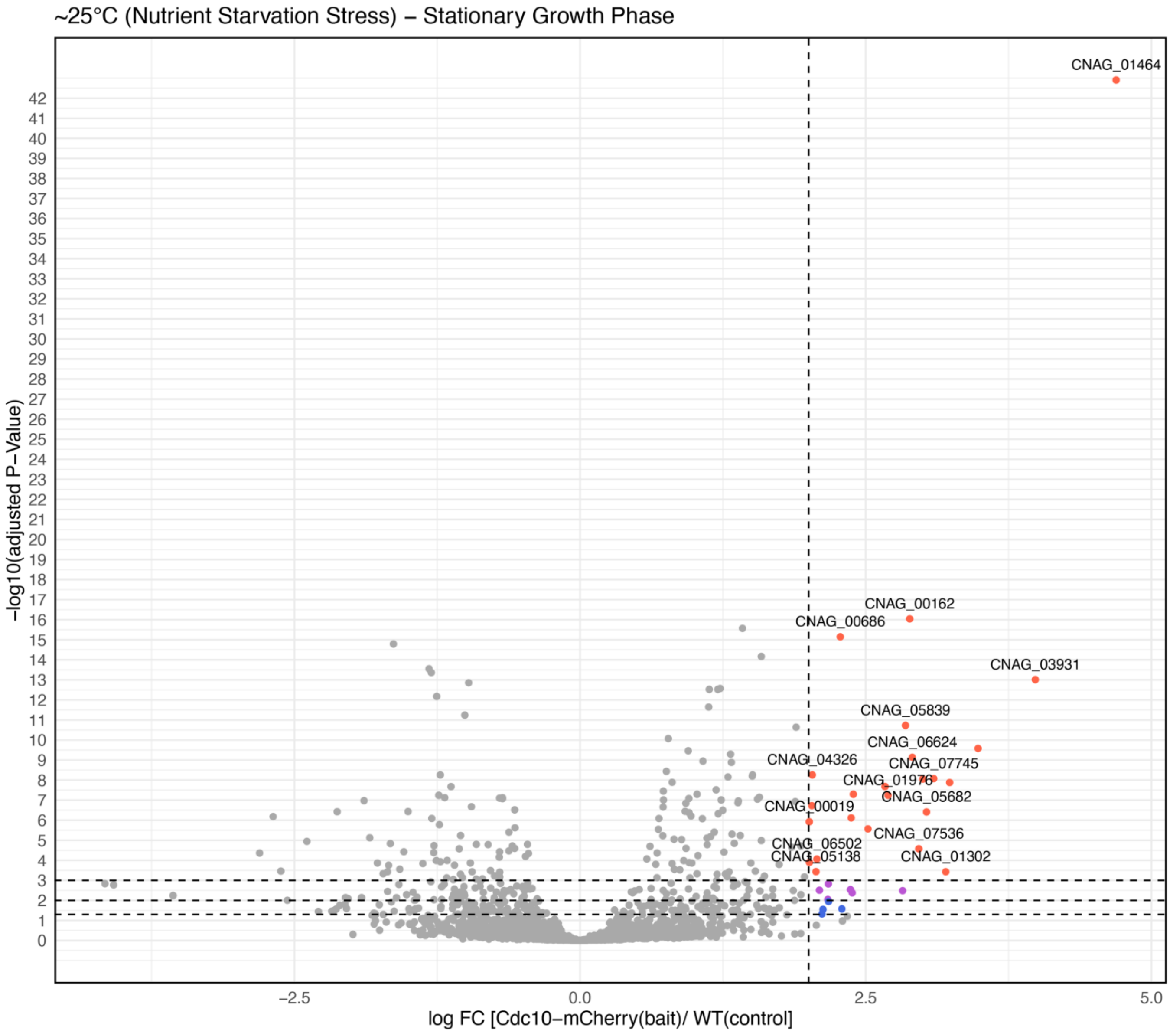

S5Fig. Volcano plot of protein interactome results showing proteins that are significantly enriched by Cdc10-mCherry co- immunoprecipitation in stationary phase growth at ambient temperature. Significant interacting partners were determined by statistical t-test using logFC >2 and FDR of 0.05. The most significant candidates (highlighted) were considered those with a logFC >2 and FDR of 0.001. Cdc10 (the bait) was not identified as a significant enriched protein in the pulldown comparison with the negative control Thus, the experiment was considered unsuccessful.

S6Fig.

A.

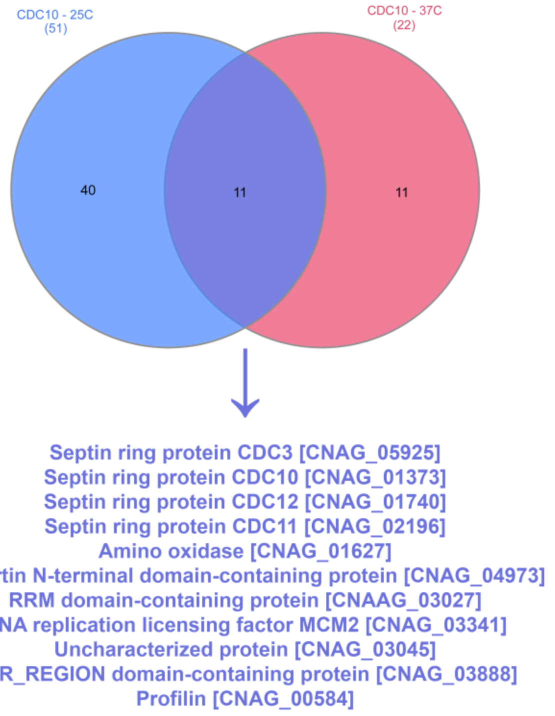

B.

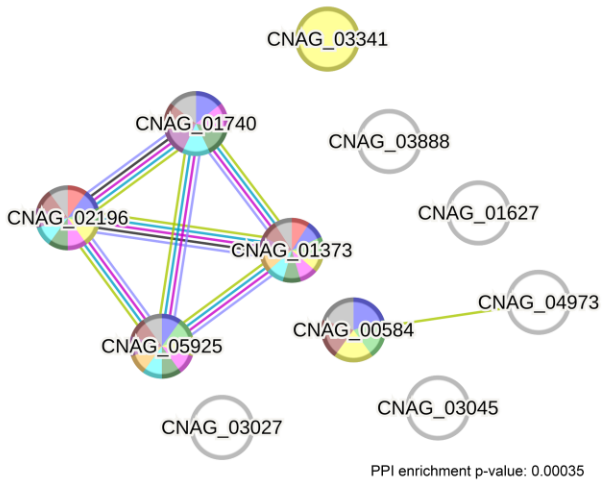

	STRING GO Term	Strength	FDR
	Septin ring organization	2.18	0.0243
	Cytoskeleton-dependent cytokinesis	2.11	1.27e-06
	Mitotic cytokinesis	2.05	5.42e-05
	Mitotic cell cycle process	1.31	0.0011
	Molecular adaptor activity	1.45	0.0124
	Septin complex	2.83	1.41e-07
	Septin ring	2.43	1.00e-06
	Intracellular immature response	2.09	0.0172
	Cell division site	1.69	0.00018
	Cell cortex	1.49	6.47e-05
	Cytoskeleton	1.2	0.0010

S6Fig. Overlap between the identified binding partners of septin Cdc10 during permissive growth temperature (~25°C) and heat stress (37°C). (A) Venn diagram displaying the shared interactome of Cdc10 during permissive growth temperature and heats stress. Only interacting partners with a **logFC >2 and adjusted p-value <0.001** were considered for this high stringency comparison. (B) STRING protein-protein interaction (PPI) network of **11 common proteins identified as consistent binding partners for septin Cdc10 during both permissive growth temperature and heat stress.** The PPI network nodes represent proteins, and are color coded according to STRING gene set analysis functional enrichment (Gene Ontology Analysis). The edges represent protein-protein associations: curated databases (light blue), magenta (experimentally determined), gene neighborhood (green), gene fusions (red), gene co-occurrence (dark blue), text mining (lime green), co-expression (black), and protein homology (light purple).

S7Fig.

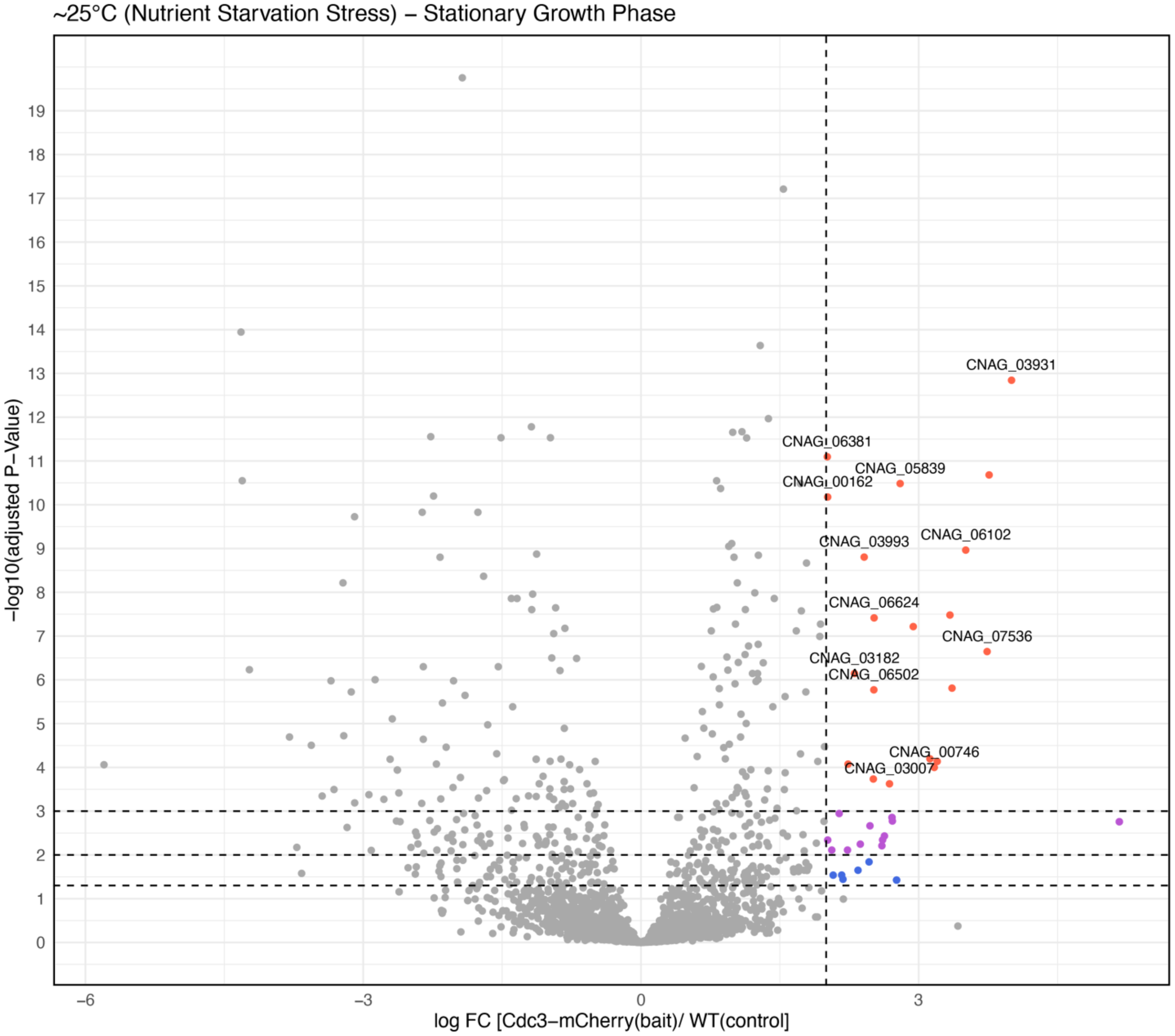

S7Fig. Volcano plot of protein interactome results showing proteins that are significantly enriched by Cdc3-mCherry co- immunoprecipitation in stationary phase growth at ambient temperature. Significant interacting partners were determined by statistical t-test using logFC >2 and FDR of 0.05. The most significant candidates (highlighted) were considered those with a logFC >2 and FDR of 0.001. Cdc3 (the bait) was not identified as a significant enriched protein in the pulldown comparison with the negative control Thus, the experiment was considered unsuccessful.

S8Fig.

A.

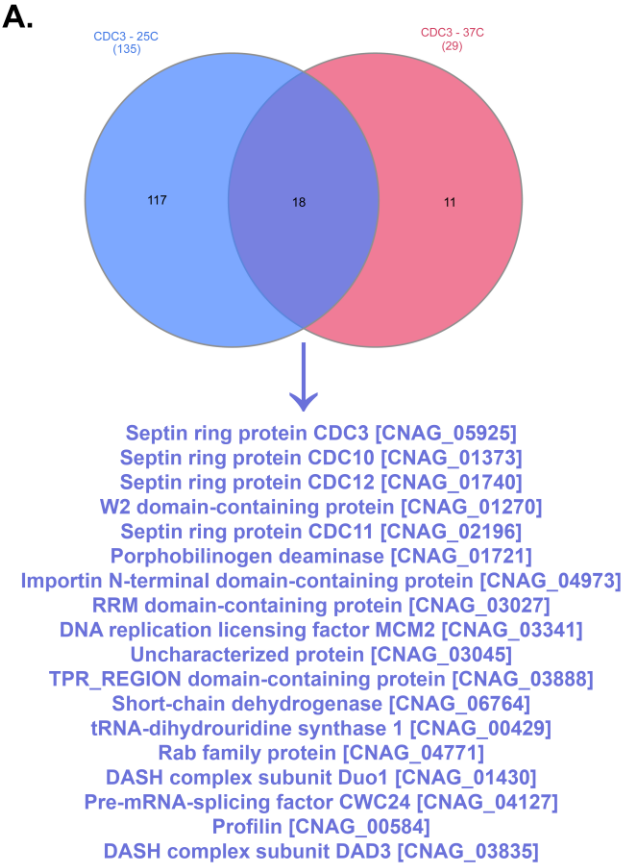

B.

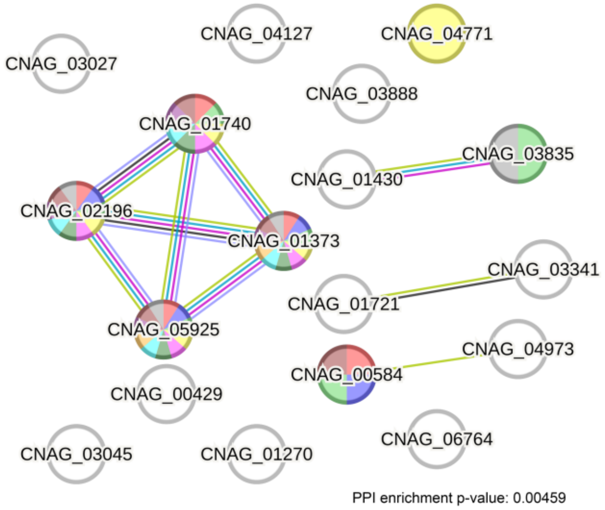

	STRING GO Term	Strength	FDR
	Cytoskeleton-dependent cytokinesis	1.9	2.30e-05
	Mitotic cytokinesis	1.84	0.00037
	Cell division	1.35	0.00022
	GTPase activity	1.34	0.0040
	Molecular adaptor activity	1.24	0.022
	Septin complex	2.62	1.30e-06
	Septin ring	2.22	9.19e-06
	Intracellular immature spore	1.88	0.0415
	Cell division site	1.48	0.0013
	Cell cortex	1.28	0.0011
	Microtubule cytoskeleton	1.14	0.0215

S8Fig. Overlap between the identified binding partners of septin Cdc3 during permissive growth temperature (~25°C) and heat stress (37°C). (A) Venn diagram displaying the shared interactome of Cdc3 during permissive growth temperature and heats stress. Only interacting partners with a **logFC >2 and adjusted p-value <0.001** were considered for this high stringency comparison. (B) STRING protein-protein interaction (PPI) network of **18 common proteins identified as consistent binding partners for septin Cdc3 during both permissive growth temperature and heat stress**. The PPI network nodes represent proteins, and are color coded according to STRING gene set analysis functional enrichment (Gene Ontology Analysis). The edges represent protein-protein associations: curated databases (light blue), magenta (experimentally determined), gene neighborhood (green), gene fusions (red), gene co-occurrence (dark blue), text mining (lime green), co-expression (black), and protein homology (light purple).

S9Fig.

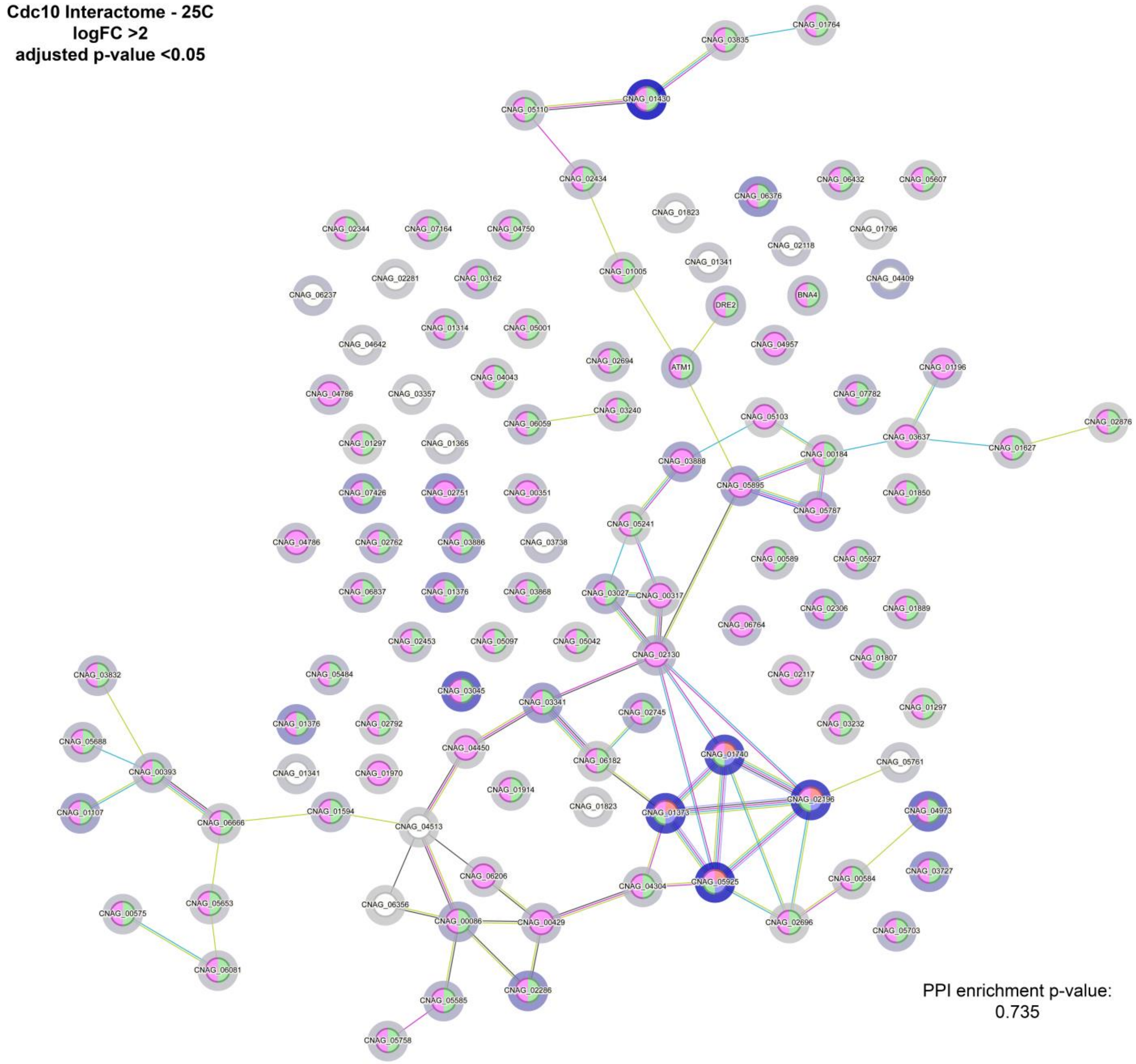

	Cellular Component GO Term	Strength	FDR
	Septin Complex	1.82	0.0024
	Septin Ring	1.42	0.0080
	Cytoplasm	0.16	0.0024
	Intracellular anatomical structure	0.11	0.0024

S9Fig. STRING protein-protein interaction (PPI) network of 112 proteins that are significantly enriched for Cdc10 pulldown at ambient temperature versus control (Supplementary Table 1). The PPI network nodes represent proteins, and are color coded according to STRING gene

set analysis functional enrichment (Gene Ontology-Cellular Component). The blue halo around each node represents the logFC enrichment value. The darker the halo, the higher the enrichment. The edges represent protein-protein associations: curated databases (light blue), magenta (experimentally determined), gene neighborhood (green), gene fusions (red), gene co-occurrence (dark blue), text mining (lime green), co-expression (black), and protein homology (light purple).

S10Fig.

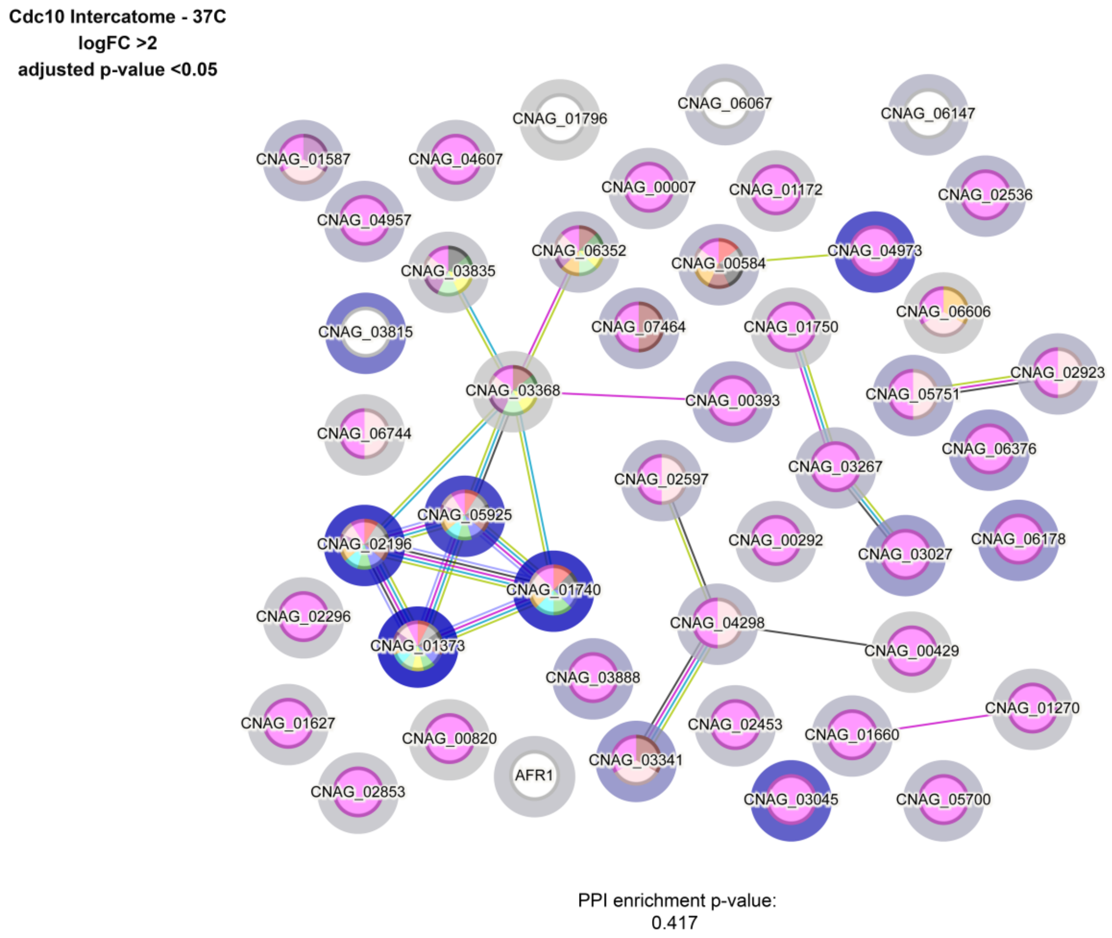

	Biological Process GO Term	Strength	FDR
	Cytoskeletal-dependent cytokinesis	1.49	0.0024
	Mitotic Cytokinesis	1.43	0.0103
	Cell Division	0.94	0.0283
	Mitotic cell cycle process	0.89	0.0070

	Molecular Function GO Term	Strength	FDR
	Microtubule plus-end binding	1.64	0.0347

	Cellular Component GO Term	Strength	FDR
	Septin complex	2.21	6.47e-05
	Septin ring	1.81	0.00031
	Spindle	1.15	0.0204
	Microtubule	1.08	0.0321
	Cell division site	1.07	0.0321
	Cell cortex	1.02	0.00091
	Microtubule cytoskeleton	0.97	0.0015
	Intracellular non-membrane bound organelle	0.43	0.0153
	Intracellular anatomical structure	0.13	0.0321

S10Fig. STRING protein-protein interaction (PPI) network of 46 proteins that are significantly enriched for Cdc10 pulldown versus control, during heat stress (Supplementary Table 2). The PPI network nodes represent proteins, and are color coded according to STRING gene set analysis functional enrichment (Gene Ontology Analysis). The blue halo around each node represents the logFC enrichment value. The darker the halo, the higher the enrichment. The edges

represent protein-protein associations: curated databases (light blue), magenta (experimentally determined), gene neighborhood (green), gene fusions (red), gene co-occurrence (dark blue), text mining (lime green), co-expression (black), and protein homology (light purple).

S11Fig.

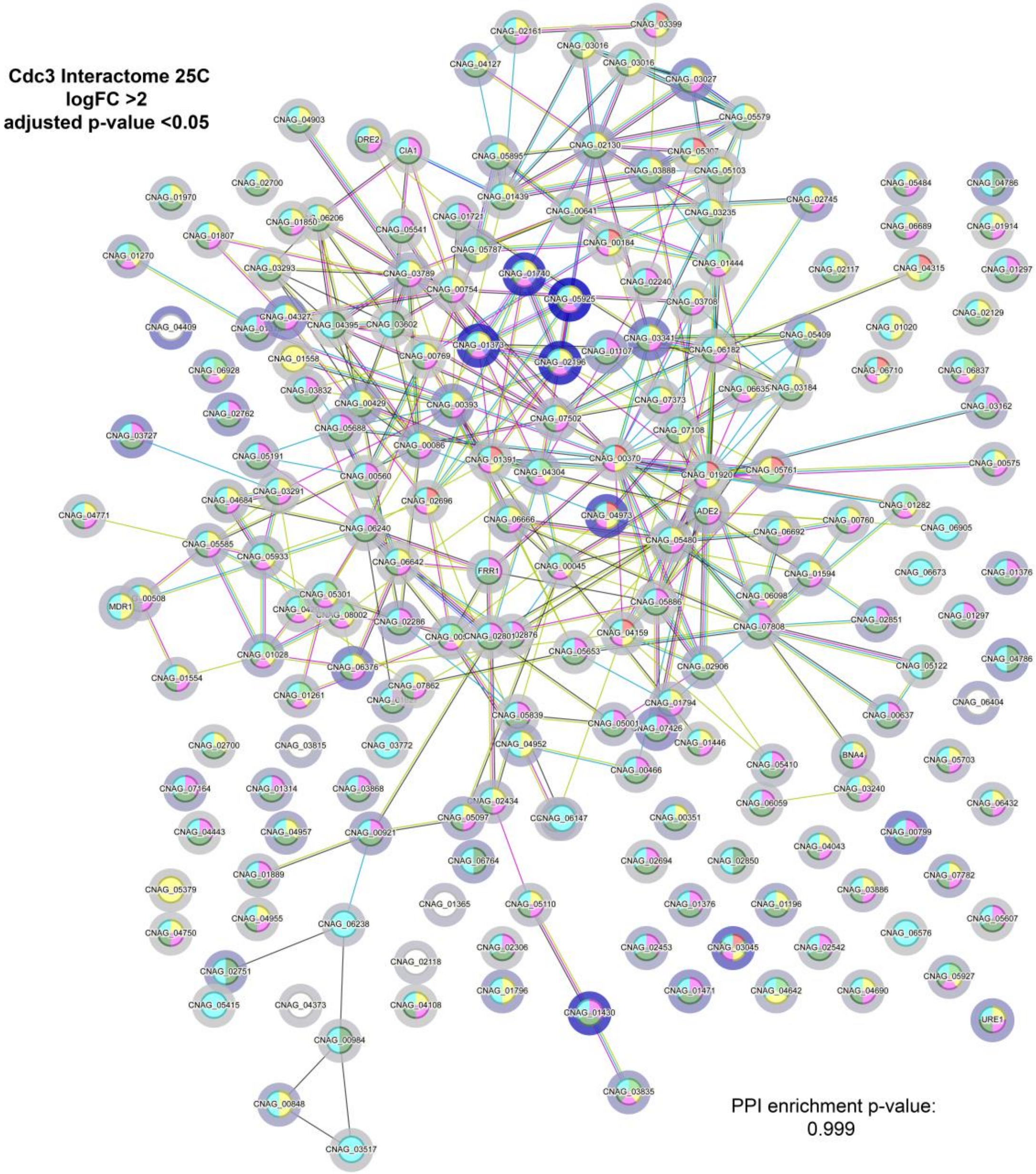

	Molecular Function GO Term	Strength	FDR
	Enzyme binding	0.57	0.0384
	Protein binding	0.31	0.0321
	Binding	0.13	0.0035

	Cellular Component GO Term	Strength	FDR
	Septin Complex	1.58	0.0053
	Cytoplasm	0.16	1.79e-06
	Intracellular anatomical structure	0.12	8.50e-09
	Cellular anatomical entity	0.07	7.42e-06

S11Fig. STRING protein-protein interaction (PPI) network of 196 proteins that are significantly enriched for Cdc3 pulldown at ambient temperature versus control (Supplementary Table

3). The PPI network nodes represent proteins, and are color coded according to STRING gene set analysis functional enrichment (Gene Ontology- Molecular Function & Cellular Component). The blue halo around each node represents the logFC enrichment value. The darker the halo, the higher the enrichment. The edges represent protein-protein associations: curated databases (light blue), magenta (experimentally determined), gene neighborhood (green), gene fusions (red), gene co-occurrence (dark blue), text mining (lime green), co-expression (black), and protein homology (light purple).

S12Fig.

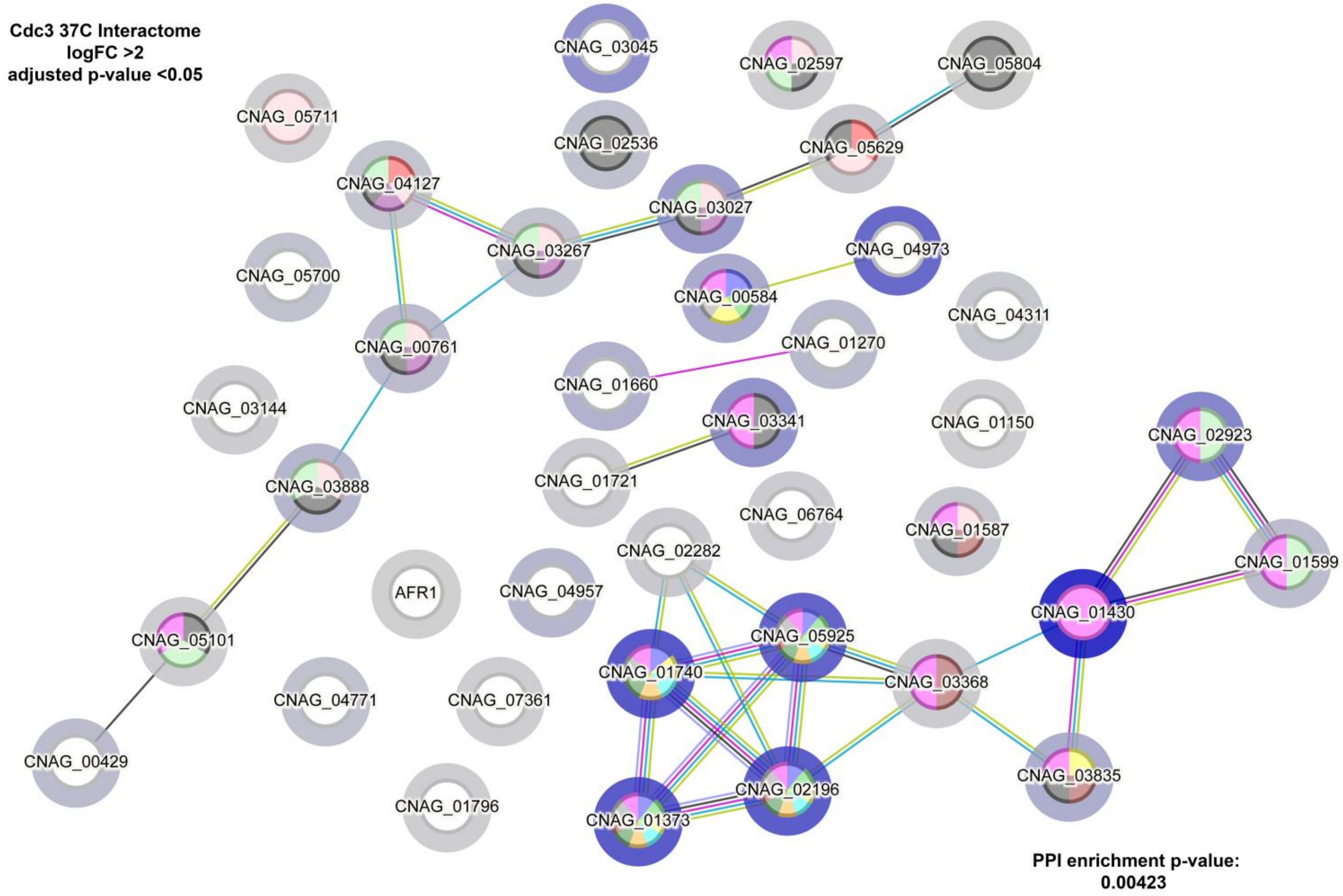

	Biological Process GO Term	Strength	FDR
	snoRNA splicing	2.27	0.0327
	Cytoskeleton-dependent cytokinesis	1.55	0.0016
	Mitotic cytokinesis	1.49	0.0081
	Cell division	1.0	0.0126
	mRNA processing	0.89	0.0021

	Cellular Component GO Term	Strength	FDR
	Septin complex	2.27	3.79e-05
	Septin ring	1.87	0.00027
	U2-type spliceosomal complex	1.18	0.0167
	Cell division site	1.13	0.0225
	Microtubule cytoskeleton	0.96	0.0057
	Cell cortex	0.93	0.0254
	Nuclear protein-containing complex	0.62	0.0011
	Ribonucleoprotein complex	0.58	0.0314
	Intracellular non-membrane-bounded organelle	0.43	0.0254

S12Fig. STRING protein-protein interaction (PPI) network of 40 proteins that are significantly enriched for Cdc3 pulldown versus control, during heat stress (Supplementary Table 4). The PPI network nodes represent proteins, and are color coded according to STRING gene set analysis functional enrichment (Gene Ontology- Biological Process & Cellular component). The blue halo around each node represents the logFC enrichment value. The darker the halo, the higher the

enrichment. The edges represent protein-protein associations: curated databases (light blue), magenta (experimentally determined), gene neighborhood (green), gene fusions (red), gene co-occurrence (dark blue), text mining (lime green), co-expression (black), and protein homology (light purple).

S13Fig.

A.

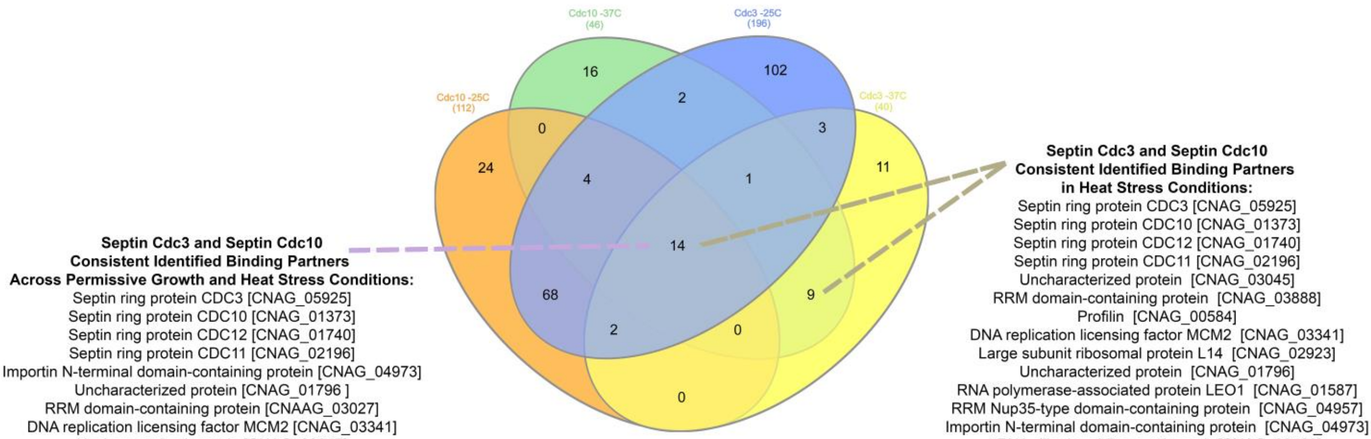

Septin Cdc3 and Septin Cdc10
Consistent Identified Binding Partners
Across Permissive Growth and Heat Stress Conditions:

Septin ring protein CDC3 [CNAG_05925]
Septin ring protein CDC10 [CNAG_01373]
Septin ring protein CDC12 [CNAG_01740]
Septin ring protein CDC11 [CNAG_02196]
Importin N-terminal domain-containing protein [CNAG_04973]
Uncharacterized protein [CNAG_01796]
RRM domain-containing protein [CNAAG_03027]
DNA replication licensing factor MCM2 [CNAG_03341]
Uncharacterized protein [CNAG_03045]
PWI domain-containing protein [CNAG_03027]
tRNA-dihydrouridine synthase 1 [CNAG_00429]
RRM Nup35-type domain-containing protein [CNAG_04957]
Profilin [CNAG_00584]
DASH complex subunit DAD3

Septin Cdc3 and Septin Cdc10
Consistent Identified Binding Partners
in Heat Stress Conditions:

Septin ring protein CDC3 [CNAG_05925]
Septin ring protein CDC10 [CNAG_01373]
Septin ring protein CDC12 [CNAG_01740]
Septin ring protein CDC11 [CNAG_02196]
Uncharacterized protein [CNAG_03045]
RRM domain-containing protein [CNAG_03888]
Profilin [CNAG_00584]
DNA replication licensing factor MCM2 [CNAG_03341]
Large subunit ribosomal protein L14 [CNAG_02923]
Uncharacterized protein [CNAG_01796]
RNA polymerase-associated protein LEO1 [CNAG_01587]
RRM Nup35-type domain-containing protein [CNAG_04957]
Importin N-terminal domain-containing protein [CNAG_04973]
tRNA-dihydrouridine synthase 1 [CNAG_00429]
Transcription initiation factor TFIID subunit 6 [CNAG_02536]
Microtubule Associated protein [CNAG_03368]
DASH complex subunit DAD3 [CNAG_03835]
ABC multidrug transporter AFR1 [CNAG_00730]
PWI domain-containing protein [CNAG_03027]
Endoplasmic reticulum protein [CNAG_05700]
Splicing factor 3B subunit 2 [CNAG_03267]
Uncharacterized protein [CNAG_02597]
(Translation initiation factor) eIF-2B GDP-GTP exchange factor subunit alpha [CNAG_01660]

B.

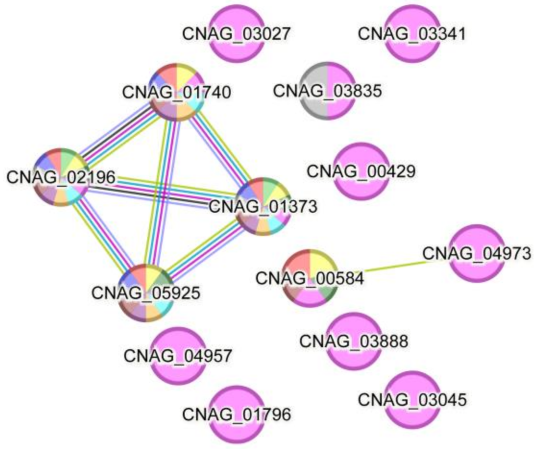

PPI enrichment p-value: 0.00271

	STRING GO Term	Strength	FDR
	Septin ring organization	2.07	0.0432
	Cytoskeleton-dependent cytokinesis	2.01	5.44e-06
	Mitotic cytokinesis	1.95	0.00012
	Binding	0.33	0.0317
	Septin complex	2.73	4.26e-07
	Septin ring	2.33	3.03e-06
	Cell division site	1.59	0.00044
	Cell cortex	1.39	0.00027
	Microtubule cytoskeleton	1.24	0.0074

	Local network cluster (STRING)	Strength	FDR
	Septin complex	2.63	1.32e-06
	Cell cortex, and MAPK signaling pathway - yeast	1.25	0.0027

C.

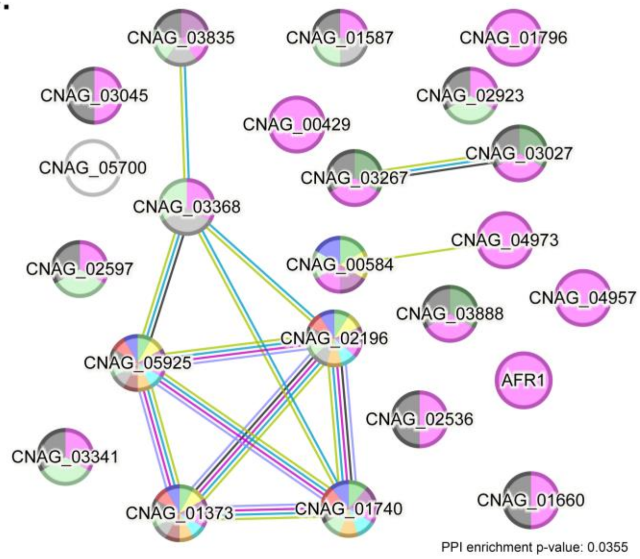

PPI enrichment p-value: 0.0355

	STRING GO Term	Strength	FDR
	Cytoskeleton-dependent cytokinesis	1.79	8.86e-05
	Mitotic cytokinesis	1.73	0.00060
	Spliceosomal complex assembly	1.57	0.0233
	Cell division	1.24	0.00056
	Binding	0.31	0.00091
	Septin Complex	2.51	3.47e-06
	Septin Ring	2.11	2.64e-05
	Intracellular immature spore	1.77	0.0405
	Microtubule cytoskeleton	1.21	0.00027
	Intracellular non-membrane-bounded organelle	0.57	0.0046
	Protein-containing complex	0.45	0.0027

	Local network cluster (STRING)	Strength	FDR
	Septin complex	2.41	1.15e-05
	Cell cortex, and MAPK signaling pathway - yeast	1.04	0.0393

S13Fig. STRING Analysis of Overlap between Cdc10 and Cdc3 protein interactome. Significant interacting partners were determined by statistical t-test using logFC >2 and adjusted p-

value of 0.05. (A) Venn Diagram showing the overlap of significantly enriched proteins for co-immunoprecipitation of both Cdc3 and Cdc10 between ambient temperature (~25°C) and heat stress (37°C). (B) STRING analysis generated protein-protein interaction (PPI) network of the 14 proteins identified consistently as part of both the Cdc3 and Cdc10 interactome with a significant enrichment during both ambient temperature and heat stress. (C) STRING analysis generated protein-protein interaction (PPI) network of the 25 proteins identified consistently as part of both the Cdc3 and Cdc10 interactome with a significant enrichment during heat stress. The PPI network nodes represent proteins, and are color coded according to STRING gene set analysis functional enrichment. The edges represent protein-protein associations: curated databases (light blue), magenta (experimentally determined), gene neighborhood (green), gene fusions (red), gene co-occurrence (dark blue), text mining (lime green), co-expression (black), and protein homology (light purple).
