# Supplementary figures and images for: Quantitative analysis of septin Cdc10 & Cdc3-associated proteome during stress response in the fungal pathogen Cryptococcus neoformans
Source: PLoS One. 2024 Dec 17;19(12):e0313444. doi: 10.1371/journal.pone.0313444 (PMC11651612; doi:10.1371/journal.pone.0313444)

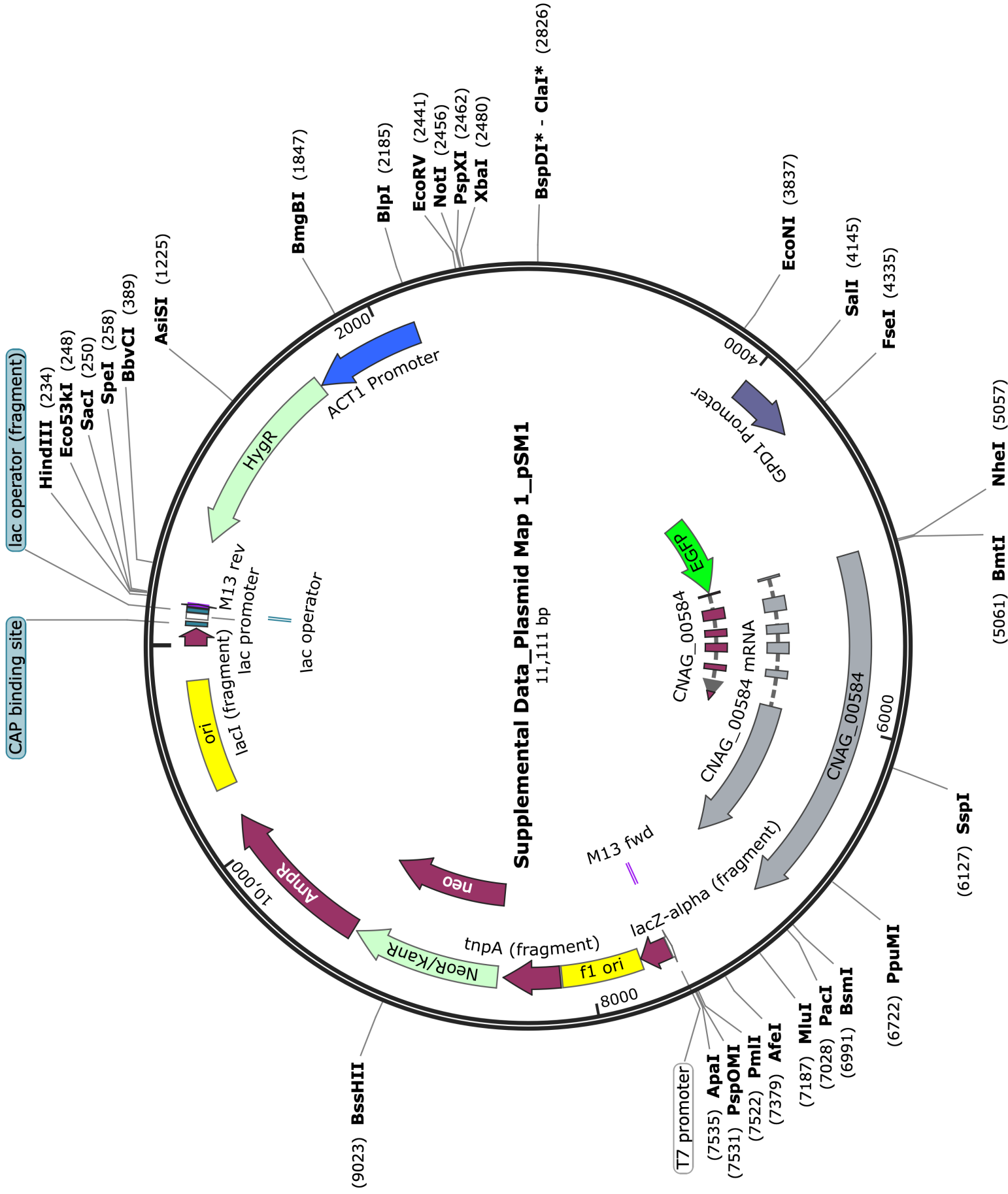

Supplement: S1 Data — (PDF) [file pone.0313444.s006.pdf]

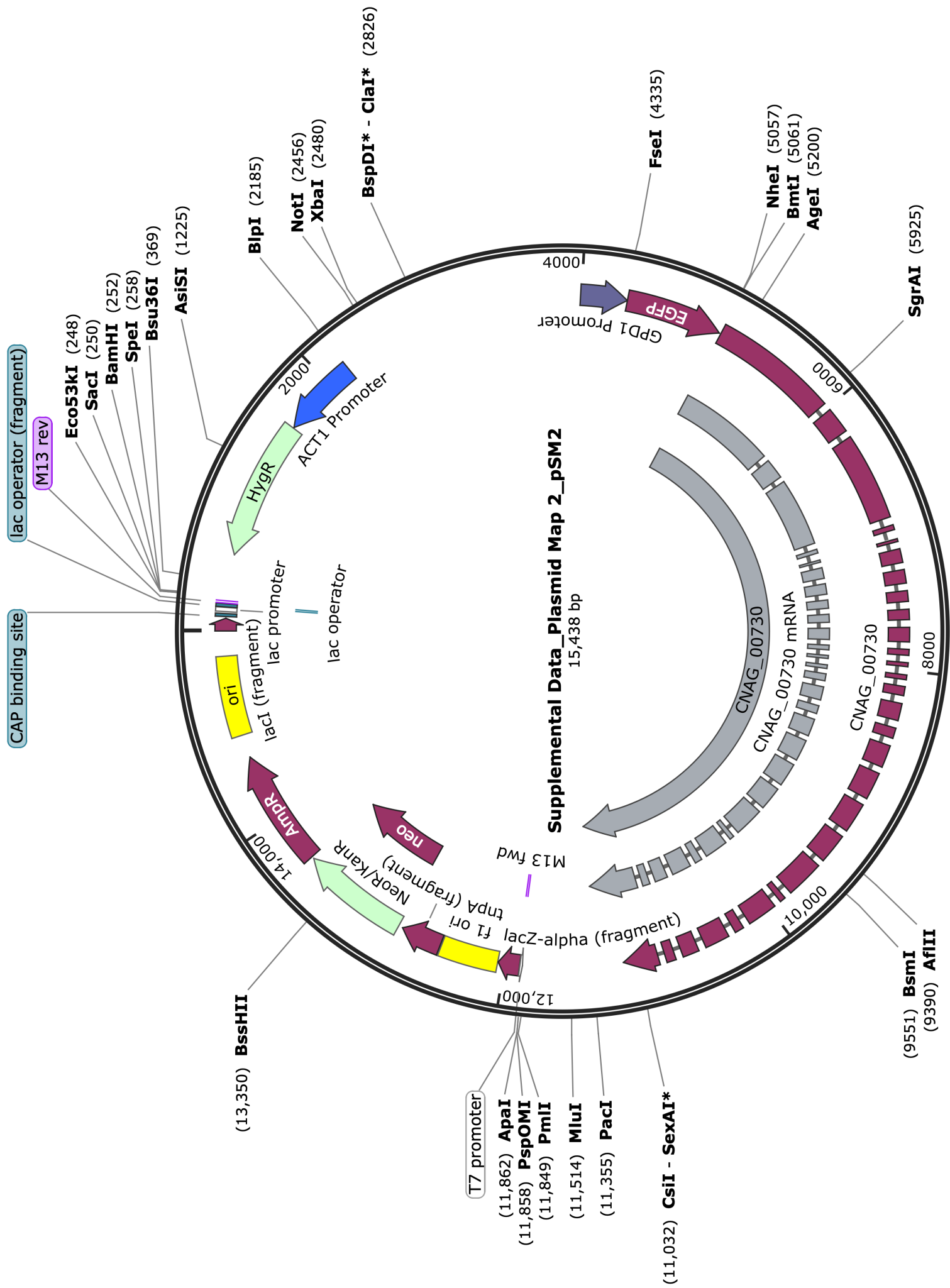

Supplement: S2 Data — (PDF) [file pone.0313444.s007.pdf]
