# Supplementary material for: Quantitative analysis of septin Cdc10 & Cdc3-associated proteome during stress response in the fungal pathogen Cryptococcus neoformans
Source: PLoS One. 2024 Dec 17;19(12):e0313444. doi: 10.1371/journal.pone.0313444 (PMC11651612; doi:10.1371/journal.pone.0313444)
Supplement: S1 Raw images — (PDF) [file pone.0313444.s009.pdf]

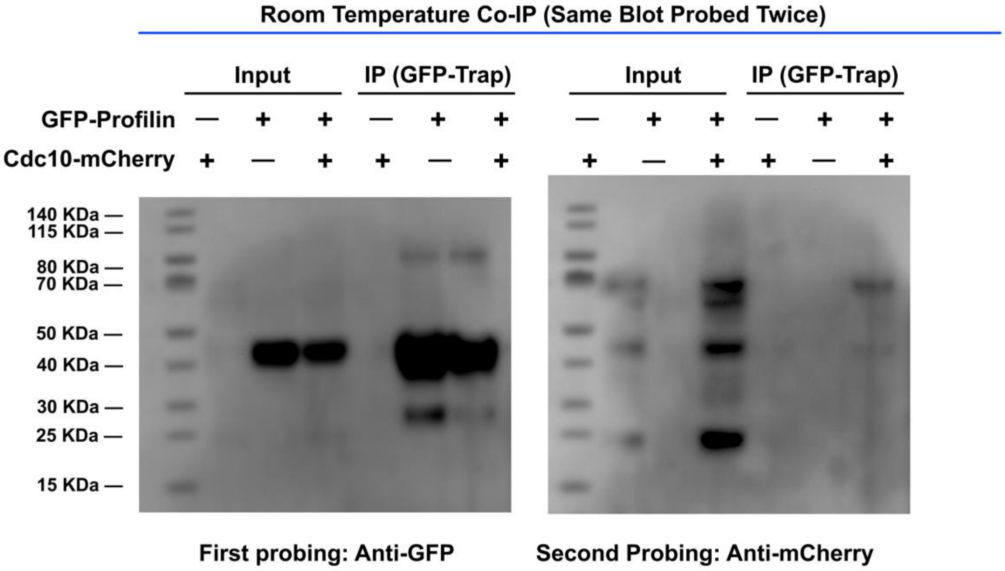
Room Temperature Co-IP (Same Blot Probed Twice)
Input
IP (GFP-Trap)
Input
IP (GFP-Trap)
GFP-Profilin — + + — + + — + + — + +
Cdc10-mCherry + — + + — + + — + + — +
140 KDa —
115 KDa —
80 KDa —
70 KDa —
50 KDa —
40 KDa —
30 KDa —
25 KDa —
15 KDa —
First probing: Anti-GFP
Second Probing: Anti-mCherry

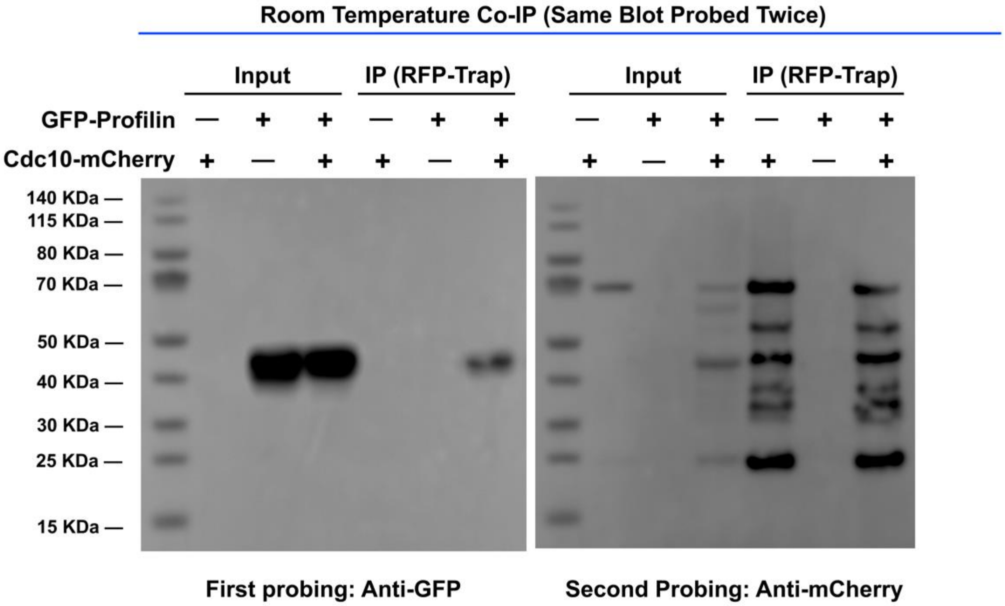
Room Temperature Co-IP (Same Blot Probed Twice)
Input
IP (RFP-Trap)
Input
IP (RFP-Trap)
GFP-Profilin — + + — + + — + + — + +
Cdc10-mCherry + — + + — + + — + + — +
140 KDa —
115 KDa —
80 KDa —
70 KDa —
50 KDa —
40 KDa —
30 KDa —
25 KDa —
15 KDa —
First probing: Anti-GFP
Second Probing: Anti-mCherry

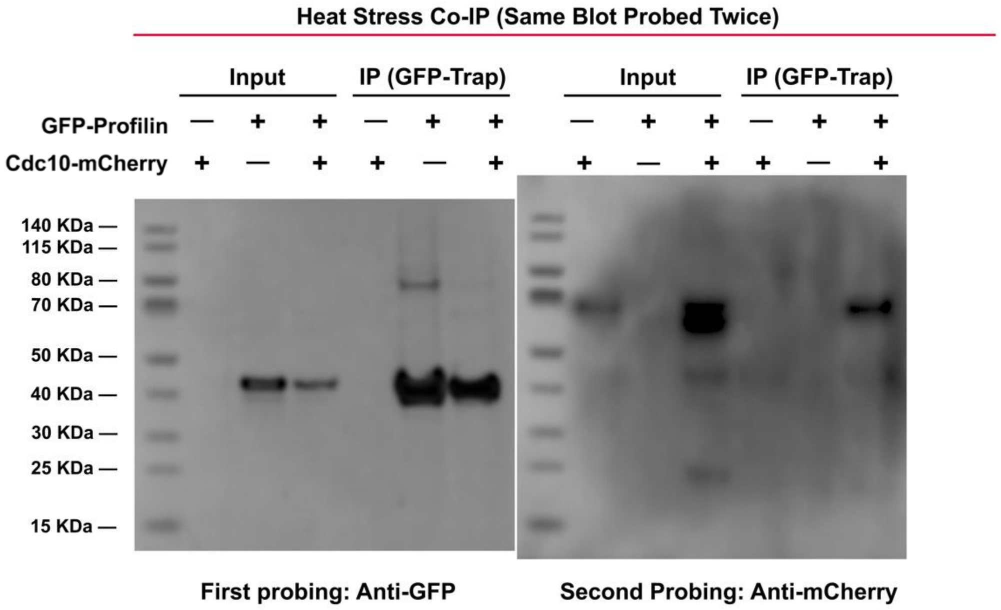
Heat Stress Co-IP (Same Blot Probed Twice)
Input
IP (GFP-Trap)
Input
IP (GFP-Trap)
GFP-Profilin — + + — + + — + + — + +
Cdc10-mCherry + — + + — + + — + + — +
140 KDa —
115 KDa —
80 KDa —
70 KDa —
50 KDa —
40 KDa —
30 KDa —
25 KDa —
15 KDa —
First probing: Anti-GFP
Second Probing: Anti-mCherry

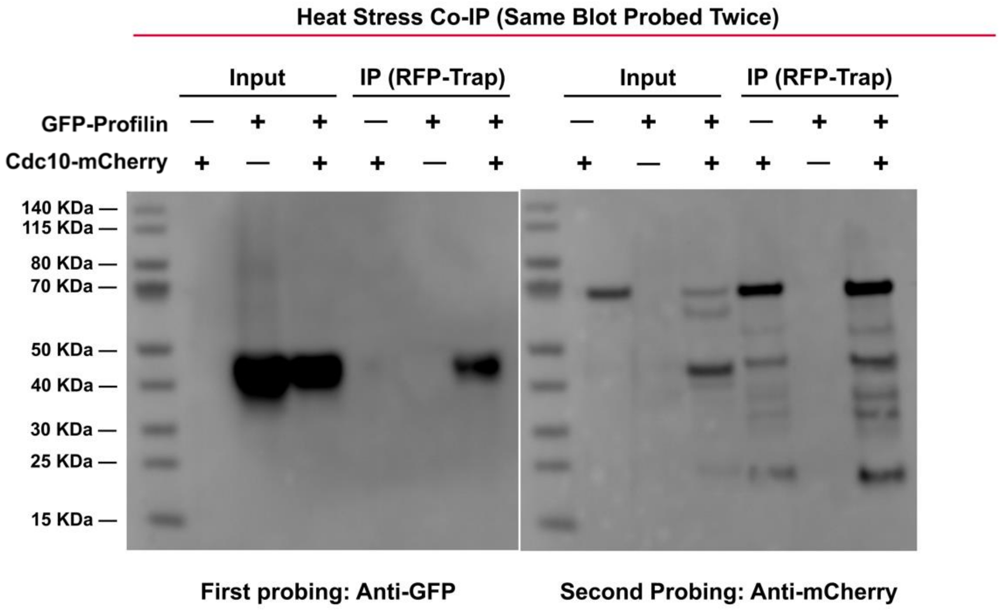
Heat Stress Co-IP (Same Blot Probed Twice)
Input
IP (RFP-Trap)
Input
IP (RFP-Trap)
GFP-Profilin — + + — + + — + + — + +
Cdc10-mCherry + — + + — + + — + + — +
140 KDa —
115 KDa —
80 KDa —
70 KDa —
50 KDa —
40 KDa —
30 KDa —
25 KDa —
15 KDa —
First probing: Anti-GFP
Second Probing: Anti-mCherry

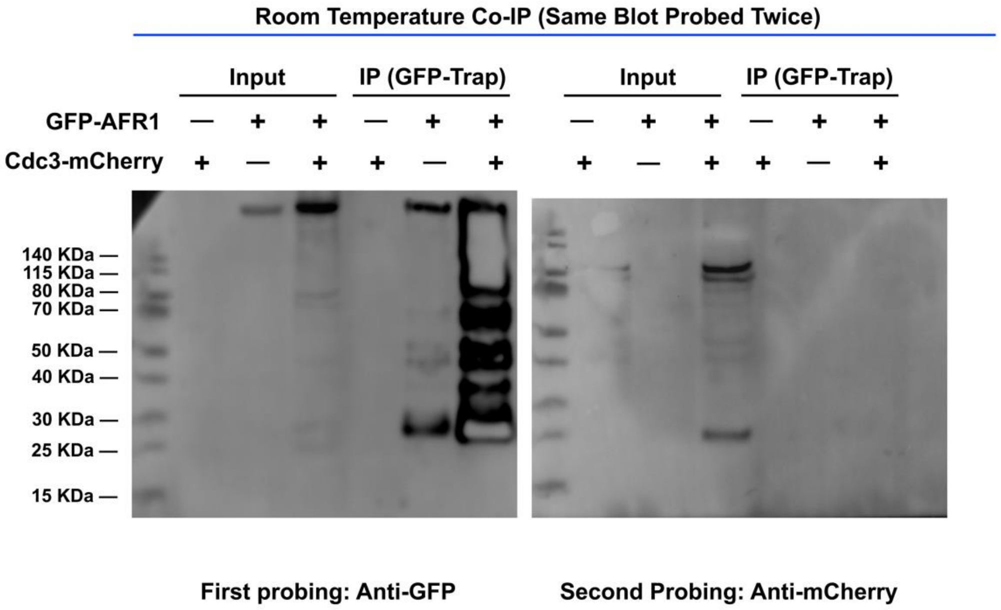

Room Temperature Co-IP (Same Blot Probed Twice)
Input
IP (GFP-Trap)
Input
IP (GFP-Trap)
GFP-AFR1 — + + — + + — + + — + +
Cdc3-mCherry + — + + — + + — + + — +
140 KDa —
115 KDa —
80 KDa —
70 KDa —
50 KDa —
40 KDa —
30 KDa —
25 KDa —
15 KDa —
First probing: Anti-GFP
Second Probing: Anti-mCherry

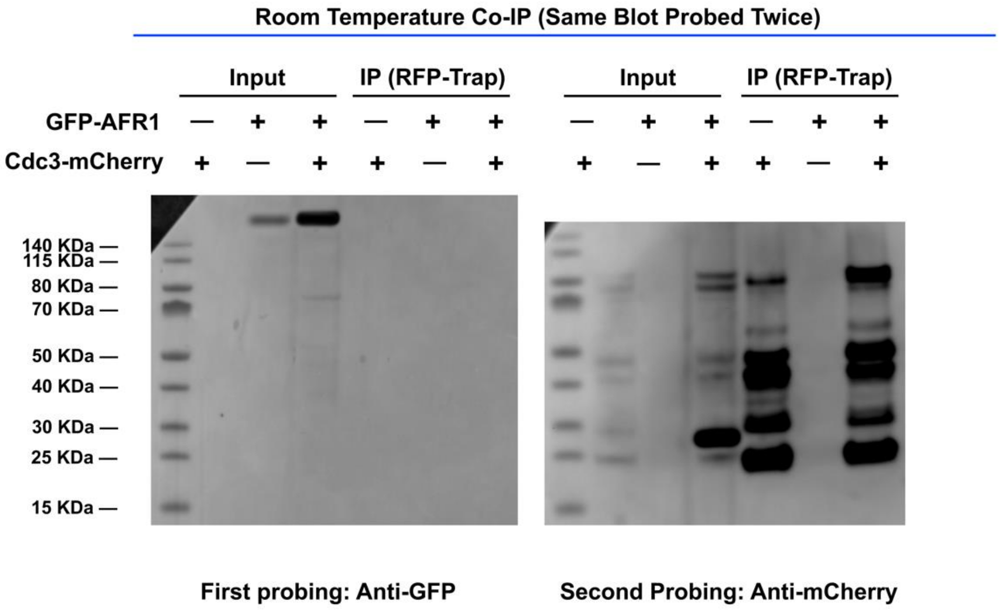

Room Temperature Co-IP (Same Blot Probed Twice)
Input
IP (RFP-Trap)
Input
IP (RFP-Trap)
GFP-AFR1 — + + — + + — + + — + +
Cdc3-mCherry + — + + — + + — + + — +
140 KDa —
115 KDa —
80 KDa —
70 KDa —
50 KDa —
40 KDa —
30 KDa —
25 KDa —
15 KDa —
First probing: Anti-GFP
Second Probing: Anti-mCherry

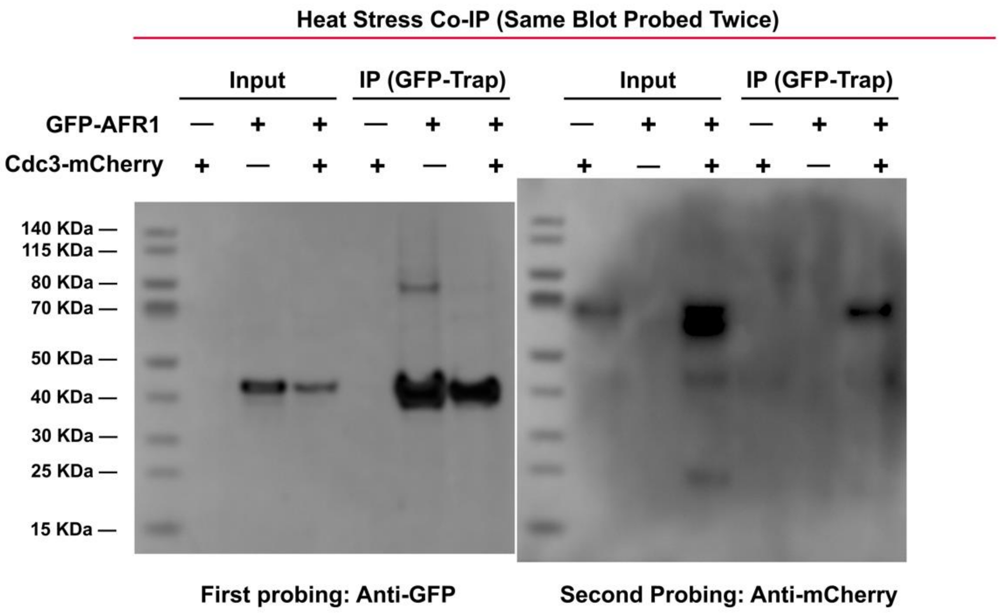

Heat Stress Co-IP (Same Blot Probed Twice)
Input
IP (GFP-Trap)
Input
IP (GFP-Trap)
GFP-AFR1 — + + — + + — + + — + +
Cdc3-mCherry + — + + — + + — + + — +
140 KDa —
115 KDa —
80 KDa —
70 KDa —
50 KDa —
40 KDa —
30 KDa —
25 KDa —
15 KDa —
First probing: Anti-GFP
Second Probing: Anti-mCherry

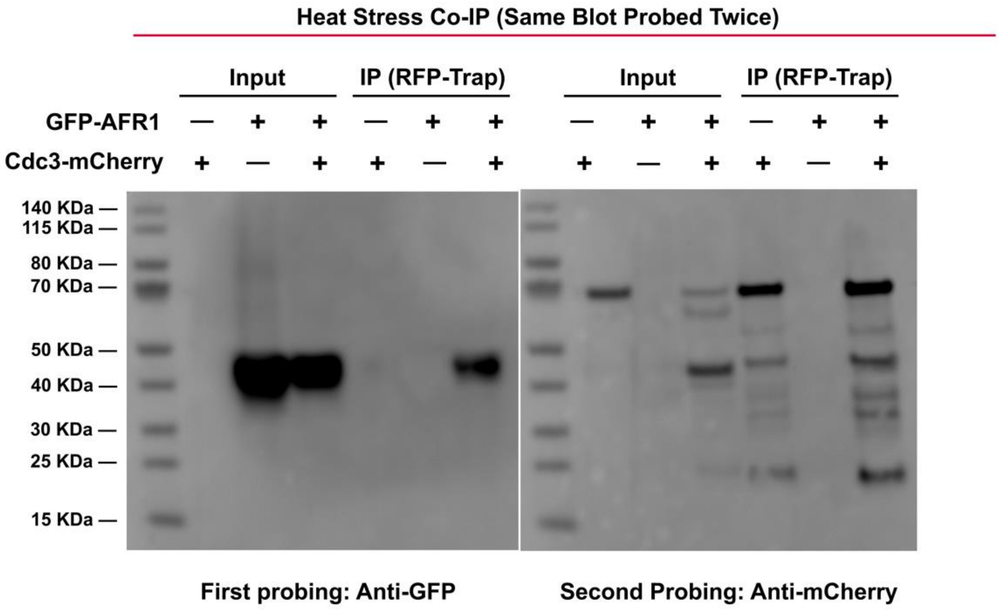

Heat Stress Co-IP (Same Blot Probed Twice)
Input
IP (RFP-Trap)
Input
IP (RFP-Trap)
GFP-AFR1 — + + — + + — + + — + +
Cdc3-mCherry + — + + — + + — + + — +
140 KDa —
115 KDa —
80 KDa —
70 KDa —
50 KDa —
40 KDa —
30 KDa —
25 KDa —
15 KDa —
First probing: Anti-GFP
Second Probing: Anti-mCherry
